# Whole-genome resequencing of honeybee drones to detect genomic selection in a population managed for royal jelly

**DOI:** 10.1038/srep27168

**Published:** 2016-06-03

**Authors:** David Wragg, Maria Marti-Marimon, Benjamin Basso, Jean-Pierre Bidanel, Emmanuelle Labarthe, Olivier Bouchez, Yves Le Conte, Alain Vignal

**Affiliations:** 1 GenPhySE, Université de Toulouse, INRA, INPT, INP-ENVT, 31326 Castanet Tolosan, France; 2 Institut de l’abeille (ITSAP), UMT PrADE, 8914 Avignon, France; 3 GABI, INRA, AgroParisTech, Université Paris-Saclay, 78352 Jouy-en-Josas, France; 4 INRA, UR 406 Abeilles et Environnement, UMT PrADE, 84914 Avignon, France

## Abstract

Four main evolutionary lineages of *A. mellifera* have been described including eastern Europe (C) and western and northern Europe (M). Many apiculturists prefer bees from the C lineage due to their docility and high productivity. In France, the routine importation of bees from the C lineage has resulted in the widespread admixture of bees from the M lineage. The haplodiploid nature of the honeybee *Apis mellifera*, and its small genome size, permits affordable and extensive genomics studies. As a pilot study of a larger project to characterise French honeybee populations, we sequenced 60 drones sampled from two commercial populations managed for the production of honey and royal jelly. Results indicate a C lineage origin, whilst mitochondrial analysis suggests two drones originated from the O lineage. Analysis of heterozygous SNPs identified potential copy number variants near to genes encoding odorant binding proteins and several cytochrome P450 genes. Signatures of selection were detected using the hapFLK haplotype-based method, revealing several regions under putative selection for royal jelly production. The framework developed during this study will be applied to a broader sampling regime, allowing the genetic diversity of French honeybees to be characterised in detail.

The earliest evidence of human interaction with honeybees dates to around 7 thousand years ago (kya), and is illustrated in a cave painting (Araña Caves, Spain) depicting a figure collecting honey from a bee nest in a tree. Later evidence of domestication can be found in Israel where a well organized apiary at Tel Rehov dating to approximately 3 kya was discovered^1^. The Tel Rehov hives were found to contain the exceptionally well preserved remains of honeybee workers, drones, pupae and larvae, which differ morphologically from the present day local subspecies (*A. m. syriaca*) and more closely resemble *A. m. anatoliaca* which currently inhabits parts of Turkey^2^. *A. mellifera* includes 27 geographic races or subspecies, each defined according to the morphological, behavioural, physiological and ecological characteristics suited to their local habitat^3^. These broadly represent distinct evolutionary lineages distributed throughout Africa (A), western and northern Europe (M), eastern Europe (C), the Near East and central Asia (O), and Yemen and Ethiopia (Y), having diverged around 150–300 kya ago^4^,^5^,^6^,^7^. In France, the endogenous subspecies is *A. m. mellifera* (lineage M), however since the late 19^th^ century apiculturists have increasingly imported *A. m. ligustica* (lineage C)^4^. Honeybees of this subspecies are preferred because of their ability to store large quantities of honey without swarming and their docile nature if disturbed^8^. This has resulted in varying degrees of admixture and a significant contribution of C lineage alleles in domestic bees - a consequence of apicultural practices not only in France, but throughout much of the world^4^.

Despite being managed for over 3,000 years, selective breeding programmes have witnessed limited long-term genetic gains in honeybees^9^, although not unexpectedly so. Apicultural practices commonly include (i) using naturally mated queens who fly through congregation areas attracting drones from hundreds of surrounding colonies, where they typically mate with 7–20 drones^10^, and (ii) replacing queens every 1–2 years to maximize productivity^11^. The regular importation of stock and movement of managed colonies around large areas, together with the promiscuous mating system of honeybees, have exposed native European honeybees to introgressive hybridisation with managed non-native subspecies^12^,^13^. The resulting increase in genetic diversity of managed populations^14^,^15^ reduces inbreeding and the overall effectiveness of selective breeding programmes. Replacing local queens with imported queens also intuitively undermines selective breeding programmes, as the queens’ breeders may have selected for different traits, and the agro-ecological conditions in which the queens were raised may differ substantially to those at her destination^16^. As a consequence, the conservation of local honeybee subspecies and their genetic identity is a major concern in several countries^16^. *A. m. mellifera* in particular is increasingly threatened in its native range, prompting the establishment of several conservation programmes throughout western Europe^17^.

Domestication characteristically results in different breeds or lines of a species with exaggerated traits of interest; the products of extensive breeding efforts which can lead to high levels of inbreeding and genetic isolation^18^,^19^. Some apiculturists specialise in queen breeding, distributing large numbers of progeny from few queen mothers, leading to a decrease in genetic diversity^20^ suggested as one of several causes of global decline in honeybees^21^. This is of particular importance as genetic diversity has been associated with resistance to parasites^22^, nest thermoregulation^23^ and colony defence^24^. In contrast, a comparison of two heavily managed commercial populations to several “progenitor” populations which included feral colonies from Africa (*A. m. scutellata*) and managed colonies from Europe (*A. m. mellifera, A. m. iberiensis* and *A. m. carnica*), lead Harpur *et al*. to the controversial conclusion that management increases genetic diversity via admixture^14^. Several concerns were raised by De la Rúa *et al*. regarding the conclusions of that study^13^, and although it is acknowledged that admixture can increase genetic diversity in the short term, it ultimately results in genetic homogenization of the admixed population, loss of local adaptations, and risk of genetic pollution to native subspecies through gene flow. Harpur *et al*. responded to these concerns^15^ providing justification for their processing of the data, supporting literature concerning evidence of admixture, and additional analyses supporting their initial findings – that movement and interbreeding of structured populations results in increased genetic diversity of progeny relative to progenitor populations. Nonetheless, Harpur *et al*. are in agreement with De la Rúa *et al*. that native subspecies should be conserved.

Admixture and inbreeding are not the only concerns related to managed domestic breeds, as selection on specific traits can affect the genome locally around the genes involved in the trait’s expression, leading to selection signatures. These are usually chromosomal segments exhibiting a localised loss of heterozygosity in the selected population and/or an increased differentiation to other populations. Current studies on honeybees only rarely take this into account. In order to understand the genomic make-up of French managed honeybee populations, the SeqApiPop project, a collaboration between the Institut National de la Recherche Agronomique (INRA) and the Institut de l’abeille (ITSAP), aims to sequence complete genomes of several hundred honeybees. A similarly extensive study involving diploid organisms with large genomes, such as humans or livestock species, would entail considerable economic cost due to the need for high depths of coverage to circumvent issues arising from heterozygosity. However, the haploid nature of *A. mellifera* drones and their small genome size (<250 Mb), makes such a study both economically and computationally feasible. Here, as a pilot study, we present the preliminary analyses of whole-genome re-sequencing data of 120 drones drawn from five populations, chosen in order to assess their genetic make-up and to detect signatures of selection in a population selected for the production of royal jelly. These include the royal jelly population derived from lineage C, three further lineage C populations from different geographic origins and management regimes, and a population from lineage M to function as an outgroup.

## Results

### Sequencing and read mapping

To calculate the optimal economic sequencing depth, two drones (H1 and H2) were sequenced at moderately high depth of coverage (DP; DP ≥ 15), and reads sequentially down-sampled to approximate sequencing at depths of 10, 7, 5 and 3 X. Regression fitting of DP against fraction of genome callable (GX) indicated DP = 5.3 X ≈ GX = 0.7, and GX ≥ 0.8 requires much higher DP (Supplementary Material). We proceeded to assess the suitability of drones sequenced at DP ≈ 6 for characterising population diversity and detecting signatures of selection. Drones were sampled from one population used for honey production (HN; n = 30) and another for royal jelly (RJ; n = 30).

The sequence data was supplemented with a reference dataset of diploids (n = 39) published by Harpur *et al*.^7^, representing four lineages (A, M, C, Y). This reference dataset enabled the fraction of lineage ancestry in HN and RJ populations to be assessed. On average ≥91% of reads from both datasets mapped to the reference genome, with ≥83% having a mapping quality (MQ) ≥ 20 (Supplementary Table ST1). Mapping duplicates were highest in the reference dataset which is not unexpected given their higher DP (26.61 ± 2.04) compared to the drones (7.1 ± 2.59). The higher coverage in the reference dataset captured an additional 6% of the reference genome compared to the drones, and resulted in a 10% increase in GX (Supplementary Table ST1).

### Variant detection in the drone and reference populations

More than twice the number of SNPs were identified on average per individual in the reference dataset than in the drones (Supplementary Table ST2). Moreover, the numbers of SNPs identified are in agreement with our calculations of SNPs identified in drones sequenced at 5 and 7 X to diploids sequenced at 20 and 30 X (Supplementary Methods SM2). Post-filtering for DP, 10.44 M SNPs were detected in the reference dataset, whilst in the drones it was 3.69 M. The higher number of SNPs in the reference dataset compared to the drones (RJ and HN) is not unexpected given the near 4-fold increase in DP and diversity of lineages sampled. The mean number of SNPs detected per drone (~856 K) is marginally lower than that detected in reference bees from the C lineage (~1 M), from which the drones originate, and is consistent with the modelled expectations (see Supplementary Material). Merging both datasets resulted in 11.24 M unique SNPs, of which 807 K were common to both datasets. The mean fraction of missing genotypes across all samples was 0.02. The greatest number of private SNPs (4.96 M) was detected in lineage A, whilst the least (207 K) was observed in lineage C (Supplementary Fig. SF1). As the reference genome was generated from lineage C individuals it is not unsurprising to observe fewer SNPs in bees of the same lineage. For downstream analysis three datasets were constructed, the first (i) comprised all drones (HN and RJ), the second (ii) comprised reference individuals and drones (HN and RJ), whilst the third (iii) was composed of reference individuals and *in silico* diploids generated by combining the alleles of random pairs of drones within each population. Individuals were genotyped for all SNPs detected and, following quality control and imputation, 2.53 M SNPs were retained in dataset (i), 3.12 M SNPs in dataset (ii), and 3.16 M SNPs in dataset (iii).

For each drone population (dataset i), SNPs in which at least 10% of the population carried a non-reference allele were evaluated using Ensembl’s variant effect predictor (VEP). This enables the quantification of variants by genomic location (e.g. upstream of a transcript, in coding sequence, etc.) and consequence (e.g. frameshift, missense mutation, etc.), enabling one to consider the potential effects on the fitness of mutations. Intergenic variants accounted for the largest number of predicted effects (59.5%, inclusive of 16.3% upstream and 15.8% downstream variants), followed by intron (36.8%), exon (3.5%) and splicing (0.2%) variants (Supplementary Table ST3). The same distribution (to within <0.63%) was observed amongst the SNPs private to each population. These results are not unexpected given that exon sequences make up <12% of the genome, and that mutations within exons and splicing regions are more likely to be deleterious than those in introns and intergenic sequences.

### Diversity and population structure

Nucleotide diversity (π) was calculated for each evolutionary lineage and drone population (dataset ii). Diversity was observed to be highest in lineage A (π = 0.0058), followed by HN bees (π = 0.0036), lineage Y (π = 0.0033), RJ bees (π = 0.0032), M (π = 0.0029) and C lineage (π = 0.0024). Plots of autosomal nucleotide diversity in 12 kb bins for HN and RJ bees are available in the Supplementary material (Supplementary Figs SF2 to SF17).

**Figure 1:**
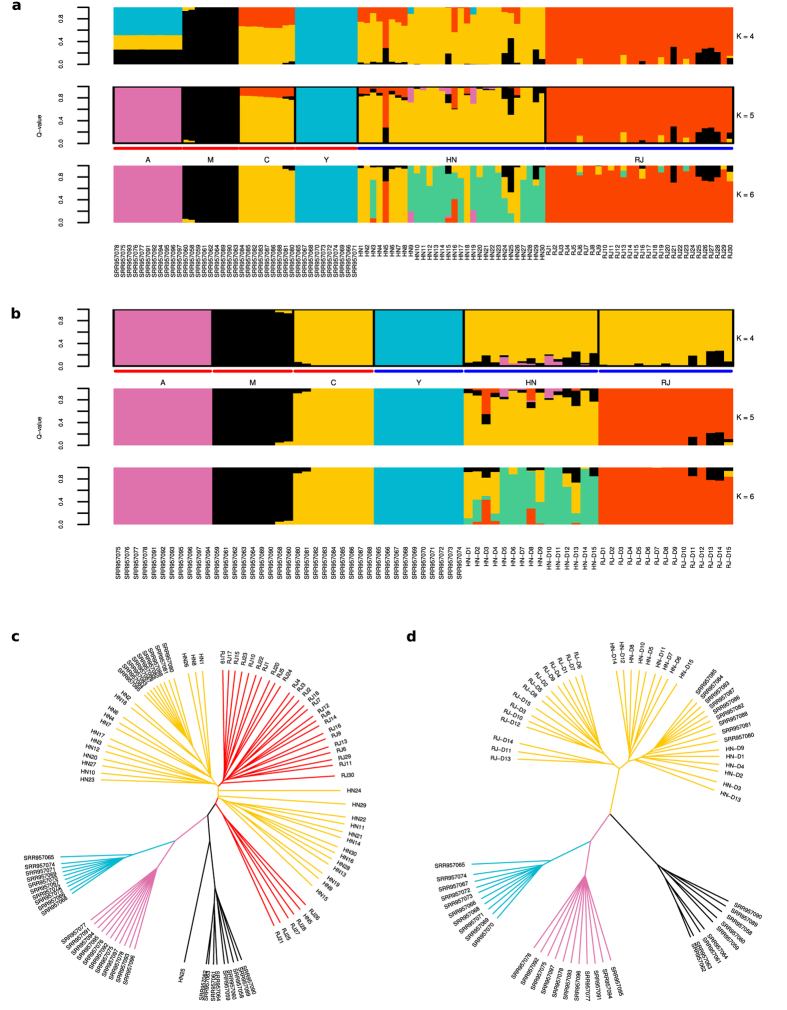
Population structure of HN and RJ drones to workers from lineages A, M, C and Y. (**a**) ADMIXTURE results of 4 ≤ K ≤ 6 for reference bees from four evolutionary lineages (A, M, C, Y) and drones from populations selected for honey (HN) and royal jelly production (RJ). The optimal K (5) is indicated by the additional population annotation. (**b**) ADMIXTURE results of reference bees and *in silico* diploids (see Supplementary Material). (**c**) Unrooted phylogeny constructed using the genotype matrix of the optimal K identified in plot A. (**d**) Unrooted phylogeny constructed using the genotype matrix of the optimal K identified in plot B.

Combining the diploid reference dataset of four lineages (A, M, C, Y) with our haploid data, which exhibits systematic homozygosity, could potentially result in misleading relationships in ADMIXTURE analysis. To test this, we compared ADMIXTURE analyses using a mixed haploid-diploid dataset with those obtained with a diploid-only dataset by combining *in silico* alleles from pairs of drones to create diploid individuals. The optimal number of ancestral backgrounds (*K*) was inferred from the cross-validation (CV) error estimation (Supplementary Fig. SF18). Following 10 replicates for 1 ≤ *K* ≤ 6 the optimal was *K* = 5 in the haploid-diploid dataset (ii; Fig. 1a; Supplementary Table ST4), whilst it was *K* = 4 in the diploid-only dataset (iii; Fig. 1b; Supplementary Table ST5). The ancestral backgrounds identified coincide with the four lineages (A, M, C, Y) in the reference dataset. A fifth background dominated by RJ bees is present in the haploid-diploid dataset (ii), and emerges at *K* = 5 in the diploid dataset (iii), whilst at *K* = 6 both analyses are comparable. Unrooted phylogenetic trees constructed from the haploid-diploid (ii; Fig. 1c) and diploid datasets (iii; Fig. 1d) broadly support the ADMIXTURE results. The *F*_*ST*_ values at the optimal *K* between populations in the diploid dataset (iii) ranged from 0.381 when comparing A to C, to 0.61 when comparing Y to M (Supplementary Table ST6), whilst those in the haploid-diploid dataset (ii) ranged from 0.139 when comparing C to RJ, to 0.625 when comparing Y to M (Supplementary Table ST7).

**Figure 2:**
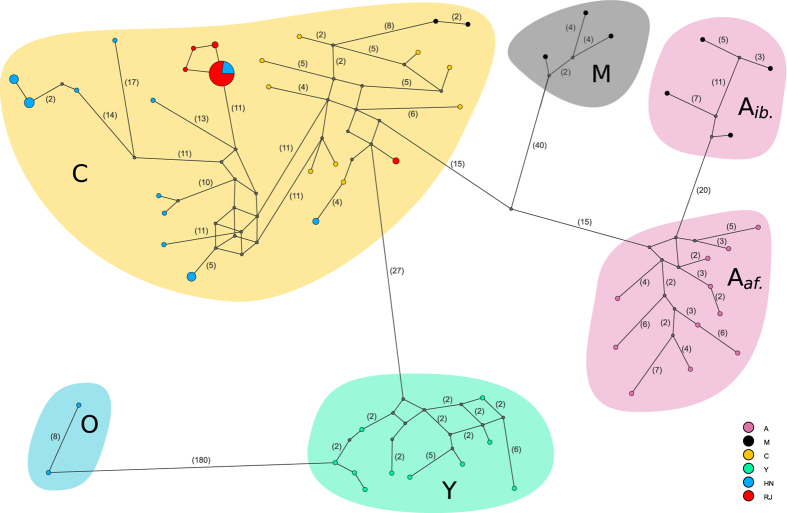
Haplotype network of whole mitochondrial genome. Median-joining network of whole mitochondrial sequences recovered from whole-genome sequence data. Haplotypes are coloured according to sample origin (A, M, C, Y, HN, RJ) and scaled according to the number of supporting sequences. Vertex labels indicate the number of segregating sites between haplotypes/nodes, and where missing are supported by one segregating site. Haplotypes were broadly grouped into their respective lineages (A, M, C, O, Y) based on these results, sampling origin, and Sanger sequence evidence in the case of the O lineage. Haplotypes grouped under lineage A are sub-grouped into A_*ib.*_ comprising bees from Iberia, and A_*af.*_ comprising bees from Africa.

Mitochondrial sequences extracted from the whole-genome alignments were used to construct an unrooted phylogenetic tree (Supplementary Fig. SF19) and haplotype network (Fig. 2). These results broadly support the ADMIXTURE results with the exception of two HN bees (HN9, HN19) falling in the O lineage and two M lineage bees (SRR957059, SRR957060) in the C lineage. To add further uncertainty, the two misplaced HN bees have the highest degree of admixture from the A lineage in the ADMIXTURE results at *K* = 5 (Fig. 1a). The phylogenetic tree indicates four M lineage bees (SRR957061, SRR957062, SRR957063, SRR957064) to form an intermediate cluster between the A lineage and remaining M lineage bees. These four bees were sampled from Spain^7^ where a well-defined northeastern-southwestern cline of M to A mitotypes has previously been demonstrated^25^, and which accounts for the branching in the tree (Supplementary Fig. SF19).

**Figure 3:**
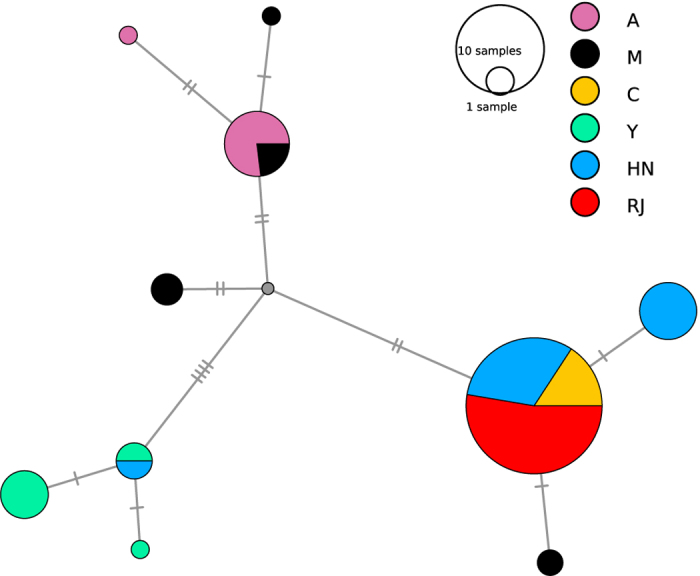
Haplotype network of mitochondrial tRNA^Leu^-cox2 intergenic sequence. Median-joining network of mitochondrial tRNA^Leu^-cox2 intergenic sequence recovered from whole-genome sequence data (see Methods). Haplotypes are coloured according to sample origin (A, M, C, Y, HN, RJ) and scaled according to the number of supporting sequences. Dashes on vertex labels indicate the number of segregating sites between haplotypes.

To assess the reliability of the method, due to the reference genome being derived from the C lineage, a number of drones (HN n = 5; RJ n = 9) were Sanger sequenced for the mitochondrial tRNA^Leu^-cox2 intergenic region. This highly variable region has been extensively used to characterise mtDNA haplotypes (mitotypes) of bees from different lineages and populations. Multiple alignment of these sequences, together with 68 references downloaded from GenBank representing each of the major lineages, supports the clustering of HN9 and HN19 with bees possessing an O lineage mitotype (Supplementary Fig. SF20). A method was also developed to retrieve the tRNA^Leu^-cox2 sequence from the whole-genome sequence data, so as to reconstruct the mitotype by referencing 226 sequences downloaded from GenBank. The efficacy of the approach was validated against the mitotype sequences of the same bees that were Sanger sequenced for the region (Supplementary Fig. SF21). Median-joining haplotype networks (Fig. 3; Supplementary Fig. SF22) arising from the subsequent alignment of these sequences also supports the earlier analyses.

### Heterozygous SNPs as potential copy number variants (CNVs)

Heterozygous SNPs (hetSNPs) distributed in clusters in haploid data can indicate CNVs^4^,^26^, and so the distribution and frequency of 1.2 M distinct hetSNPs identified across all drones was analysed. Several criteria were applied whilst processing the hetSNPs on a drone-by-drone basis. Specifically, hetSNPs located within 2 kb of another hetSNP were retained, resulting in a mean of 7 k hetSNPs per drone and a total of 71,951 distinct hetSNPs across all drones, of which 17 were common to 95% of individuals (Supplementary Fig. SF23). Post-processing, 814 clusters of hetSNPs were identified (Supplementary Fig. SF24), of which 95% were present in ≤15 drones. The 5% of intervals that were present in >15 drones (Supplementary Table ST8) were considered less likely to be sequencing or alignment artefacts, and analysed further. Of these 41 intervals, 13 overlap with previously identified crossover or gene conversion events^26^ and includes 1 of 3 intervals present in 90% of drones, indicating possible issues with the reference genome assembly. A summary of genes within 2 kb of each interval is provided in the Supplementary material (Supplementary Table ST9).

Although the majority (65%) of genes within 2 kb of these clusters lack annotation for gene ontology (GO) terms, a small number with GO terms are of potential interest. One cluster (13:9.56 Mb), which shares a similar frequency in both populations, hosts *CYP6AS5* (GB49890) and is proximal to several other cytochrome P450 genes. Chi-squared tests of the hetSNP interval frequencies in each population indicates several to have significant population-specific bias in frequency. For instance, 1:2.48–2.49 Mb was detected in 77% of RJ bees and 31% of HN bees (*P* = 2.8^−4^), whilst 15:0.27–0.28 Mb was detected in 39% of RJ bees and 79% of HN bees (*P* = 1.68^−3^). Another cluster (15:1.48 Mb, *P* = 4.1^−2^), present at higher frequency in HN bees, is proximal to at least 5 genes encoding odorant binding proteins. And whilst not significant, one cluster (10:4.67–4.68 Mb, *P* = 1.16^−1^) present at higher frequency in RJ bees spans the final exon of a gene (GB43369) annotated with GO terms linked to catalytic and hydrolase activity, specifically serine-type endopeptidase activity.

### Signatures of selection

**Figure 4:**
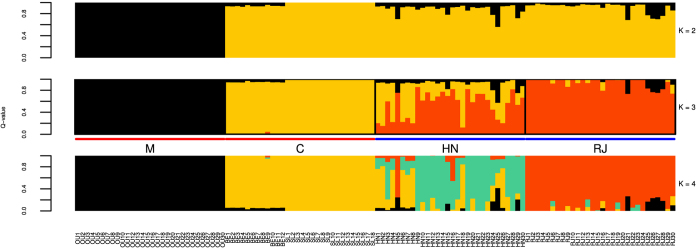
Population structure of HN and RJ drones to drones from lineages M and C. Lineage M is represented by drones sampled from a population in Ouessant (OU). Lineage C is represented by drones sampled from Germany (BE) and Slovenia (SL). The optimal K (3) is indicated by the additional population annotation.

HapFLK, a method estimating differences in haplotype frequencies between populations whilst accounting for their hierarchical structure, was employed to investigate the effects of selection on the HN and RJ populations. For optimum results, hapFLK requires at least 3 populations in addition to an outgroup. Additional drones were therefore included representing bees from lineage M, sampled from the Island of Ouessant (OU, n = 30), to serve as an outgroup for rooting of trees in hapFLK. Drones were also included from pure populations representative of lineage C, sampled from Berlin (BE, n = 12) and Slovenia (SL, n = 18), to provide a third population to contrast the HN and RJ populations. To check the suitability of the outgroup and lineage C samples, unsupervised ADMIXTURE analyses were performed on the populations (OU, BE, SL, HN, RJ) for K 1 to 6, whose CV error rate indicated K = 3 to be optimal (Fig. 4). At K = 2 the M lineage is differentiated from the C lineage and its derived populations (HN and RJ), whilst at K = 3 a new background dominates the RJ population. At K = 4 a further background emerges in the HN population, and the overall ancestry proportions assigned to each individual in the HN and RJ populations are consistent with the ADXMITURE analyses presented in the manuscript at K = 6 using reference populations from Harpur *et al*.^7^ (r ≥ 0.98). The results indicate the drones from the M and C lineages represent two homogenous populations, whereas the HN and RJ drones are shown to derive from the C lineage (K = 2) with the HN population exhibiting the greatest degree of admixture.

**Figure 5:**
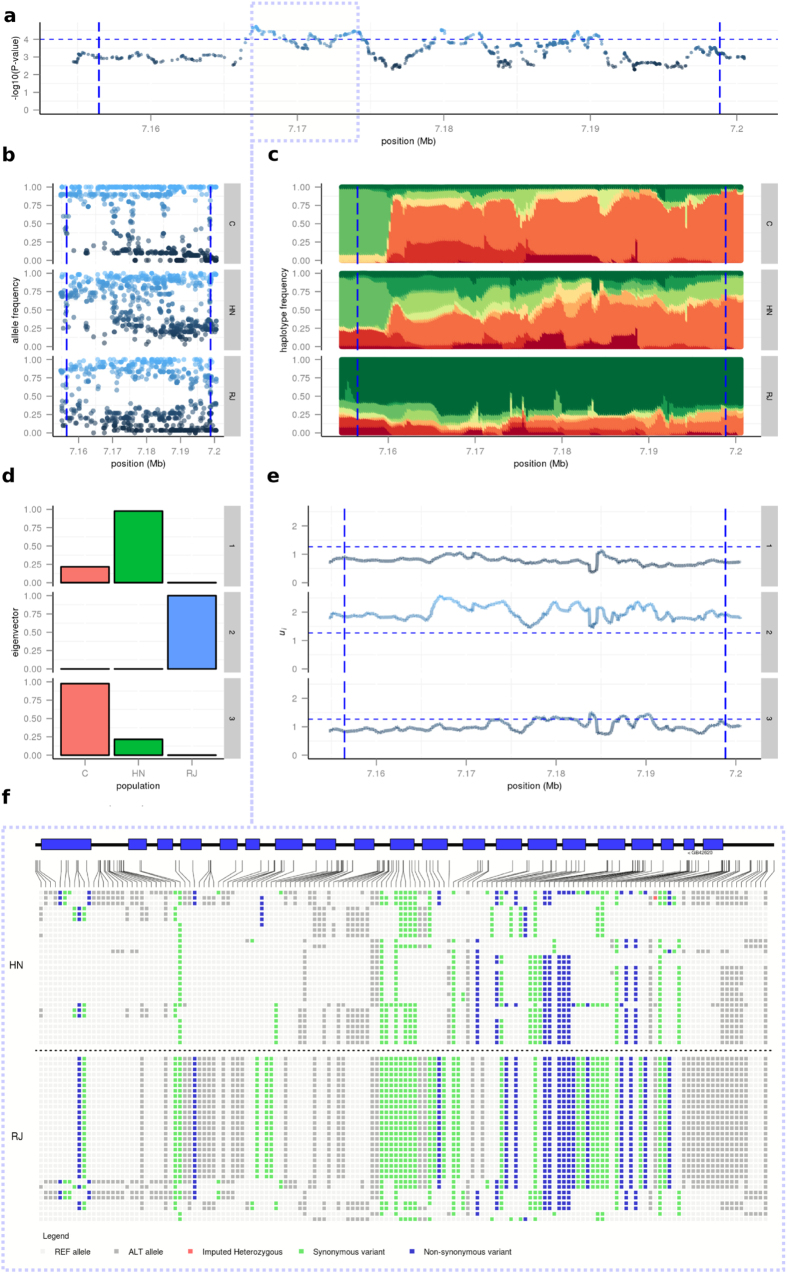
Example region (9:7.15–7.2 Mb) under putative selection in RJ population. (**a**) Plot of hapFLK values, dashed horizontal line indicates a 10^−4^ significance threshold. (**b**) SNP allele frequencies for each population (C, HN, RJ); the reference allele used for the SNP frequency representation is arbitrary; colour gradient denotes allele frequency. (**c**) HapFLK haplotype cluster frequencies for each population (C, HN, RJ); colours denote individual haplotypes. (**d**) Bar plot of *Q*_*i,j*_ values following spectral decomposition of kinship matrix, see main text for further details. (**e**) Plot of *u*_*i*_ values following spectral decomposition of kinship matrix, see main text for further details. (**f**) Haplotype schematic illustrating genotypes per individual in HN and RJ populations for a subset of the identified region. Vertical dashed lines in plots (**a**–**c**,**f**) illustrate the boundaries of a region identified as significant in the hapFLK test.

Chromosome-wide plots following the original FLK statistic and the hapFLK analysis are provided in the Supplementary material (Supplementary Figs SF25–SF40). Where significant signals are identified this is suggestive that one population diverges from the others. To identify the population under selection spectral decomposition of the kinship matrix is performed, as described by Fariello *et al*.^27^. In summary, can be decomposed as *Q*’*DQ*, were *Q* is an orthogonal matrix and *D* is a diagonal matrix with the eigenvalues of on the diagonal. Denoting the vector of allele frequency variations from the ancestral population as 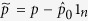 the *T*_*FLK*_ statistic^28^ can be re-written^27^ as 
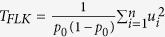
 where 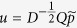 and thus *u*_*i*_ is the contribution to the test of the *i*^th^ eigenvector. Plotting the *u*_*i*_’s independently indicates which eigenvector(s) most contribute to a corresponding peak in *T*_*FLK*_ values. *Q*_*i,j*_ provides the loading of population *j* in eigenvector *i*, such that large absolute values of *Q*_*i,j*_ pinpoint the population represented by eigenvector *i*. An example of this is presented in Fig. 5, whereby a region (9:7.15–7.2 Mb) identified as significant (Fig. 5a) is examined in each population to reveal allele (Fig. 5b) and haplotype (Fig. 5c) frequencies. The loading of each population (Fig. 5d) for a given eigenvector (Fig. 5e) enables the delineation of the population under selection for the corresponding peak in hapFLK values (Fig. 5a). In this instance, the peak corresponds with positive values in the 2^nd^ eigenvector (Fig. 5e), which relates to the RJ population (Fig. 5d). Visualising the genotypes for each individual in the HN and RJ populations within a subset of this region (Fig. 5f) further reveals the extent of selection.

The hapFLK results were considered significant at *P* ≤ 10^−4^, and regions defined by clustering *P* values within 2 kb of one-another and extending to capture 10 kb flanking (Supplementary Table ST10). Following spectral decomposition, a population was considered under selection if the measure of selection for a given region was at least twice that of the other populations, resulting in the detection of one region under selection in lineage C, and 9 and 44 in the HN and RJ populations, respectively (Supplementary Table ST10). For the HN and RJ populations, the nearest genes on forward and reverse strands within 20 kb of the most significant *P* value per region were identified (Supplementary Tables ST11–12). The single region identified to be under selection in lineage C comprises two genes for which no annotation is available.

Several of the genes identified in the HN population (Supplementary Table ST11) are orthologus to genes in *Drosophila* with functions linked to the muscular and nervous systems. Specifically, these include: *sls* (GB47977), demonstrated to play a key role in the development and function of striated muscles and muscle tendons; *dlp* (GB42671), associated with neuromuscular and compound eye development, and the *Wnt* and smoothed signalling pathways; *Rim* (GB54272), involved in regulation of synaptic plasticity and neuromuscular synaptic transmission; and *kuz* (GB49985), involved in the notch and roundabout signalling pathways, lymph gland, ventral cord and muscle tissue development. Collectively this suggests a selective pressure acting on genes involved in the muscular and nervous systems, however this is not evident in a GO term analysis (g:GOst; Supplementary Table ST13).

Of the genes identified in the RJ population, several encode proteins that have been previously highlighted in a proteomics study^29^ of differentially expressed proteins in nurses and foragers. Given that nurse bees are responsible for feeding larvae and producing royal jelly, genes identified in our RJ population that overlap with those from studies concerning nurse bees are of particular interest. These include sequences predicted to be similar to: the HECT domain, present in *Ube3a* (GB51221); the PACS-2 domain, similar to the PACS-1 domain in *KrT95D* (GB48889); *mRpL38* (GB45886); *Stretchin-Mick* (GB47949); *Cog7* (GB52588); *Rpn2* (GB51372); *jagn* (GB49305); *Cht7* (GB48474); and CG16791 (GB54308). Of these genes, *Stretchin-Mick* is the most significant in our results, and is involved in the regulation of the glucose metabolic process. A high rate of synthesis of vitellogenin is characteristic of nurse bees, and vitellogenin was detected only in nurse bees in the proteomics study. Although the vitellogenin gene (*Vg*) was not identified in our results, one of the genes identified, *jagn*, plays a role in vitellogenesis through oocyte endoplasmic reticulum reorganisation^30^. Subjecting the genes to analysis with g:GOst revealed only five genes (GB47950, GB51190, GB48482, GB43293, GB49411) to indicate enrichment in biological processes (Supplementary Table ST14), most notably glycine and carboxlyic acid transport, and acetate ester and acetylcholine metabolic processes.

More than a third of genes identified in the RJ population lacked orthologous genes in *Drosophila* impairing the effectiveness of the g:GOst analysis. To compensate, an analysis of the information content (IC) of GO terms of the *A. mellifera* genes was conducted using the IT-GOM tool of DaGO-Fun^31^. As with the initial GO term analysis in the HN population, there is no apparent link to the muscular and nervous systems as might have been expected based on the annotations of the orthologs identified. However, the high-scoring DNA-directed RNA polymerase terms (Supplementary Table ST15), and the presence of ribonucleoprotein complex and spliceosomal complex disassembly in the g:GOst analysis (Supplementary Table ST13), are supported by their identification in an earlier study on neurogenomic signatures of spatiotemporal memories in time-trained forager honey bees^32^. That study found that genes annotated for these GO terms were upregulated in inactive bees, and the authors speculate that inactive foragers enter a sleep-like state, during which time old proteins might be purged and new proteins synthesised.

The IC of GO terms from genes identified in the RJ population is dominated by annotations linked to RNA, which includes the three highest topology-based IC scores (Supplementary Table ST16), consistent with the identification of RNA modification and tRNA methyltransferase complex terms in the g:GOst analysis (Supplementary Table ST14). Other high-frequency annotations include those linked to ‘ubiquitin’ and ‘serine’. Ubiquitin is a small regulatory protein which, as the name implies, is found in almost all eukaryote tissues and functions in a variety of protein modifications. The final step of ubiquination, an enzymatic post-transaltional modification, is catalyzed by E3 ubiquitin ligases, which possess one of two domain – one being the homologous to the E6-AP carboxyl terminus (HECT) domain. As mentioned previously, one of the genes identified in the RJ population, *Ube3a* (GB51221), possess a HECT domain, which in a proteomics study was detected only in the brain of nurse bees^29^; a further gene (GB50392), orthologous to *Usp38*, has GO terms linked to protein deubiquitiniation. Serine endopeptidases are ubiquitous enzymes and are responsible for co-ordinating a variety of physiological functions. Two genes have annotations linked to serine (GB43369, serine-type peptidase activity; GB45876, protein serine/threonine kinase activity), of which one is orthologs to the ribosomal protein *S6k* (GB45876). The activation of *S6k* by royalactin, a royal jelly protein, has been found to play a role in the increase of body size in queens^33^. The identification of genes encoding ribosomal proteins (*S6k, mRpL38*) is consistent with the findings of several proteomics studies, in which differential expression of ribosomal proteins in nurse bees have been observed^29^,^34^,^35^.

Within the 71 genes potentially under selection in the HN and RJ populations, 433 non-synonymous variants were identified (Supplementary Table ST17). Functional importance of these variants, analysed with PROVEAN, indicates 30 predicted to be deleterious, distributed across 17 genes. None of the predicted deleterious variants were fixed in either population, and only one was in the 90^th^ percentile of difference in allele frequency (ΔAF = 0.5) between HN and RJ populations for all non-synonymous variants, indicating none to be strongly divergent. The single variant in the 90^th^ percentile of ΔAF is located in the tenectin gene, *tnc* (GB53632), which is annotated with GO terms linked to morphogenesis of wing disc, male genitalia, epithelial tube and embryonic hindgut. Significant differential expression of *tnc* has been observed in hind leg development between queen and worker bees^36^, however there appear to be no other studies on the gene in the honeybee to provide any further insights.

## Discussion

This study demonstrates the advantages of sequencing drones for large-scale studies of population genomics in the honeybee. An assessment of the optimal economic sequencing depth indicated that DP ≥ 7 resulted in little gain in the fraction of genome callable (GX), and to obtain GX ≥ 0.8 requires sequencing at disproportionately greater DP for marginal gains. It is therefore more biologically and computationally informative to sequence two drones at 7 X rather than one worker at 14 X. This is particularly pertinent for social insects, where the benefits of capturing the broad genomic diversity of a colony outweigh those of a few individuals captured at higher resolution. A further consideration of concerning the use of drones is that only the queen’s contribution to the colony is retrieved. This could be considered disadvantageous when attempting to characterise the genetic diversity of a population. Conversely, it could be considered advantageous when assessing commercial populations and conservation programmes where many of the decision-making processes relate to queens.

The higher level of π observed in the A lineage together with the greater number of private SNPs identified is consistent with observations in previous studies^4^,^37^. The reduced π in RJ compared to HN bees is further evidenced in the ADMIXTURE results, in which the population forms a distinct background at *K* ≥ 5. The low *F*_*ST*_ (0.139) between the C lineage and RJ bees suggests this background most likely reflects sub-structuring within the C lineage. This is further evident in the phylogenetic trees and is likely to be a consequence of closed-population management practices of the RJ apiculturists. In summary, the data suggests HN and RJ bees originate from the C lineage, from which the majority of domestic strains are derived^5^, with a fraction of admixture with the M lineage being evident in some individuals.

Analysis of the mitochondrial genome revealed the unexpected placement of four bees. Two of these were HN drones (HN9, HN19), which Sanger sequencing confirmed to possess O lineage mitotypes, whilst the other two bees were from the M lineage in the reference dataset. ADMIXTURE analyses indicated the HN drones to originate predominantly from the C lineage, with minor admixture from the M lineage. In the ADXMITURE results, the two HN drones found to possess O lineage mitotypes revealed a Y lineage ancestry fraction of 0.11–0.12 at *K* = 4, switching at *K* = 5 to an A lineage ancestry fraction of 0.28–0.3. Future availability of whole-genome sequence data from O lineage bees will provide an opportunity to address the discordance in these results. Concerning the assignment of two M lineage bees (SRR957059, SRR957060) to the C lineage, these two bees were sampled from Sucha Rzeczka in Poland whilst the remaining M lineage bees sampled from within Poland originated from Zalewo and Rudawka. It is therefore possible that the Sucha Rzeczka bees are not as pure as the other M lineage bees from Poland. With the exception of these latter two bees, the remaining M lineage bees formed two groups – one of which was close to the A lineage (Fig. 2). M lineage bees in the group proximal to the A lineage were sampled from Spain, where a well-defined northeast to southwest clinal pattern of M to A lineage ancestry has been demonstrated^25^, accounting for the bifurcation of M lineage bees in our results.

The Amel4.5 reference genome is derived from *A. m. ligustica*, and as a consequence possesses a C1 mitotype for the tRNA^Leu^-cox2 intergenic sequence widely used in classical mitotype studies. Different mitotypes are characterised by a number of point mutations and indels in the ‘P’ element, and the number of duplications of the ‘Q’ element^38^. To compare whole-genome sequence data for the region with Sanger sequence mitotypes requires a different strategy than simply mapping reads to the reference genome. The method developed here to reconstruct mitotypes from whole-genome sequence data was validated using a number of individuals for which Sanger sequences were also available. PopArt^39^ was used to generate the haplotype network, however this masks sites with more than 5% missing data, resulting in the masking of the P element due to its absence in C-lineage mitotypes. Consequently, the network is based on only 16 segregating sites. Similarly, the absence of duplications of the Q element in some sequences would result in these sites being masked also. These issues can be mitigated by re-coding sequences. For instance a recent study coded Q elements as present or absent to ensure they were retained in the analysis^25^. A network is provided in the supplement (Supplementary Fig. SF22), in which sequences were reduced to 3 nucleotides by re-coding ‘C’ as ‘G’, and then re-coding gaps as ‘C’. The results indicate that despite the complex genetic architecture of *A. mellifera* mitotypes, comprising point mutations, indels and duplications, it remains possible to retrieve mitotype sequences from whole-genome sequence data. This facilitates their analysis in context with the extensive mitotype sequence data available in GenBank, providing further insight into the mitochondrial history of the sequenced specimen. However, the discordant population assignment of two HN bees and two M lineage bees when comparing mitochondrial (Supplementary Fig. SF19) and autosomal (Fig. 1) analyses highlights the risks inherent when studying maternally inherited loci in isolation.

Management and selection of honeybees in France is far from having reached the degree of sophistication seen for other species such as cattle or poultry. The commercial aim for the HN population is to develop colonies possessing desirable characteristics for honey production, which can include for instance productivity, docility, and resistance to disease. Selection is thus not very strong towards any one characteristic, and broadly corresponds to the global objective since the domestication of the honeybee. By contrast the RJ population is managed solely for royal jelly production, and undergoes an annual evaluation of productivity, the results of which determine which colonies are selected to produce the next generation. The population was established within the last 10 years, with many of the queens having been imported from China where selection for royal jelly was established in the 1950s using *A. m. ligustica* colonies originally introduced for honey production. Since then royal jelly production per colony in China has increased from ~0.2–0.3 kg per year to more than 10 kg per year^40^. Other differences observed between the unimproved and high royal jelly production lines include: more numerous and larger hypopharyngeal gland acini in RJ bees; positive correlation between hypopharyngeal gland length and royal jelly production; increased hypopharyngeal gland secretory activity and duration; and greater quantities of mitochondria, rough endoplasmic reticulum, secretion granules and secretion masses in hypophayngeal gland cells^40^. The closed-population management practices of the RJ apiculturists following the historical bottlenecks likely to have occurred following the initial import and selection of bees to China, likely contributes to the differentiation of the RJ population from the other C lineage bees in the ADMIXTURE results and phylogenetic trees (Fig. 1).

An advantage of sequencing drones, highlighted in this study, is the capacity to explore clusters of hetSNPs as putative CNVs. Where these are present at high frequency they detract from the likelihood of being a sequencing or mapping error. Several such clusters were identified, with population-specific bias in frequency, and further research may associate such biases in frequency with selection. Indeed, the GO term annotations of nearby genes in some of these clusters, and the presence of clusters near to the signatures of selection detected by hapFLK, hints at such a possibility. For instance, several encode odorant binding proteins, and one gene identified is involved in serine-type endopeptidase activity - a serine protease and a carboxypeptidase have been inferred to be the major enzymes involved in the hydrolysis of royal jelly proteins^41^. However, further investigation is required to experimentally validate these findings.

Selection results in an increase in linkage disequilibrium (LD) within the affected region of the genome, leading to the emergence of different haplotypes in populations subject to different selection pressures. The size of haplotype blocks is influenced by the strength of selection and historical recombination. Recent selection is typically characterised by large haplotype blocks, whilst the opposite is often true of historical selection as the increasing number of recombination events over the generations leads to a reduction in block size. The influence of haplotype blocks on genome structure has enabled association studies to be performed in several domestic species using small sample sizes, without the need for mapping resource populations^42^,^43^. The most widely used statistic to test localised genetic differentiation between populations is *F*_*ST*_. Typically this involves detecting outliers in the empirical distribution of statistics computed genome-wide, a major caveat of which is that it assumes populations have the same effective size and are derived independently from the same ancestral population^27^. If this assumption is false then the results can suffer from bias and false positives, not dissimilar to the effect of cryptic structure in the data^44^. One proposed solution, the FLK statistic, is to employ an extension of the Lewontin and Krakhauer (LK) test which incorporates a co-ancestry matrix (*F*) between populations, under pure drift with no migration^28^. More recently, hapFLK^27^ builds on the original FLK statistic to also account for haplotype structure using a multipoint LD model, and has been demonstrated in sheep^27^,^45^ and chicken^46^ to decrease false detection rate. This statistic is particularly suited to the analysis of drones given their haploid nature, and is recommended as a first step towards identifying genomic regions of interest for future characterisation^47^. Several of the regions identified in the RJ population in this study contain genes encoding proteins that have been found to be differentially expressed in proteomic studies of forager and nurse bees. These include genes linked to ubiquination, serine-type peptidase activity, ribosomal proteins, vitellogenin, and glucose metabolism, providing a number of avenues for further investigation to better characterise the genetics of this commercially important trait.

## Methods

### Sampling and sequencing

The royal jelly (RJ) population was sampled from 3 apiaries which are part of the Groupement des Producteurs de Gelée Royale (GPGR) selection programme. These bees are selected solely for royal jelly production using specific beekeeping guidelines described in^48^. Within this programme, each year around 200 colonies undergo a royal jelly production trait evaluation (quantity of royal jelly produced), and around 20 are selected to produce the next generation. This selection programme started in France around 2007 with queens used by royal jelly producers, many of whom imported from China where selection for royal jelly is well-established. The honey (HN) population was sampled at a commercial queen breeder apiary in Tarn (France), for which the selection objectives mirror those typically applied by commercial honey producers – according to quantity of honey produced. The other C lineage populations were sampled from the Kmetijski Inštitut Slovenije (Agricultural Institute of Slovenia) and from the Institute for Bee Research Hohen Neuendorf in Germany. The M lineage population was sampled from Ouessant, an island in Brittany (France), where it has remained in isolation for around 30 years. One drone per colony was sampled at the pupae/nymph stage or at the larval stage if no male pupae/nymphs were available at the time of sampling. Further details on DNA extraction and treatment are provided in the Supplementary material. Sequencing was performed on Illumina HiSeq 2000 and 2500 platforms, with 20 samples per lane, following the manufacturer’s protocols for library preparations (2 × 100 bp, or 2 × 125 bp). Sequencing reads of 39 *A. mellifera* workers were downloaded from the European nucleotide archive (ENA; study accession: PRJNA216922) to act as a reference dataset^7^.

### Mapping and variant detection

Sequencing reads were mapped to Amel4.5 using BWA-MEM^v0.7.9a;^^49^, duplicates marked with Picard (v1.88; http://picard.sourceforge.net), and local realignment and base quality score recalibration (BQSR) performed using GATK^v3.3−0;^^50^. SNPs were called in each drone independently and consolidated into a single set of master sites, from which all individuals were genotyped (see Supplementary Material). A filtering step in Plink (v1.9; https://www.cog-genomics.org/plink2) retained SNPs on chromosomes 1 to 16 with minor allele frequency (MAF) ≥ 0.05 and genotyping call rate ≥0.9. Missing genotypes were imputed using BEAGLE^v4;^^51^. Minor alterations were implemented to the pipeline to facilitate analysis of the diploid sequence data downloaded from the ENA, specifically this required the retention of heterozygous SNPs. Further details are provided in the Supplementary material.

To quantify variants by consequence, the VCF files for drones from the populations selected for honey (HN) production and those selected for royal jelly (RJ) were merged to create a single VCF file for each population. SNPs with a non-reference allele count (AC) > 3 (equivalent to 10% of the population) were analysed using the Perl version (v78) of Ensembl’s Variant Effect Predictor^52^ and the Amel4.5 variation database (apis_mellifera_core_25_78_45). Where reported, the effects of amino acid substitutions on protein function were assessed using the default parameters of PROVEAN^53^.

### Population structure and relationships

For each evolutionary lineage (A, C, M, Y) and population (HN, RJ), nucleotide diversity (π) was calculated in 5 kb windows with 1 kb step size using VCFtools^v0.1.12a;^^54^, and averaged to generate a genome-wide π for each lineage and population. To assess population structure using ADMIXTURE^v1.23;^^55^, diploid individuals were first constructed from the drones by combining all SNPs using both alleles from randomly selected pairs of drones. The diploid drone and reference datasets were merged and filtered to retain SNPs on chromosomes 1 to 16 with minor allele frequency (MAF) ≥ 0.05 and genotyping call rate ≥0.9. ADMIXTURE was run unsupervised assuming 1 ≤ *K* ≤ *6* with 10 bootstrap replicates. The optimal *K* was inferred from the minimum observed cross-validation error, and Q estimates plotted in R. The SNP data used for the ADXMITURE analysis was imported into R via the GenABEL package^56^, and an unrooted phylogenetic tree constructed using the complete linkage agglomeration method and plotted using the APE package^57^. For comparison, this analysis was also performed using a combined dataset of reference individuals and haploid drones.

### Mitochondrial analyses

Consensus sequences were recovered for each sample for the mitochondrial genome from BAM files using SAMTOOLS and BCFTOOLS, and aligned with ClustalW^58^. The alignment was analysed with jmodeltest2^59^,^60^ to identify the best-fit model of nucleotide substitution, including models with a proportion of invariable sites and unequal base frequencies. The results from the GTR+I+G model identified by the Akaike Information Criterion (AIC) were used to configure MrBayes^61^: (a, c, g, t) = (0.43, 0.10, 0.05, 0.42), R(a, b, c, d, e, f) = (2.140, 27.536, 2.3000, 0.940, 46.066, 1.000), which was run for 2 M generations and the results plotted using FigTree v1.4.2 (http://tree.bio.ed.ac.uk/software/figtree). Several drones (HN n = 5; RJ n = 9; Supplementary Figs SF20–SF21) were mitotyped by sequencing the mitochondrial tRNA^Leu^-cox2 intergenic region, amplified using primers E2 and H2^38^. Sanger sequences were processed using FinchTV (http://www.geospiza.com) and aligned with MAFFT^62^. MrBayes was used to generate a consensus tree which was subsequently plotted using FigTree. A haplotype median-joining network was generated using PopArt.

To analyse the whole-genome sequence data with reference to historical mitotypes generated by traditional Sanger sequencing of the tRNA^Leu^-cox2 region, reference sequences for 226 mitotypes were downloaded from GenBank. Sequences were trimmed to start from the 3′ end of tRNA^Leu^ ‘CTTTTATTAAA’ and terminate at the 5′ end of COX2 ‘ATTTCCACA’. For each sample, reads spanning the tRNA^Leu^-cox2 genomic region (NC_001566.1:3355-4295) were recovered from the BAM file and converted to FASTQ using SAMtools. These reads were then mapped to each reference mitotype and the error rate (*e* = PF_HQ_ERROR_RATE + PF_INDEL_RATE) and alignment quality (*q* = PF_HQ_ALIGNED_Q20_BASES/PF_ALIGNED_BASES) calculated with Picard. Where *e* > 0 & *q* > 0.5 the accession number, sequence length and *e* to 6 decimal places were recorded. Requiring *e* > 0 is necessary to avoid accepting a perfect alignment to a very short mitotype where for example an alignment of 99% identity is present for a longer mitotype. Requiring *q* > 0.5 filters out alignments in which the majority of base quality scores are poor. The alignment with the lowest error rate, and longest sequence length in the event of identical mismatch rates for multiple sequences, was considered the best match. From this alignment, SNPs were identified using SAMTOOLS mpileup and a consensus FASTA generated using BCFTOOLS. The resulting sequences were aligned using ClustalW and a haplotype network constructed using PopArt.

### Heterozygous SNPs

SNPs were re-called in haploid individuals and heterozygous SNPs (hetSNPs) retained for analysis. Sites were filtered to retain those on chromosomes 1 to 16 with depth of coverage (DP) ≥ 9 across all individuals. For each individual, hetSNPs that were >2 kb apart were removed and the remaining sites together with DP recorded in BED format. Sites were then processed in R on a drone-by-drone basis to retain clusters containing ≥3 hetSNPs that spanned at least 2 kb and had a mean DP ≥ 3 times the average for the drone. R was used to identify clusters overlapping with crossover and gene conversion events identified by Liu *et al*.^26^, conduct chi-squared tests, and identify *A. mellifera* genes within 2 kb of the clusters.

### Signatures of selection

Tests for signatures of selection were conducted using hapFLK^27^. Additional drones were included to represent “progenitor” bees from lineage M, sampled from Ouessant (OU, n = 30), to act as an outgroup for rooting of trees in hapFLK. Additional drones were also included from pure populations representative of lineage C, sampled from Berlin (BE, n = 12) and Slovenia (SL, n = 18), to provide a contrast to the selected HN and RJ populations. The additional drones were sampled, sequenced and processed using the same procedure outlined above, and subsequently genotyped for the SNPs identified in the HN and RJ populations. The hapFLK analysis was first conducted on the genome-wide dataset of SNPs to generate a kinship matrix from pairwise Reynolds’ distances between populations using lineage M as the outgroup. The analysis was then performed for each autosome, taking into account the kinship matrix, assuming 10 haplotype clusters, and run for 20 expectation maximization (EM) iterations. *P*-values were calculated from the hapFLK chi-squared density, and loci considered significant with hapFLK *P*-values of the order 10^−4^. Significant regions were defined by the minimum and maximum positions of significant *P*-values within 2 kb of one-another, and further extended to capture 10 kb flanking. For each region, the spectral decomposition method described by Fariello *et al*.^27^ was employed to pinpoint the population (*j*) under selection. Within each region *u*_*i*_ values in the 99^th^ percentile were summed for each eigenvector (*i*) and divided by the number of SNPs in the region to provide a measure of selection. The population under selection was identified from the eigenvector and loadings of the *Q*_*i,j*_ matrix (for further details refer to Supplementary methods of Fareillo *et al*.^27^).

### Gene Ontology analysis

*Drosophila melanogaster* orthologs (Supplementary Table ST17) were identified using the g:ORth tool and submitted for profiling using the g:GOSt tool of g:Profiler^63^. *D. melanogaster* orthologs were chosen for analysis over *A. mellifera* genes due to the more comprehensive reference databases available for the former. Due to the absence of orthologs for some of the genes identified, an analysis of GO term information content was conducted using the IT-GOM tool in DaGO-Fun^31^ on GO terms of *A. mellifera* genes identified in each population. Two approaches were applied, an annotation approach which included inferred electronic annotations, and the GO-universal topology approach.

## Additional Information

**Accession codes**: Sequencing reads for the samples supporting this study are available in the NCBI Sequence Read Archive repository under study Accession: SRP069814.

**How to cite this article**: Wragg, D. *et al*. Whole-genome resequencing of honeybee drones to detect genomic selection in a population managed for royal jelly. *Sci. Rep.* **6**, 27168; doi: 10.1038/srep27168 (2016).

## Supplementary Material

Supplementary Material

Supplementary Tables
