# Supplementary material for: Whole-genome resequencing of honeybee drones to detect genomic selection in a population managed for royal jelly
Source: Sci Rep. 2016 Jun 3;6:27168. doi: 10.1038/srep27168 (PMC4891733; doi:10.1038/srep27168)
Supplement: Supplementary Material [file srep27168-s1.pdf]

Whole-genome resequencing of honeybee drones to detect genomic selection in a population managed for royal jelly

David Wragg[1*], Maria Marti[1], Benjamin Basso[2], Jean-Pierre Bidanel[3], Emmanuelle Labarthe[1], Olivier Bouchez[1], Yves Le Conte[4], Alain Vignal[1]

Supplementary Methods and Figures

Supplementary Methods

Sampling

The honeybee population was sampled at a commercial queen breeder apiary in Tarn (France), and the royal jelly population from 3 apiaries which are part of the Groupement des Producteurs de Gelée Royale (GPGR) selection program. One drone per colony was sampled at the pupae/nymph stage or at the larval stage if no male pupae/nymphs were available at the time of sampling, and immediately placed in absolute ethanol for storage at -20°C. Total DNA was extracted from the thorax of the drones or from whole larvae cut into small pieces with a scalpel. The fragments were first incubated 3 hours at 56° in 1 mL of a solution containing 4 M urea, 10 mM Tris-HCl pH 8, 300 mM NaCl, 1% SDS, 10 mM EDTA and 0.25 mg proteinase K, after which 0.25 mg proteinase K was added for an incubation over-night at 37°C. Four hundred µL of a saturated NaCl solution was added to the incubation, which was then gently mixed and centrifuged for 30 minutes at 15000 g. The supernatant was treated for 5 minutes at room temperature with RNAse (Qiagen) and then centrifuged again, after which the DNA in the supernatant was precipitated with absolute ethanol and re-suspended in 100 µL TE 10/0.1. Pair-end sequencing was performed on Illumina HiSeq 2000 and HiSeq 2500 platforms, with 20 samples per lane, following the manufacturer's protocols for library reparations.

Mapping and variant detection

A bioinformatics pipeline was developed for processing the NGS data, in which sequencing reads were mapped to Amel4.5 using BWA-MEM [v0.7.9a; 1], duplicates marked with Picard (v1.88; http://picard.sourceforge.net), and local realignment and base quality score recalibration (BQSR) performed using GATK [v3.3-0; 2]. Each drone was processed with the pipeline independently, using SNPs called with GATK's UnifiedGenotyper as covariates for BQSR. Fraction of genome callable was calculated using GATK's CallableLoci tool, with a minimum DP ≥ 3 in haploids and minimum DP ≥ 6 in diploids, and with minimum DP for low mapping quality of 4 and 9 for haploids and diploids, respectively. Post-alignment, SNPs were called in each drone independently using three variant detection tools: UnifiedGenotyper, SAMtools mpileup [v1.1; 3] and Platypus [v0.5.2; 4]. The filtering criteria of all callers included a base quality (BQ) score ≥ 20 and MQ ≥ 30. In addition, UnifiedGenotyper calls were filtered for maximum number of alternate alleles = 2, genotype quality (GQ) ≥ 30, quality by depth (QD) ≥ 2, and Fisher strand (FS) ≤ 60. SAMtools mpileup also included a coefficient (C) for downgrading the MQ for reads containing an excessive number of mismatches = 50.

Variant call files (VCFs) containing homozygous SNPs from each tool were used as inputs for BAYSIC [5], which performs Bayesian latent class analysis to estimate false positive and false negative

error rates for each input. Variant sites exceeding a posterior probability threshold of 0.8 were retained. The sites identified across all individuals within a dataset were were then merged and filtered on depth of coverage (DP) to generate a list of sites for that dataset, with $9 \leq DP \leq 3\mu DP$ in which μ = mean. Each dataset was then merged to create a final set of master sites. In all individuals, variants were re-genotyped with the UnifiedGenotyper (BQ ≥ 20) at the master sites and any heterozygous variant calls set to missing in haploid individuals. The dataset was reduced to chromosomes 1 to 16 and subsequently filtered in Plink (v1.9; https://www.cog-genomics.org/plink2) to retain SNPs with minor allele frequency (MAF) ≥ 0.05 and genotyping call rate ≥ 0.9. Missing genotypes were then imputed using BEAGLE [v4; 6]. Minor alterations were implemented to the pipeline to facilitate analysis of the diploid sequence data downloaded from the ENA, specifically this required the retention of heterozygous SNPs.

Optimal economic sequencing depth regarding fraction of genome callable

To calculate the optimal target DP at which to sequence haploid drones, taking into account diminishing returns in the fraction of genome callable (GX) with increasing DP, two drones (H1 and H2) were sequenced at moderately high depths of coverage (DP = 16.9 ± 2.1). The alignment data was then sequentially down-sampled to approximate sequencing at depths of 10, 7, 5 and 3 X coverage. For each depth of coverage, the down-sampled BAM for each drone was merged, the read group information updated, the alignment indexed, and duplicates marked. The depth of coverage for the resulting diploid dataset was confirmed using GATK. Local polynomial regression fitting of DP against GX indicated that DP ≥ 7 X resulted in little gain of GX ≥ 0.8 (Supplementary Material SM1), and that to obtain higher GX requires sequencing at disproportionately greater DP for a marginal gain in information. It is therefore more biologically and computationally informative to sequence two haploid individuals at 7 X rather than one at 14 X. This is particularly pertinent for social insects, where the benefits of capturing the broad genomic diversity of a colony outweighs that of a few individuals captured at higher resolution.

Based on these results we decided to assess the suitability of drones for characterising population diversity and detecting signatures of selection, whilst optimising cost by targeting an optimal sequencing depth. Drones were sampled from two populations, one used for honey production (HN; n = 30) and the other selected for royal jelly (RJ; n = 30). Based on the results of the modelling, using the two previously sequenced drones, samples were sequenced at a target DP of 6 X. The resulting DP ranged from 3.49 to 13.36 (mean DP = 6.78) and GX between 0.5 and 0.83 (mean GX = 0.68), suggesting the RJ and HN sequencing runs to be less consistent than the down-sampled test data (Supplementary Table ST1). Analysis of the results per sequencing run (1 to 3; Supplementary

Material SM1) did not indicate an association between any specific sequencing run and a deviation from the model. To further investigate this genome coverage problem, we used preliminary sequence data of drones from two other populations not used further in the present study: Ile d'Ouessant (OUE; n = 20; run 4) and Corse (AOC; n = 20; run 6) at DP ranging from 3.8 to 15.6 X. The highly correlated fit of this data to the model (OUE $r = 0.97$, AOC $r = 0.91$), and across both populations (OUE ~ AOC $r = 0.99$), suggests some initial teething problems either with DNA isolation, library preparation or sequencing in the HN and RJ populations (Supplementary Material SM1).

Ploidy comparison with regards to variant detection

To evaluate the effect of ploidy and DP on the power and accuracy of variant detection, SNPs were called independently for the two haploid drones (H1 and H2) down-sampled at various sequencing depths, and from a diploid individual generated *in silico* by merging the alignment files of the two drones. This allowed comparing the total number of SNPs detected using two haploid individuals effectively sequenced each at DP of 15, 10, 7, 5 and 3 X, with the corresponding diploid individuals containing exactly the same reads, effectively sequenced at DP of 30, 20, 14, 10 and 6 X. Haploid and diploid SNP detection sensitivity and specificity could thus be estimated. As expected, a strong linear correlation ($r = 0.999$) was observed between the number of SNPs detected for each ploidy with increasing DP (Supplementary Material SM2). The total number of SNP detected in the haploid data (1.76 M ± 254 K) was higher than in the diploid data (1.61 M ± 290 K). Moreover, this number remains higher even if all heterozygous SNPs (68K ± 25 K) are removed from the haploid data, as they might be considered potential sequencing errors, sample contamination or other artefacts. The number of private SNPs identified, those exclusive to either the haploid or diploid dataset, were 179 K ± 49 K and private = 29 ± 5 K, respectively.

A small proportion of heterozygous SNPs (~ 3%) is consistently detected in each haploid individual (Supplementary Material SM2) and although intuitively unexpected in haploid data, and possible artefacts, there is evidence to indicate that such heterozygous SNPs might be linked to copy number variation [35]. A significant advantage of sequencing haploids over diploids therefore, is that such events can be investigated. If we were to assume that all of the SNPs identified in the haploid data are true, then it can be inferred that the private SNPs (~ 29 K) identified in the diploid data are likely to be false positives, suggesting a SNP false discovery rate ≈ 0.015 to 0.033 in diploids.

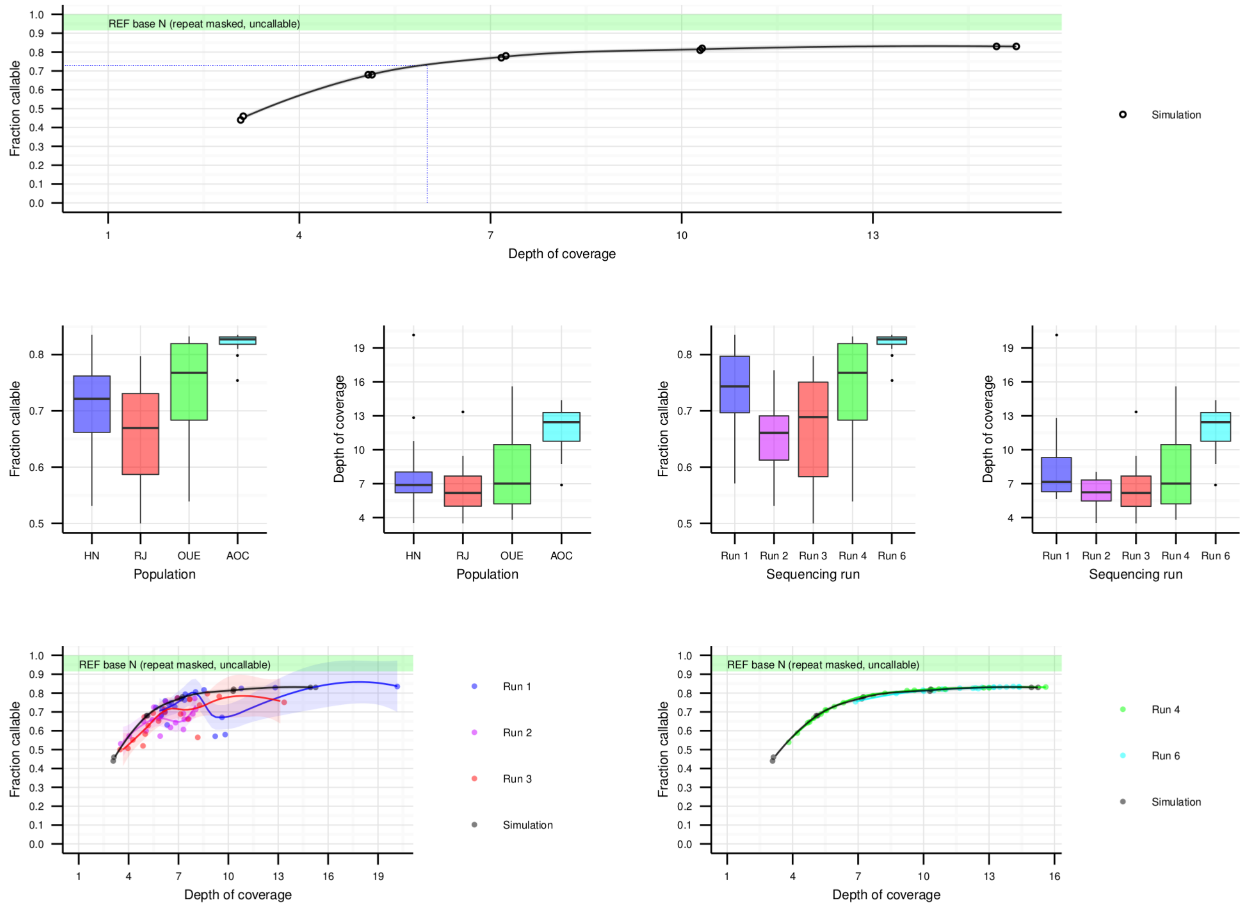

Supplementary Material SM1. Simulating optimal sequencing depth of coverage in drones

(**Top**) Modelling of depth of coverage (DP) against fraction of genome callable (GX) using two haploids down-sampled from ~ 15 X to 10, 7, 5 and 3 X. (**Middle, left to right**) Box plots indicating GX and DP in drones sequenced from 4 populations, followed by GX and DP of the same data by sequencing run. Run [n] comprised drones from [1] HN, [2] HN and RJ, [3] RJ, [4] OUE and [6] AOC. (**Bottom, left to right**) Fit of DP against GX to model by sequencing runs 1 to 3, followed by fit of DP against GX to model by sequencing runs 4 and 6.

Supplementary Material SM2 | Summary of SNPs identified in down-sampled haploids and diploids generated *in silico*

		Unique SNPs					Discrete SNPs[1]
**Dataset**	**Mean depth**	**Haploid homozygous**		**Haploid heterozygous**		**Haploid total[2]**	
**Diploid**	6.2	1,149,434					37,847
**H1**	3.12	772,779	1,322,321	14,752	30,214	1,349,829	237,342
**H2**	3.08	771,563		18,141			
**Diploid**	10.22	1,516,673					25,032
**H1**	5.14	985,327	1,642,746	27,132	56,894	1,691,775	200,134
**H2**	5.08	1,012,264		34,659			
**Diploid**	14.4	1,693,600					25,183
**H1**	7.17	1,069,895	1,770,285	35,618	74,920	1,834,895	166,478
**H2**	7.24	1,112,986		46,129			
**Diploid**	20.6	1,809,516					28,803
**H1**	10.29	1,125,634	1,849,796	42,599	85,756	1,925,064	144,351
**H2**	10.32	1,170,870		52,153			
**Diploid**	30.18	1,874,333					30,358
**H1**	15.25	1,165,508	1,906,785	47,535	92,204	1,989,329	145,354
**H2**	14.94	1,210,684		55,594			

[1] Discrete SNPs are those exclusive to either the haploids or diploid

[2] Figure is less than homozygous + heterozygous because some homozygous SNPs in one haploid are heterozygous in the other

References

1. Li, H. Aligning sequence reads, clone sequences and assembly contigs with BWA-MEM. *arXiv:1303.3997 [q-bio]* (2013). at <http://arxiv.org/abs/1303.3997>
2. McKenna, A. *et al.* The Genome Analysis Toolkit: A MapReduce framework for analyzing next-generation DNA sequencing data. *Genome Res.* **20,** 1297–1303 (2010).
3. Li, H. *et al.* The Sequence Alignment/Map format and SAMtools. *Bioinformatics* **25,** 2078–2079 (2009).
4. Rimmer, A. *et al.* Integrating mapping-, assembly- and haplotype-based approaches for calling variants in clinical sequencing applications. *Nat. Genet.* **46,** 912–918 (2014).
5. Cantarel, B. L. *et al.* BAYSIC: a Bayesian method for combining sets of genome variants with improved specificity and sensitivity. *BMC Bioinformatics* **15,** 104 (2014).
6. Browning, S. R. & Browning, B. L. Rapid and Accurate Haplotype Phasing and Missing-Data Inference for Whole-Genome Association Studies By Use of Localized Haplotype Clustering. *The American Journal of Human Genetics* **81,** 1084–1097 (2007).

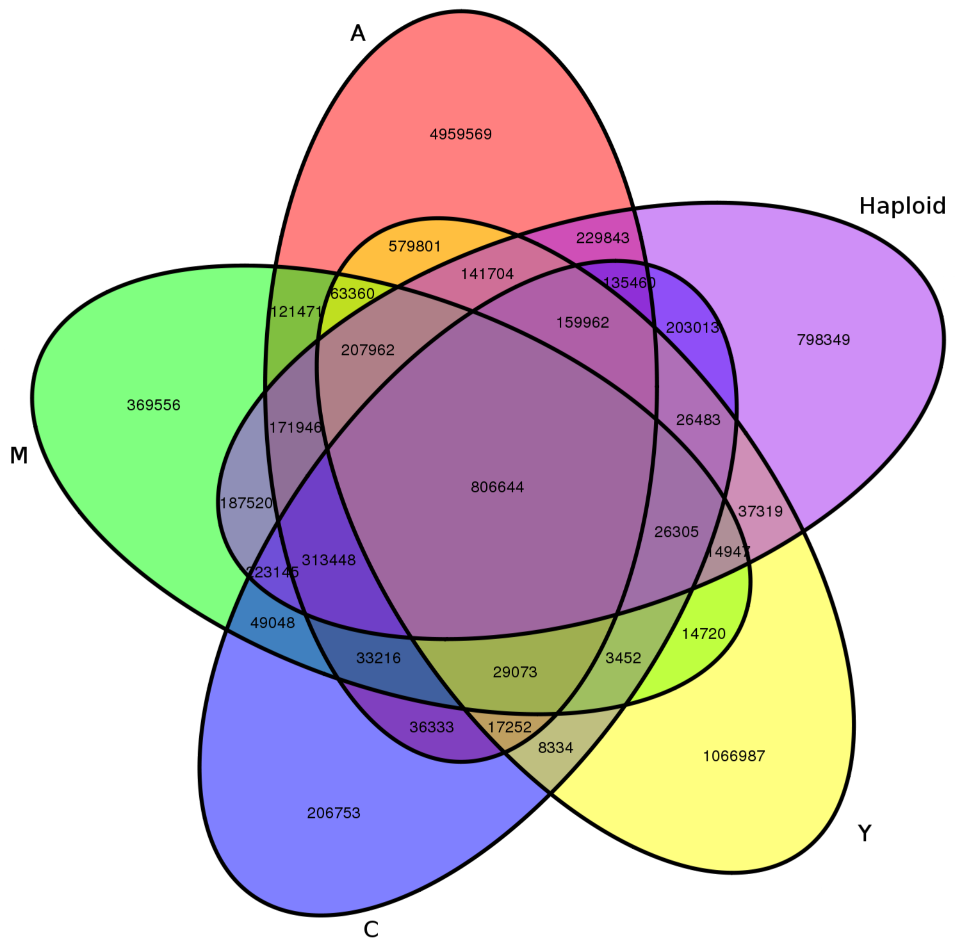

SF1. Venn diagram of SNPs called in each reference lineage and haploid data

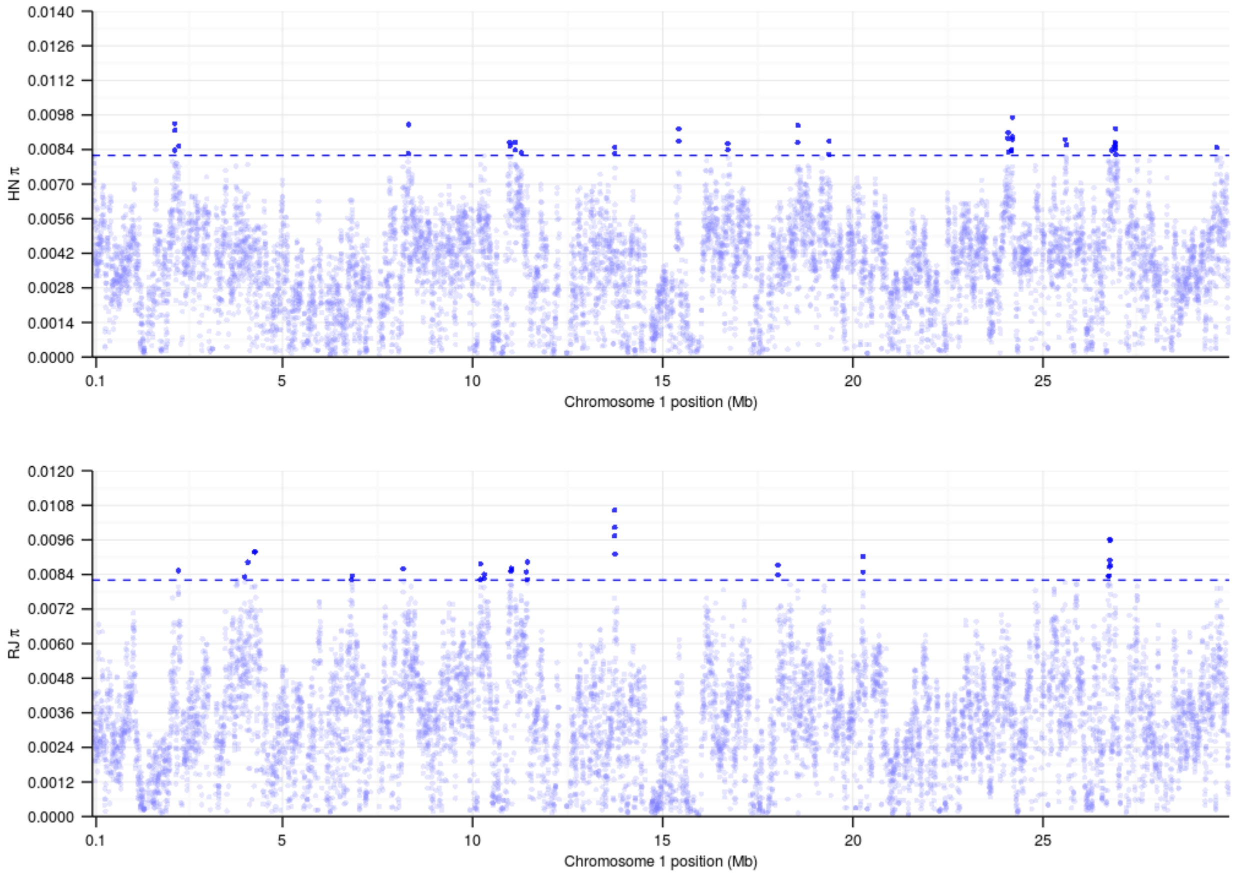

SF2. Nucleotide diversity in 12 kb bins along chromosome 1

Horizontal line indicates 99th percentile

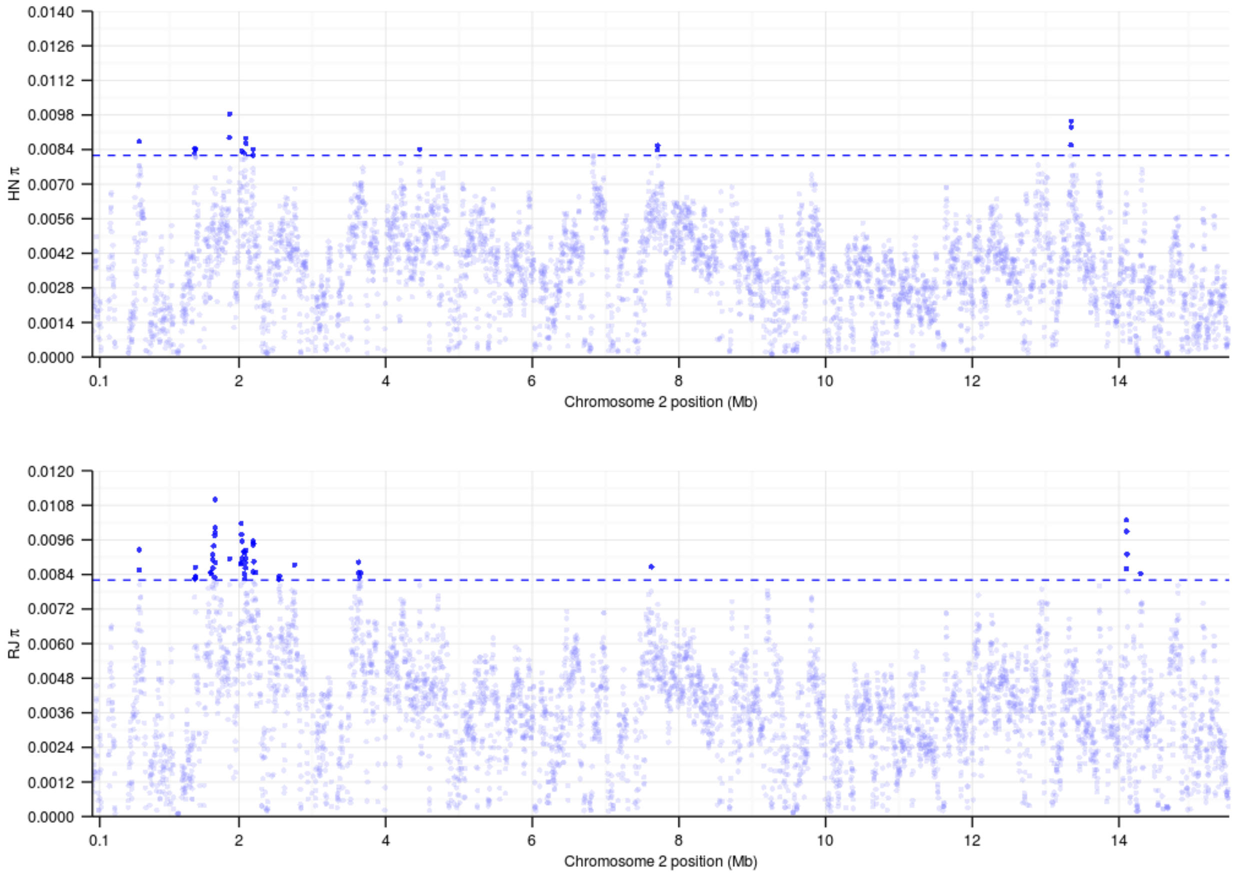

SF3. Nucleotide diversity in 12 kb bins along chromosome 2

Horizontal line indicates 99th percentile

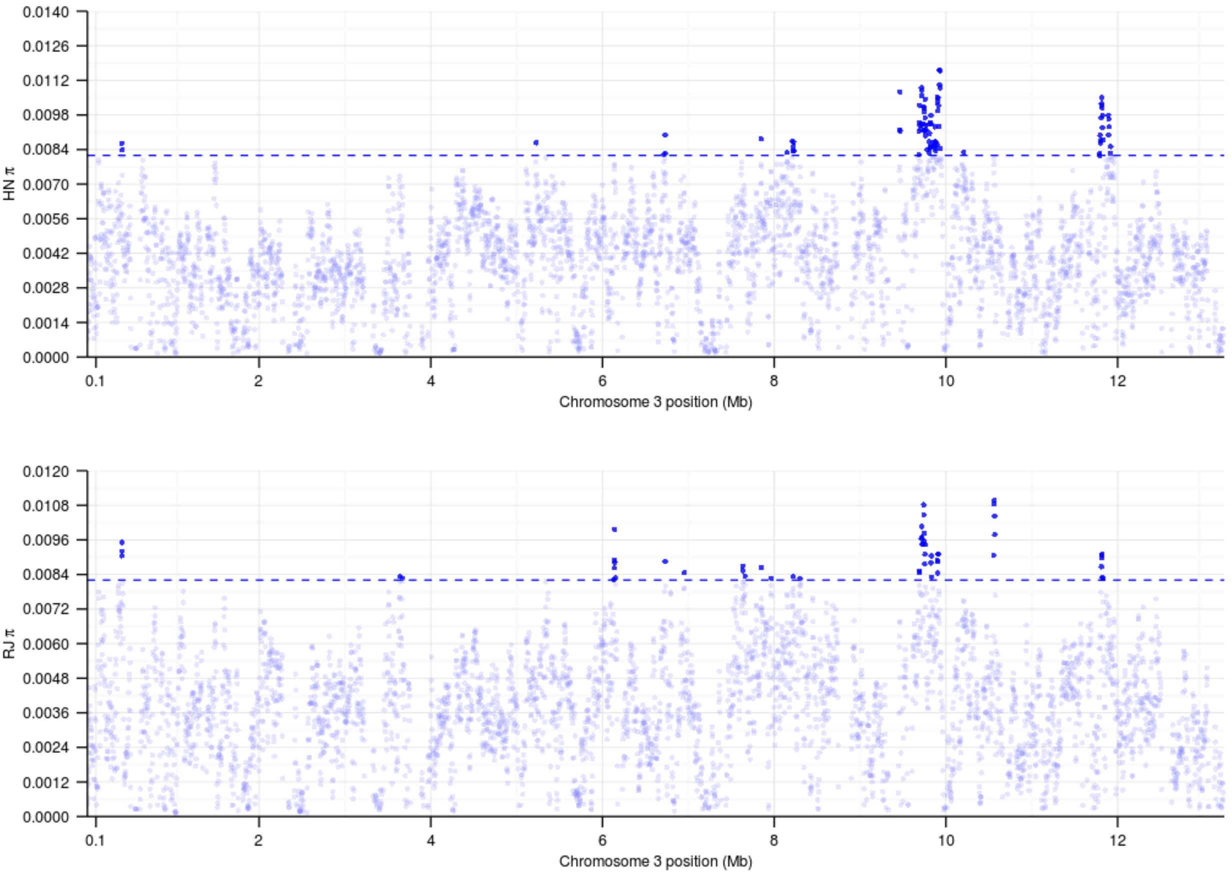

SF4. Nucleotide diversity in 12 kb bins along chromosome 3

Horizontal line indicates 99th percentile

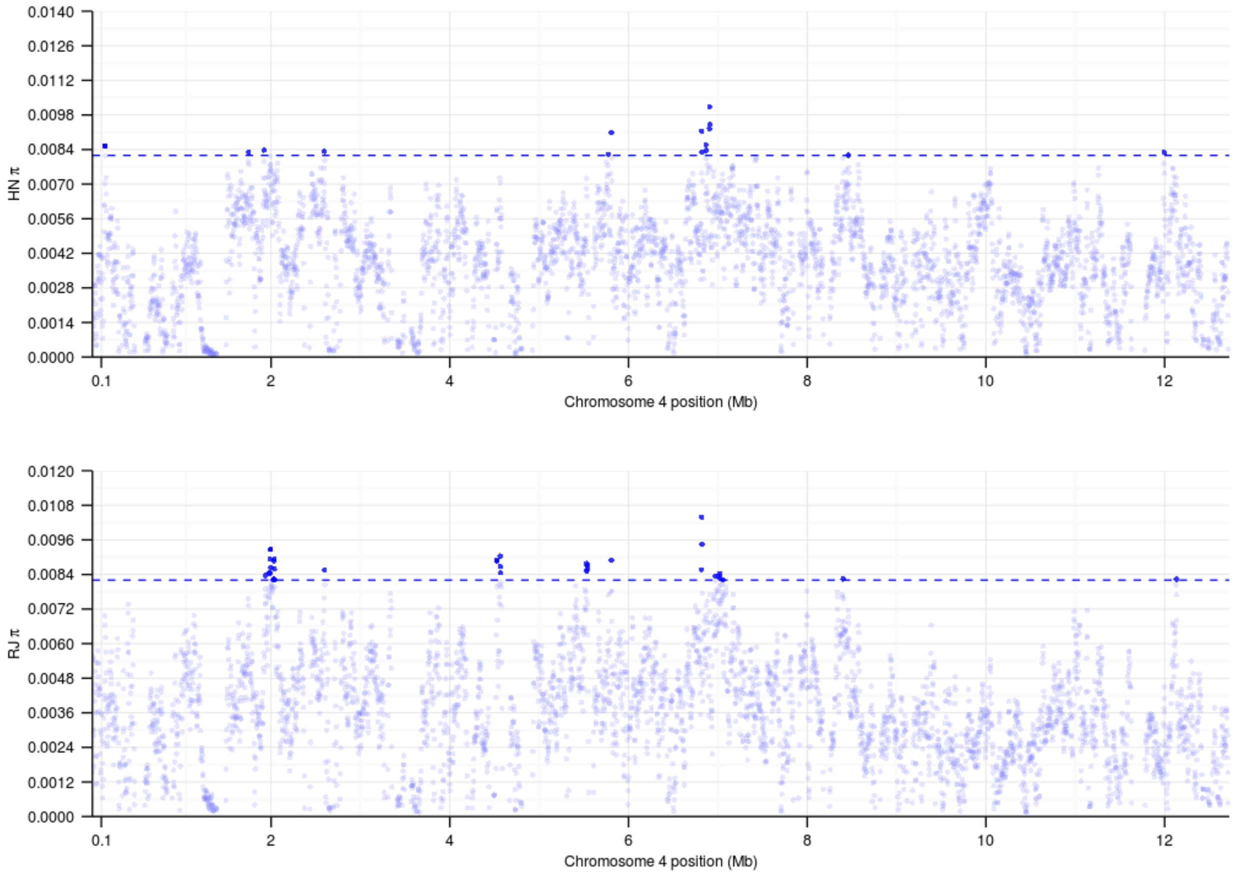

SF5. Nucleotide diversity in 12 kb bins along chromosome 4

Horizontal line indicates 99th percentile

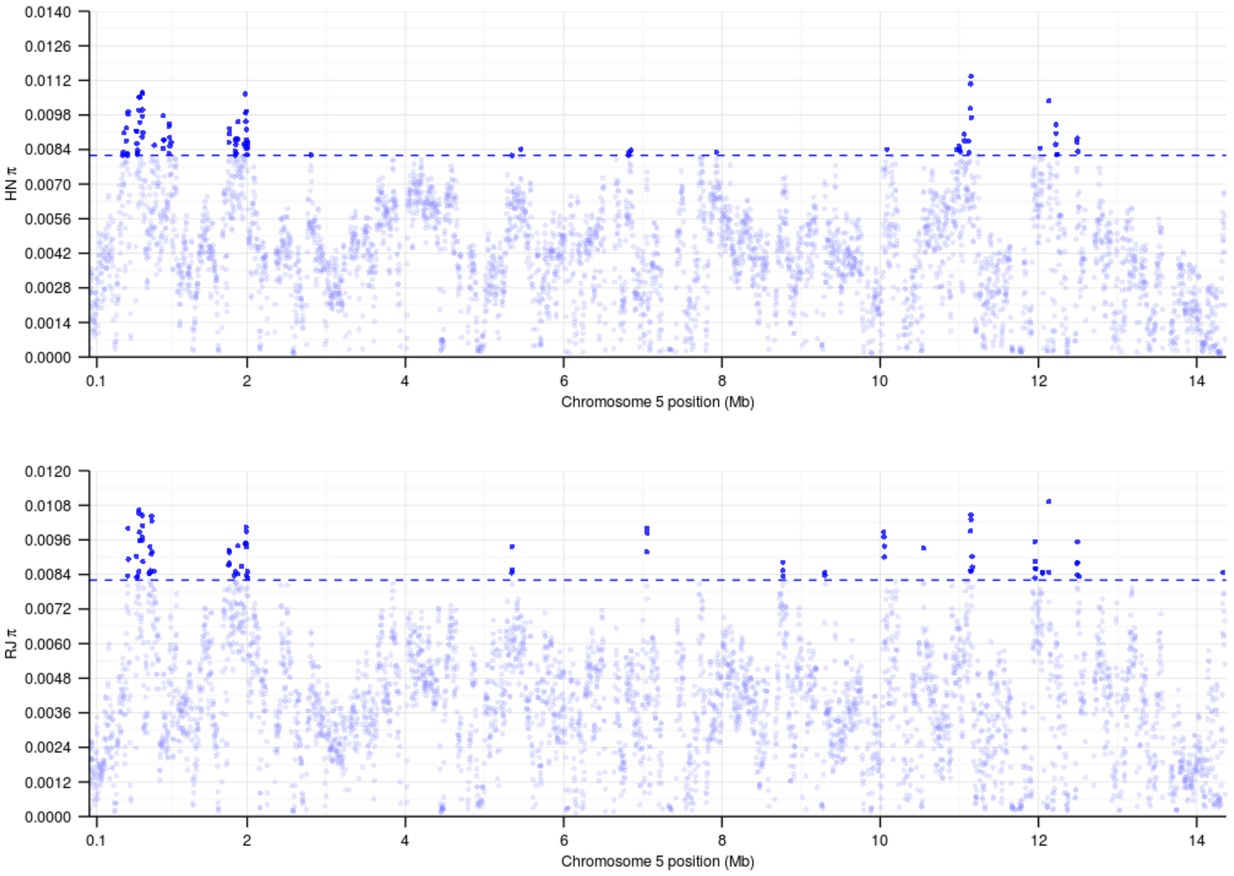

SF6. Nucleotide diversity in 12 kb bins along chromosome 5

Horizontal line indicates 99th percentile

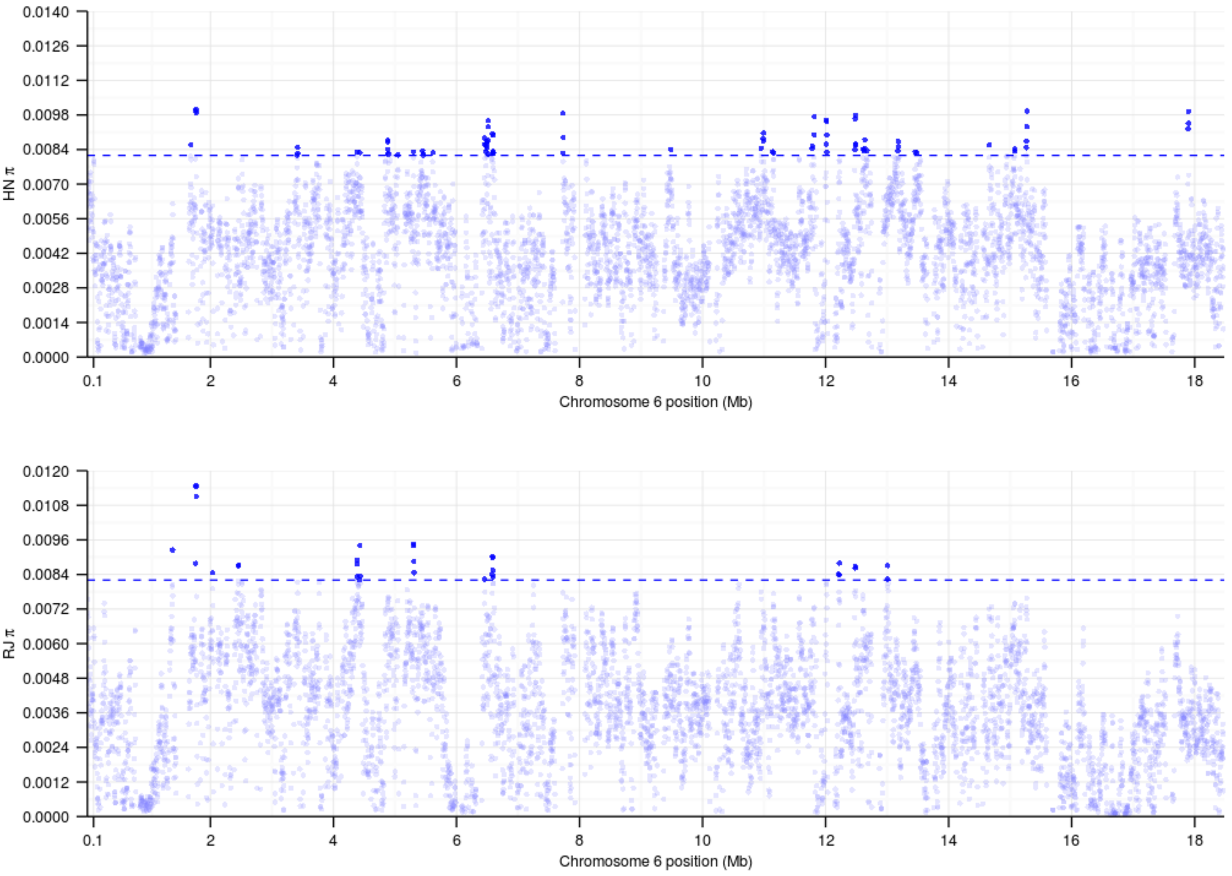

SF7. Nucleotide diversity in 12 kb bins along chromosome 6

Horizontal line indicates 99th percentile

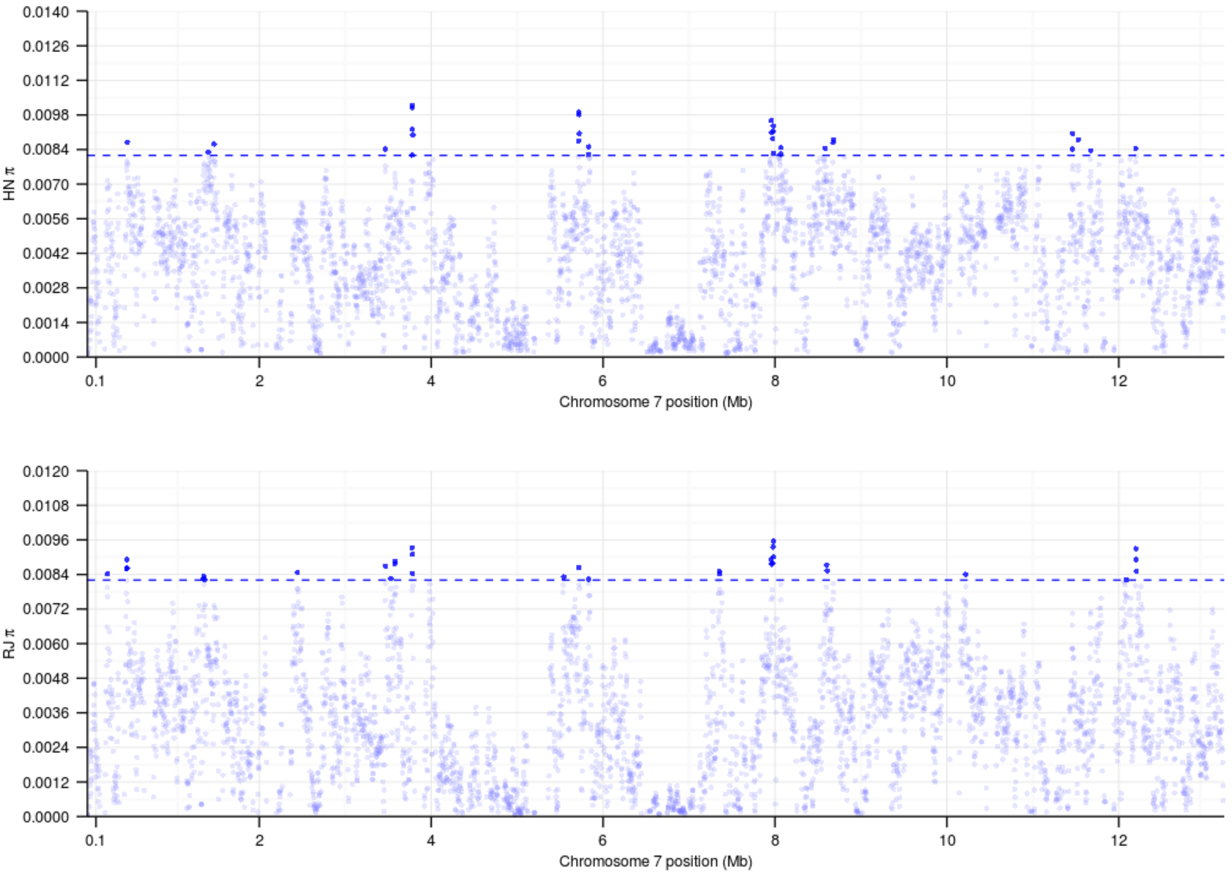

SF8. Nucleotide diversity in 12 kb bins along chromosome 7

Horizontal line indicates 99th percentile

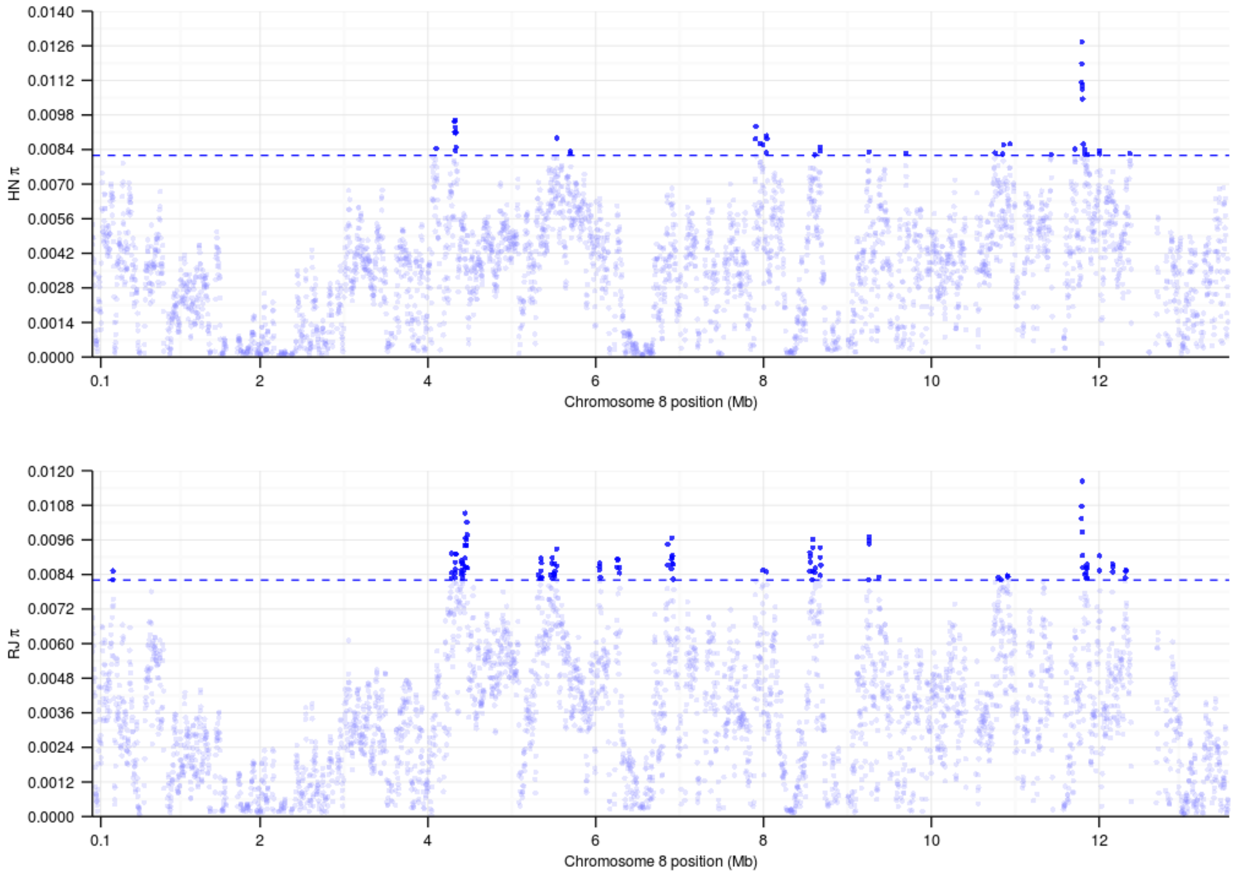

SF9. Nucleotide diversity in 12 kb bins along chromosome 8

Horizontal line indicates 99th percentile

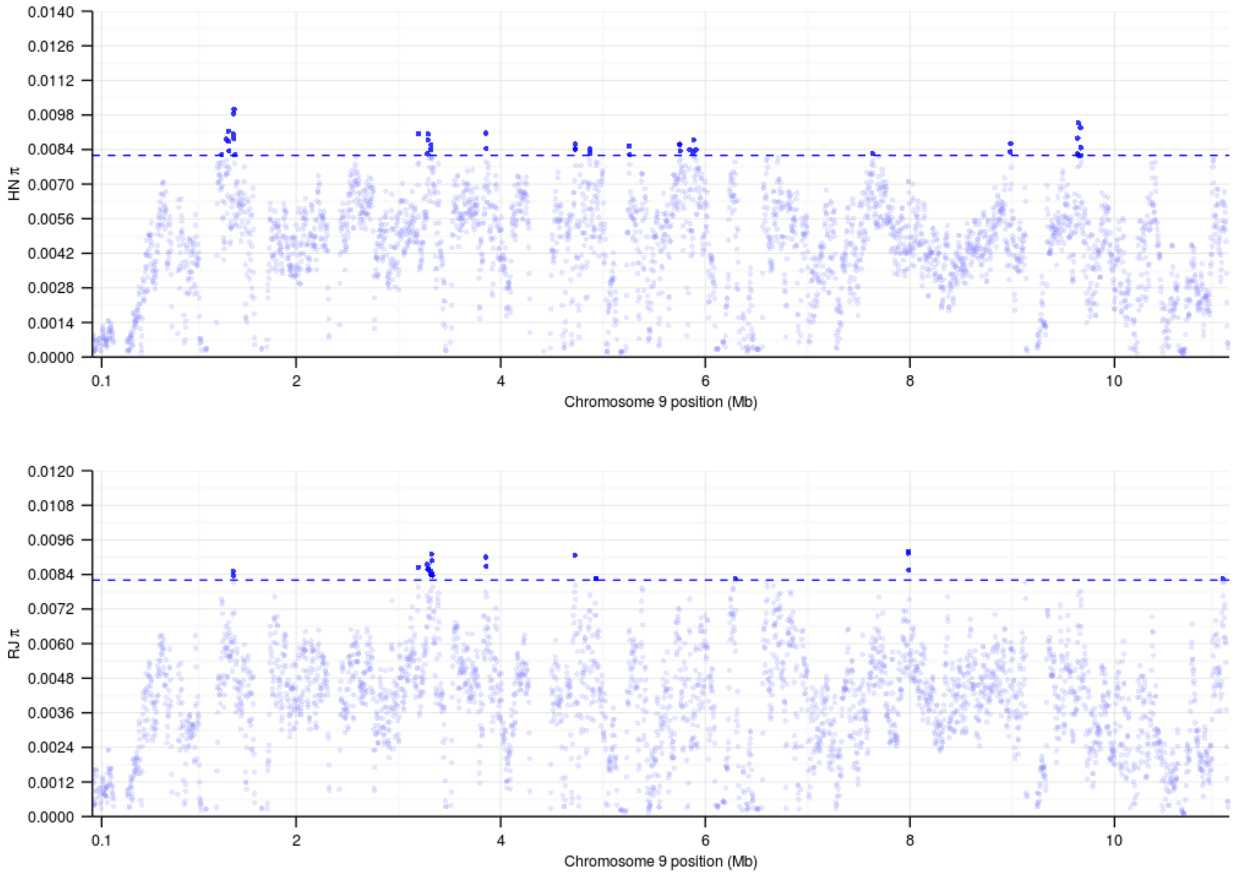

SF10. Nucleotide diversity in 12 kb bins along chromosome 9

Horizontal line indicates 99th percentile

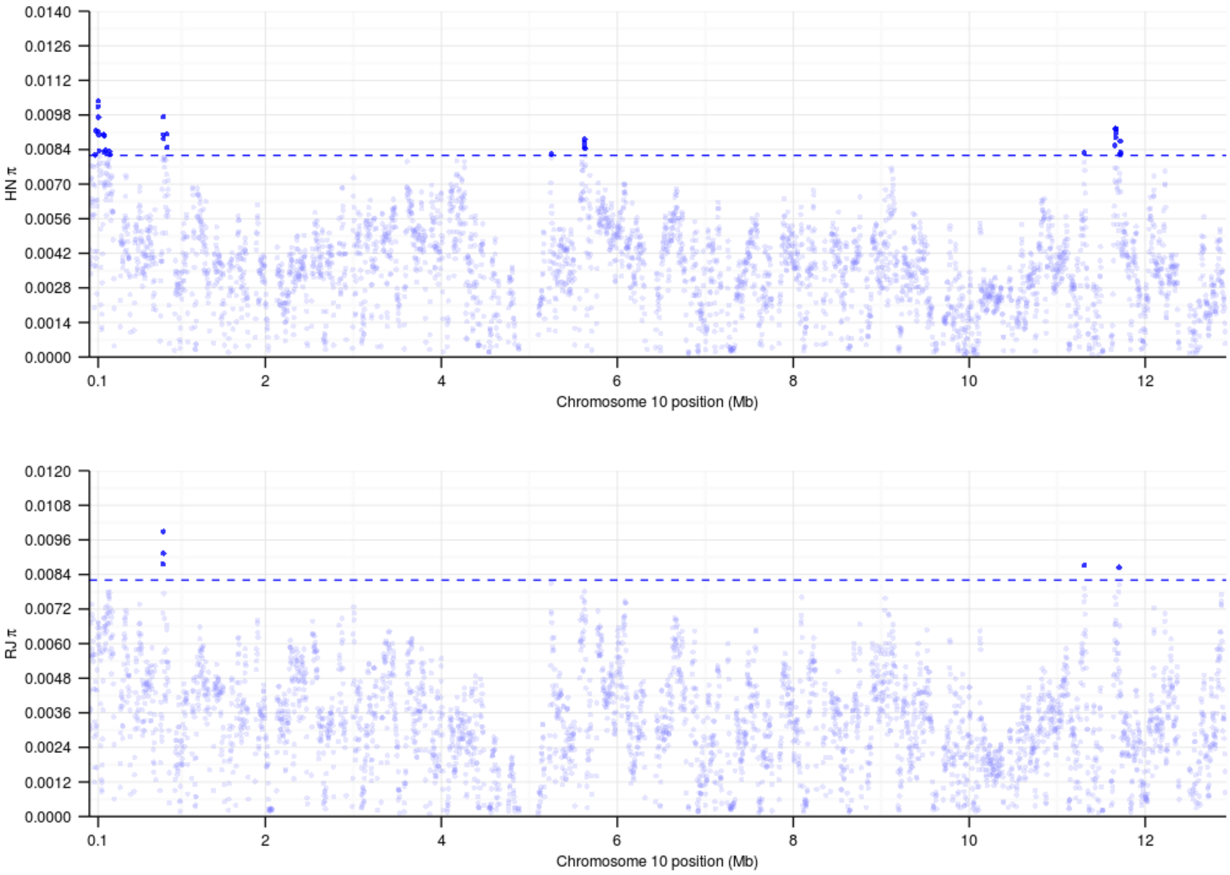

SF11. Nucleotide diversity in 12 kb bins along chromosome 10

Horizontal line indicates 99th percentile

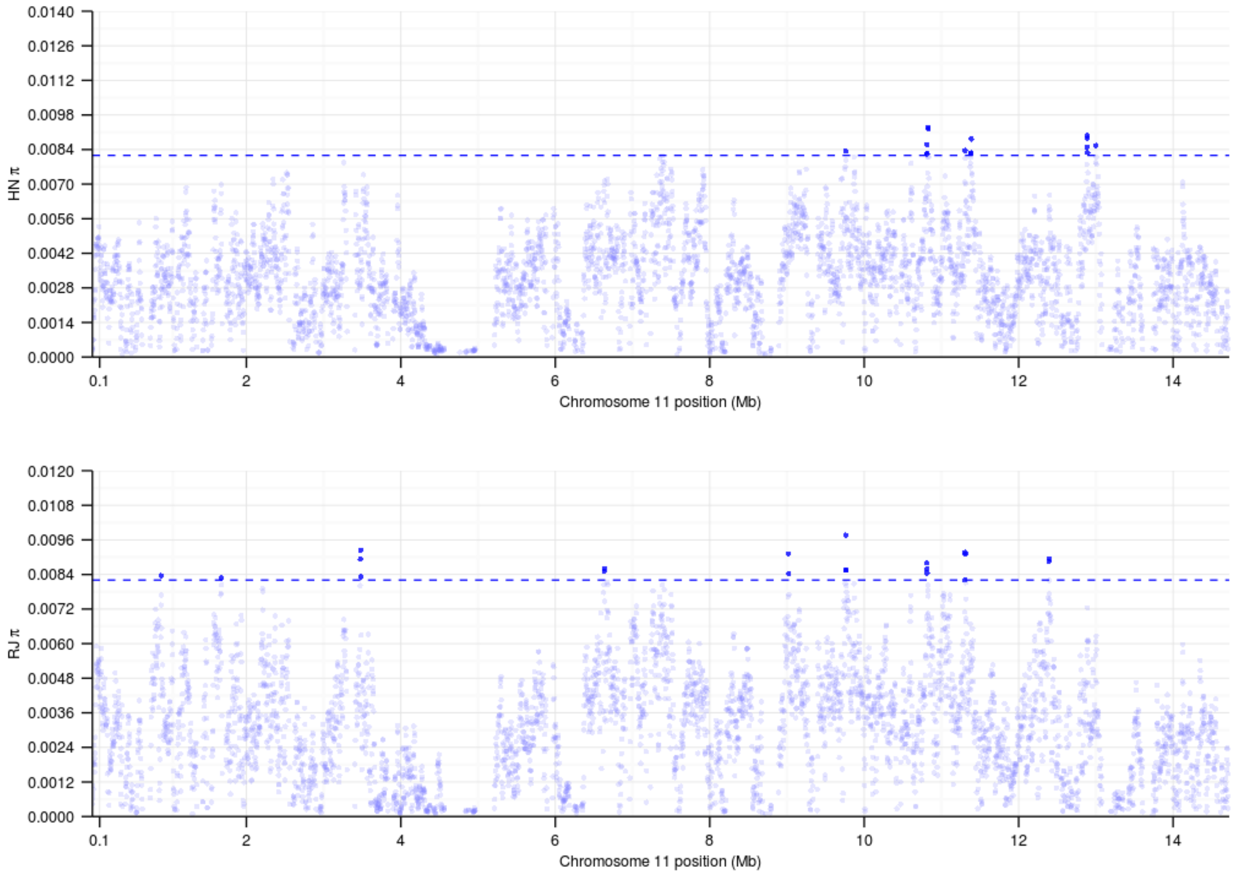

SF12. Nucleotide diversity in 12 kb bins along chromosome 11

Horizontal line indicates 99th percentile

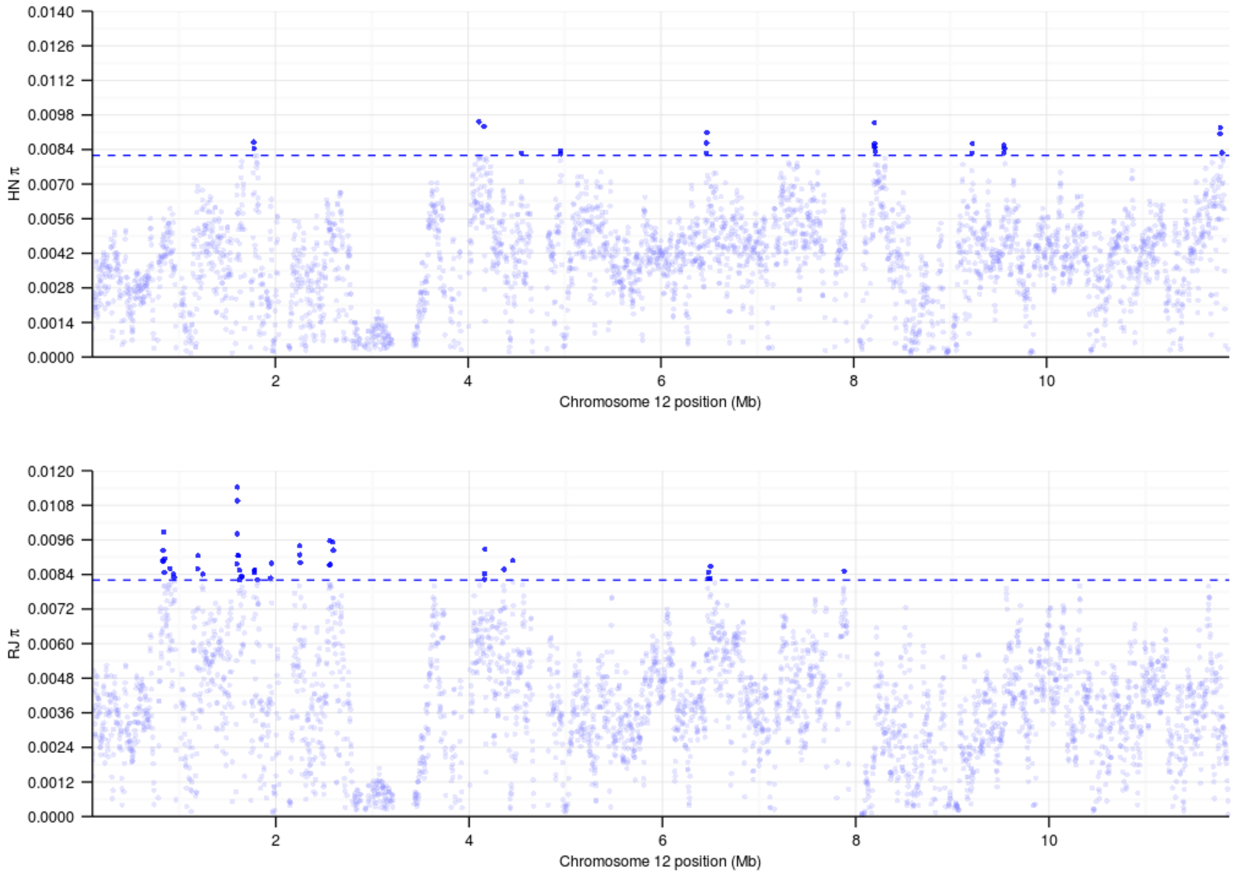

SF13. Nucleotide diversity in 12 kb bins along chromosome 12

Horizontal line indicates 99th percentile

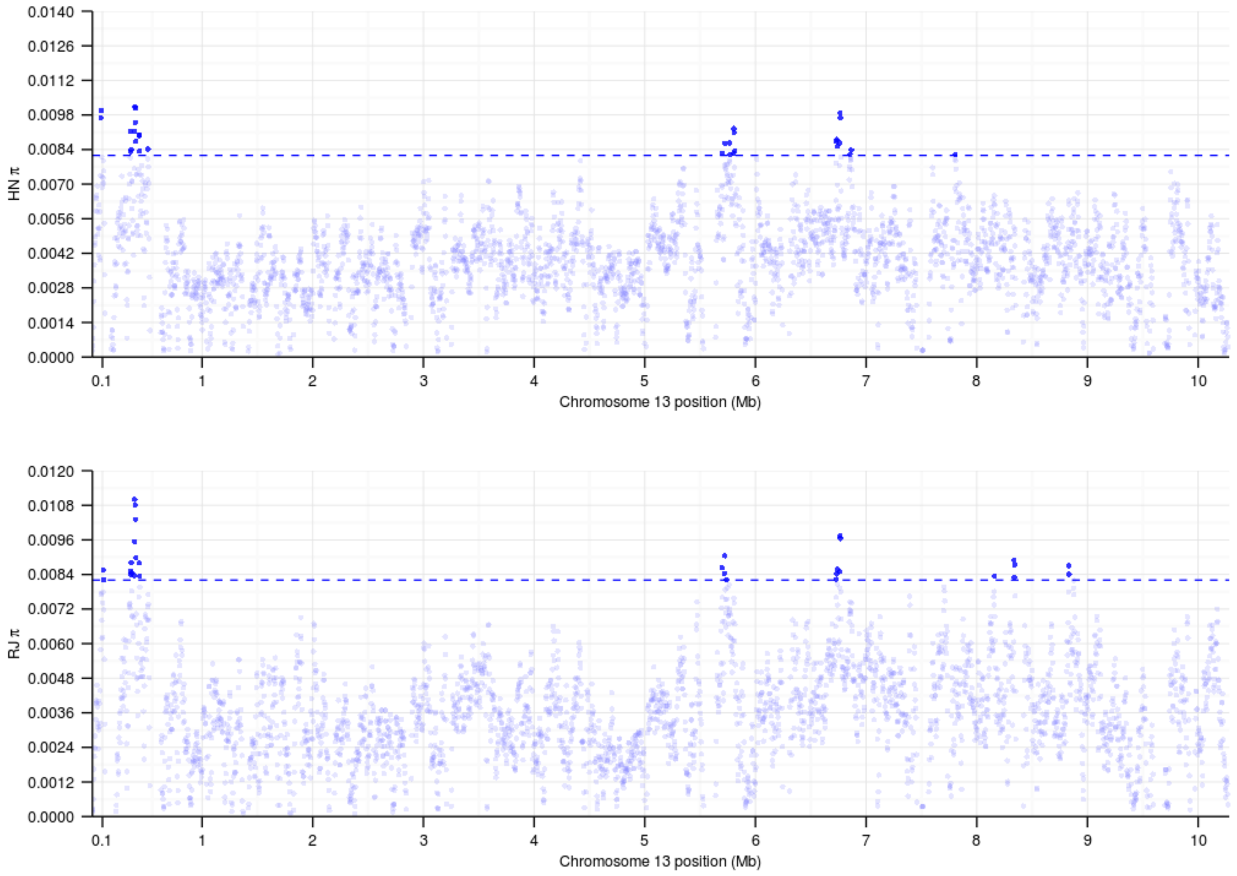

SF14. Nucleotide diversity in 12 kb bins along chromosome 13

Horizontal line indicates 99th percentile

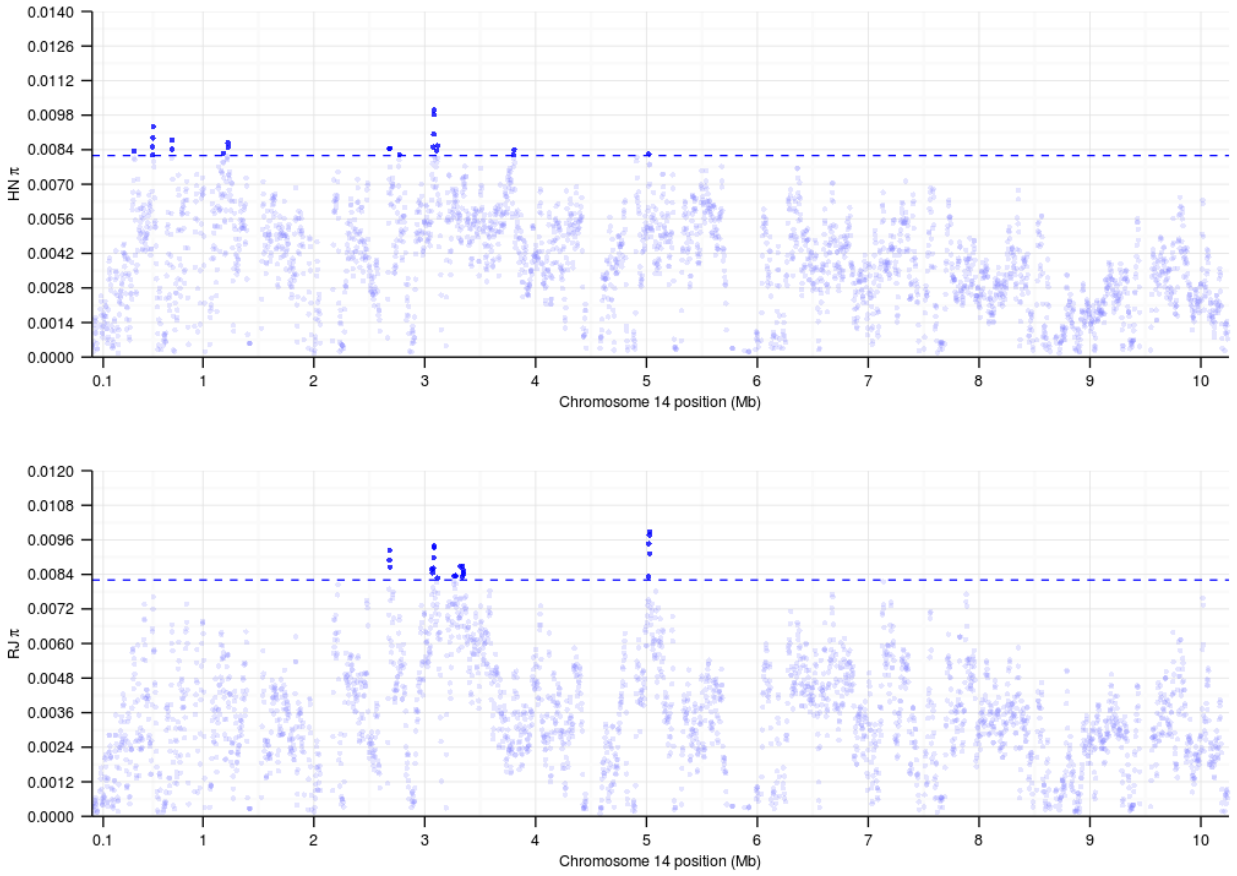

SF15. Nucleotide diversity in 12 kb bins along chromosome 14

Horizontal line indicates 99th percentile

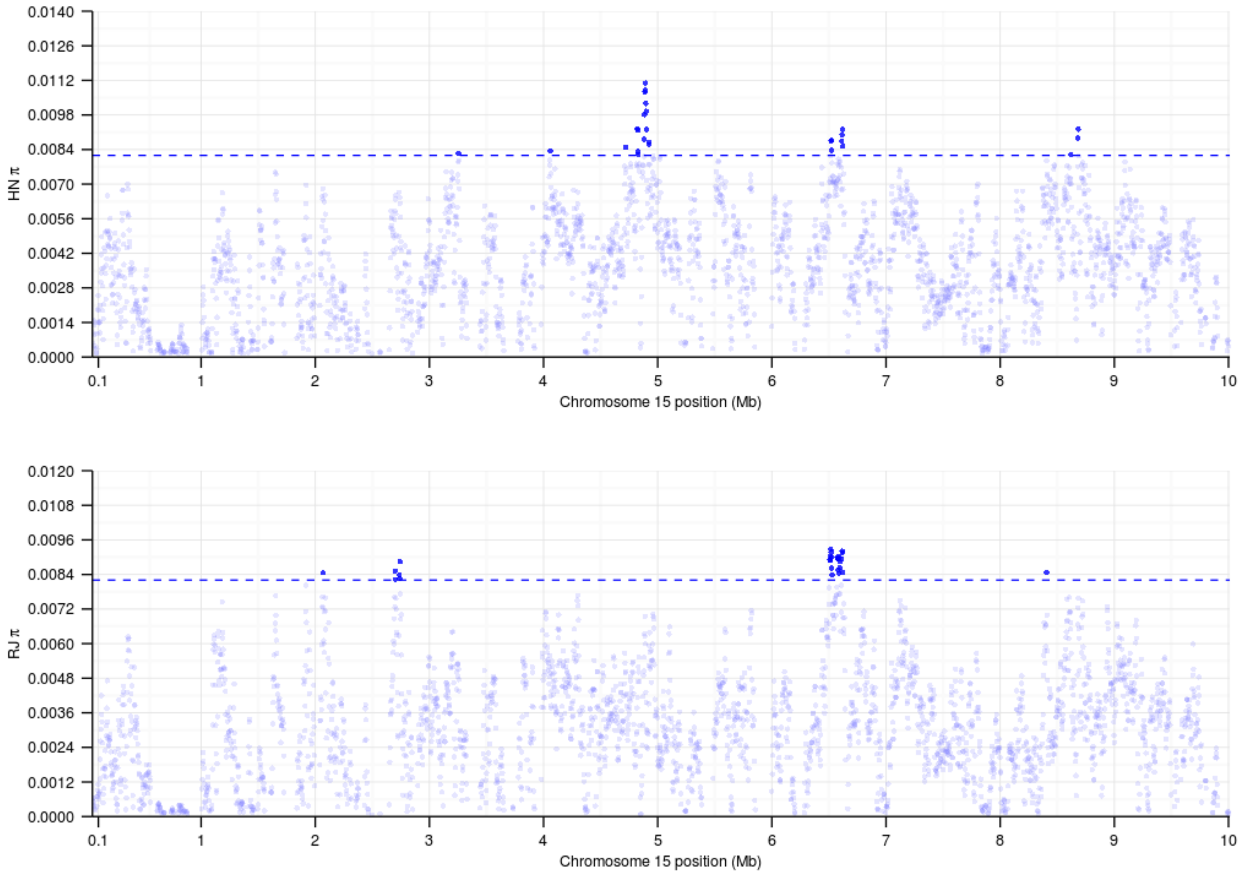

SF16. Nucleotide diversity in 12 kb bins along chromosome 15

Horizontal line indicates 99th percentile

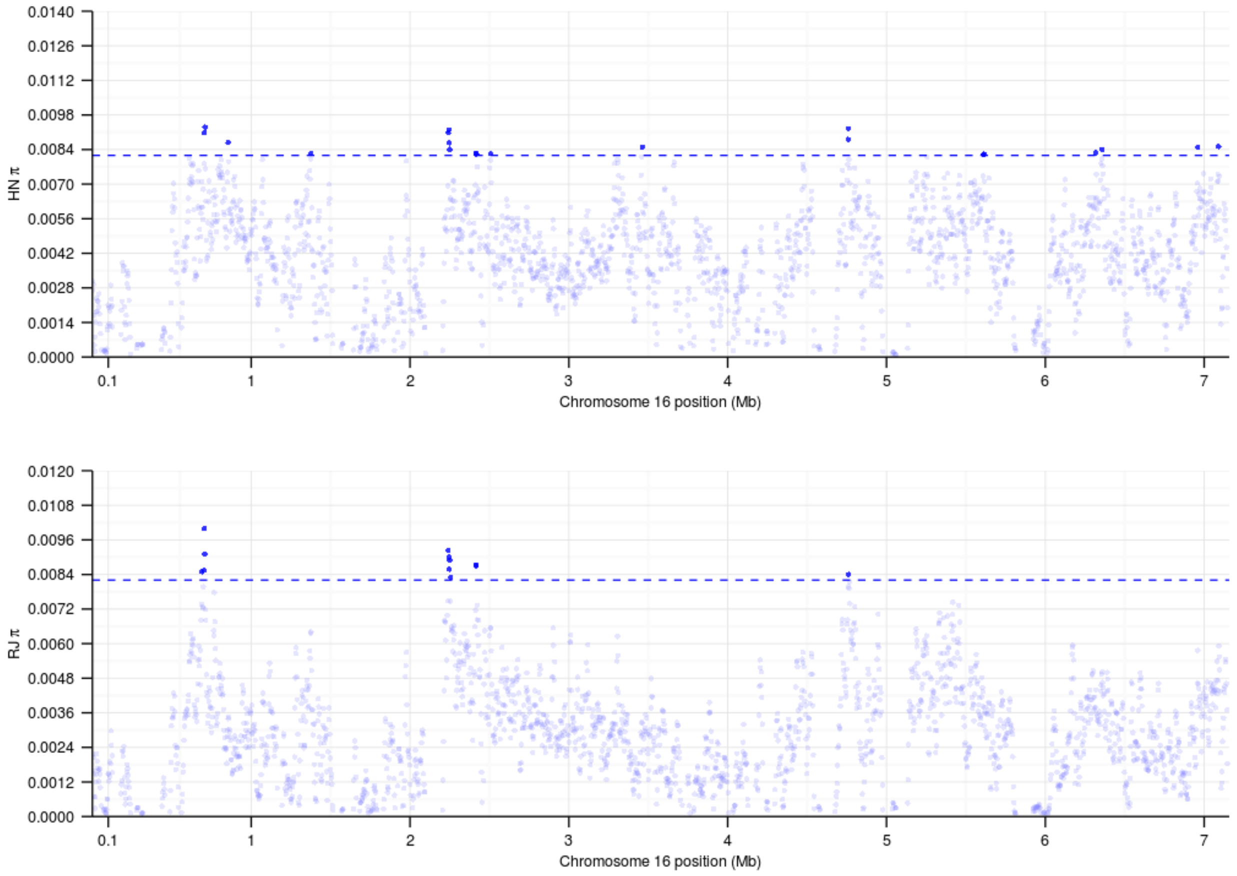

SF17. Nucleotide diversity in 12 kb bins along chromosome 16

Horizontal line indicates 99th percentile

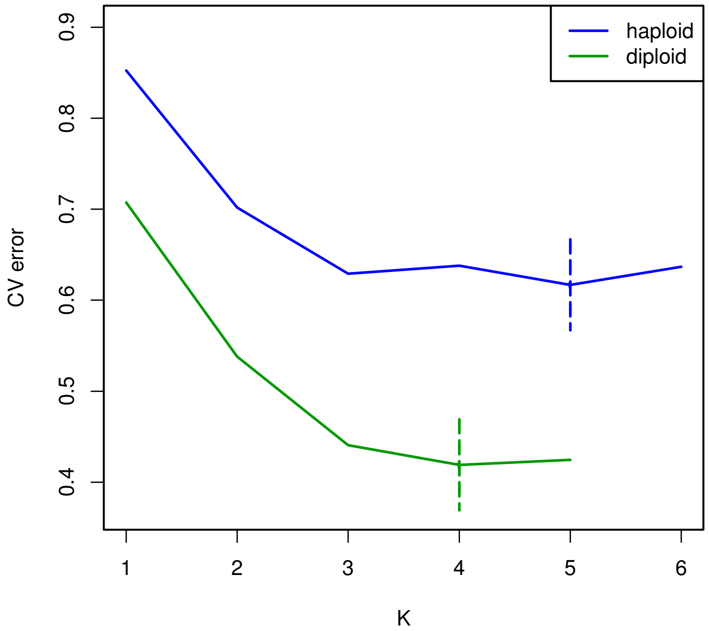

SF18. Plot of CV error from ADMIXTURE analyses

The haploid data range comprises drones from HN and RJ populations together with diploid reference workers. The diploid data range comprises diploids generate *in silico* by merging alleles of haploids within each population, together with diploid reference workers.

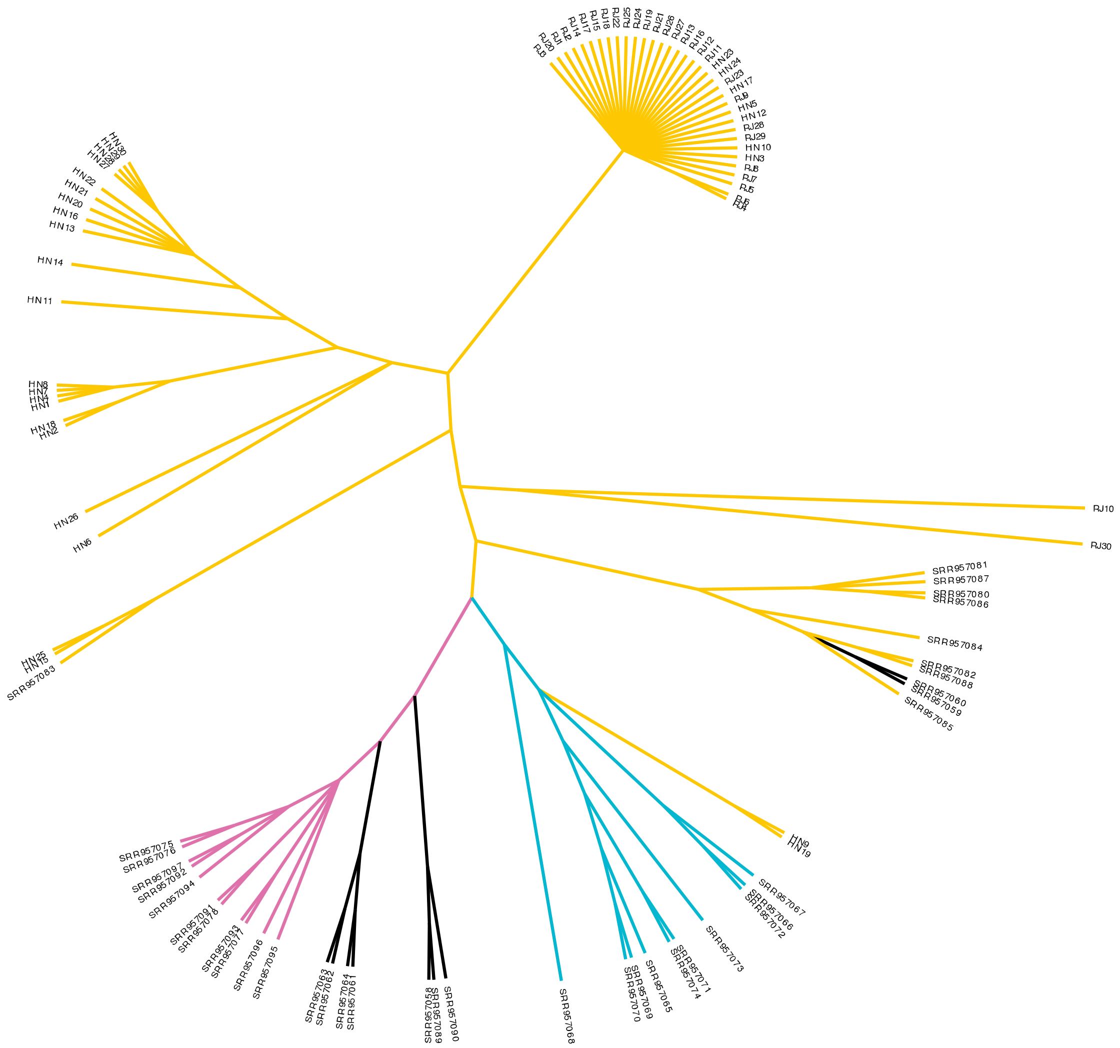

SF19. Phylogenetic tree constructed from mitochondrial genome sequence

The mitochondrial genome sequence was generated for each individual following alignment of reads to the reference genome. Sequences were subsequently aligned with Clustal W and the multiple alignment analysed using jmodeltest2 to identify the best-fit nucleotide substitution model. This was used to configure MrBayes which was run for 2 M generations, and the results plotted using FigTree. Branches are coloured according to the sample assignments by ADMIXTURE (Fig 2A).

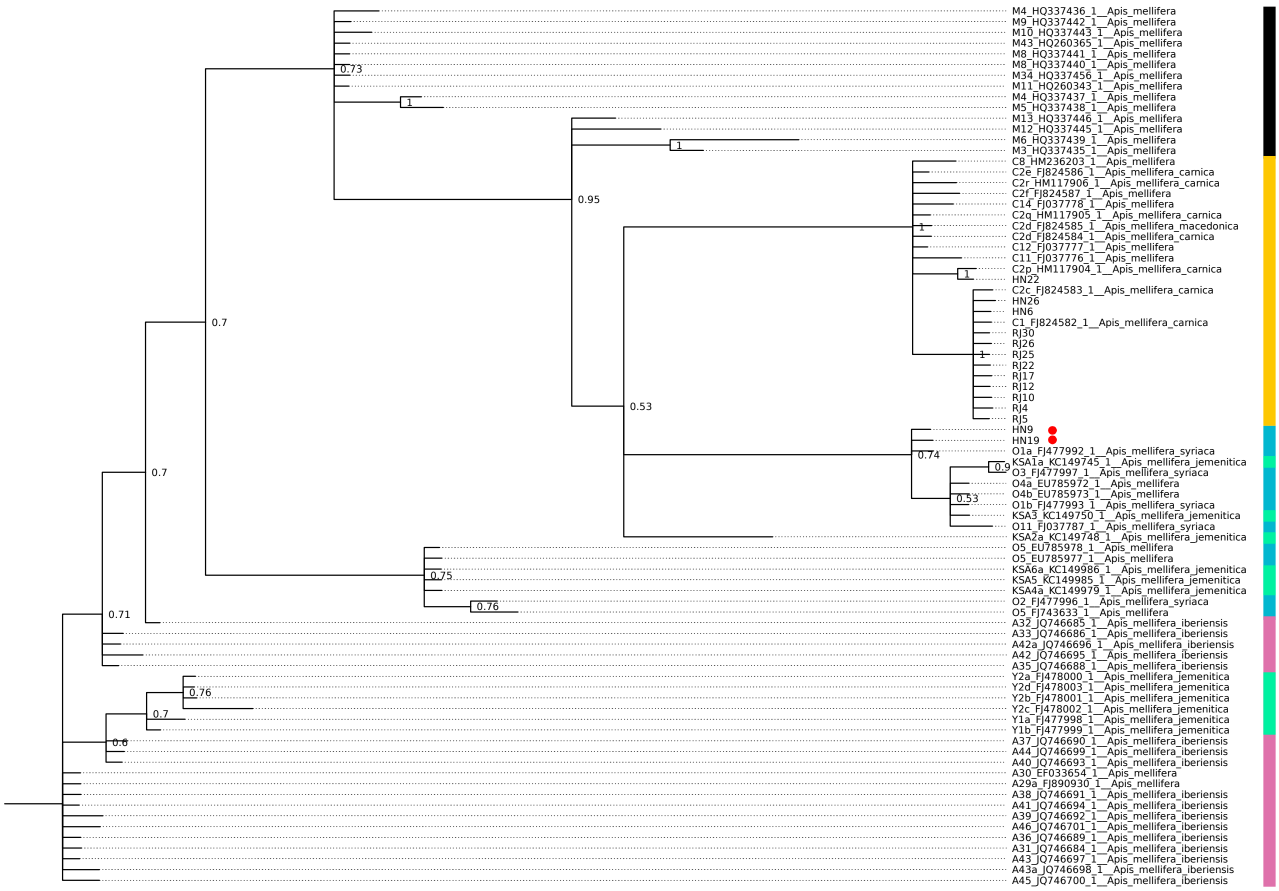

SF20. Phylogenetic tree constructed from mitochondrial tRNA^{Leu}-cox2 intergenic sequence

The intergenic tRNA^{leu}-cox2 region for several drones was amplified using primers E2 and H2 and Sanger sequenced. Sequences were processed using FinchTV and aligned using MAFFT. The sequences were aligned against 68 reference haplotypes downloaded from GenBank. Multiple alignment was processed with MrBayes to generate a consensus which was plotted using FigTree. Coloured bars on the right of the plot indicate lineage: A (pink), M (black), C (orange), O (cyan), Y (green). The two HN samples with O lineage mitotypes are highlighted with red circles.

```
                      1 [        .         .         .         .         :         .         .         .         .         1 100
 1 HN9|Sanger  100.0%    cttttattaaaattaataaattaatataaaataaaataaaatataacaaaatatatttattaaaacttaatttattaaaattt-cccacttaattcatat
 2 HN9|NGS     100.0%    cttttattaaaattaataaattaatataaaataaaataaaatataacaaaatatatttattaaaacttaatttattaaaattt-cccacttaattcatat
 3 HN19|Sanger 100.0%    cttttattaaaattaataaattaatataaaataaaataaaatataacaaaatatatttattaaaacttaatttattaaaattt-cccacttaattcatat
 4 HN19|NGS    100.0%    cttttattaaaattaataaattaatataaaataaaataaaatataacaaaatatatttattaaaacttaatttattaaaattt-cccacttaattcatat
 5 HN22|Sanger  73.8%    cttttattaaa--------------------------------------------------------------------attt-cccacttaattcatat
 6 HN22|NGS     73.8%    cttttattaaa--------------------------------------------------------------------attt-cccacttaattcatat
 7 HN26|Sanger  74.1%    cttttattaaa--------------------------------------------------------------------attt-cccacttaattcatat
 8 HN26|NGS     74.1%    cttttattaaa--------------------------------------------------------------------attt-cccacttaattcatat
 9 HN6|Sanger   74.1%    cttttattaaa--------------------------------------------------------------------attt-cccacttaattcatat
10 HN6|NGS      74.1%    cttttattaaa--------------------------------------------------------------------attt-cccacttaattcatat
11 RJ30|Sanger  73.9%    cttttattaaa--------------------------------------------------------------------atttccccacttaattcatat
12 RJ30|NGS     73.9%    cttttattaaa--------------------------------------------------------------------atttccccacttaattcatat
13 RJ26|Sanger  73.9%    cttttattaaa--------------------------------------------------------------------atttccccacttaattcatat
14 RJ26|NGS     73.9%    cttttattaaa--------------------------------------------------------------------atttccccacttaattcatat
15 RJ25|Sanger  73.9%    cttttattaaa--------------------------------------------------------------------atttccccacttaattcatat
16 RJ25|NGS     73.9%    cttttattaaa--------------------------------------------------------------------atttccccacttaattcatat
17 RJ22|Sanger  73.9%    cttttattaaa--------------------------------------------------------------------atttccccacttaattcatat
18 RJ22|NGS     73.9%    cttttattaaa--------------------------------------------------------------------atttccccacttaattcatat
19 RJ17|Sanger  73.9%    cttttattaaa--------------------------------------------------------------------atttccccacttaattcatat
20 RJ17|NGS     73.9%    cttttattaaa--------------------------------------------------------------------atttccccacttaattcatat
21 RJ12|Sanger  73.9%    cttttattaaa--------------------------------------------------------------------atttccccacttaattcatat
22 RJ12|NGS     73.9%    cttttattaaa--------------------------------------------------------------------atttccccacttaattcatat
23 RJ3|Sanger   73.9%    cttttattaaa--------------------------------------------------------------------atttccccacttaattcatat
24 RJ3|NGS      73.9%    cttttattaaa--------------------------------------------------------------------atttccccacttaattcatat
25 RJ2|Sanger   73.9%    cttttattaaa--------------------------------------------------------------------atttccccacttaattcatat
26 RJ2|NGS      73.9%    cttttattaaa--------------------------------------------------------------------atttccccacttaattcatat
27 RJ10|Sanger  73.9%    cttttattaaa--------------------------------------------------------------------atttccccacttaattcatat
28 RJ10|NGS     73.9%    cttttattaaa--------------------------------------------------------------------atttccccacttaattcatat

                    101          .         .         .         .         :         .         .         .         .         2 200
 1 HN9|Sanger  100.0%    taatttaaaaataaattaaataacaattttaataaaataaataataattttattttttatattgaattttaaattcaatcttaaagatttaatcttttt
 2 HN9|NGS     100.0%    taatttaaaaataaattaaataacaattttaataaaataaataataattttattttttatattgaattttaaattcaatcttaaagatttaatcttttt
 3 HN19|Sanger 100.0%    taatttaaaaataaattaaataacaattttaataaaataaataataattttattttttatattgaattttaaattcaatcttaaagatttaatcttttt
 4 HN19|NGS    100.0%    taatttaaaaataaattaaataacaattttaataaaataaataataattttattttttatattgaattttaaattcaatcttaaagatttaatcttttt
 5 HN22|Sanger  73.8%    taatttaaaaataaatt-aataacaattttaataaaataaataattaattttattttttatattgaatttaaaattcaatcttaaagatttaatcttttt
 6 HN22|NGS     73.8%    taatttaaaaataaatt-aataacaattttaataaaataaataattaattttattttttatattgaatttaaaattcaatcttaaagatttaatcttttt
 7 HN26|Sanger  74.1%    taatttaaaaataaatt-aataacaattttaataaaataaataattaattttattttttatattgaattttaaattcaatcttaaagatttaatcttttt
 8 HN26|NGS     74.1%    taatttaaaaataaatt-aataacaattttaataaaataaataattaattttattttttatattgaattttaaattcaatcttaaagatttaatcttttt
 9 HN6|Sanger   74.1%    taatttaaaaataaatt-aataacaattttaataaaataaataattaattttattttttatattgaattttaaattcaatcttaaagatttaatcttttt
10 HN6|NGS      74.1%    taatttaaaaataaatt-aataacaattttaataaaataaataattaattttattttttatattgaattttaaattcaatcttaaagatttaatcttttt
11 RJ30|Sanger  73.9%    taatttaaaaataaatt-aataacaattttaataaaataaataattaattttattttttatattgaattttaaattcaatcttaaagatttaatcttttt
12 RJ30|NGS     73.9%    taatttaaaaataaatt-aataacaattttaataaaataaataattaattttattttttatattgaattttaaattcaatcttaaagatttaatcttttt
13 RJ26|Sanger  73.9%    taatttaaaaataaatt-aataacaattttaataaaataaataattaattttattttttatattgaattttaaattcaatcttaaagatttaatcttttt
14 RJ26|NGS     73.9%    taatttaaaaataaatt-aataacaattttaataaaataaataattaattttattttttatattgaattttaaattcaatcttaaagatttaatcttttt
15 RJ25|Sanger  73.9%    taatttaaaaataaatt-aataacaattttaataaaataaataattaattttattttttatattgaattttaaattcaatcttaaagatttaatcttttt
16 RJ25|NGS     73.9%    taatttaaaaataaatt-aataacaattttaataaaataaataattaattttattttttatattgaattttaaattcaatcttaaagatttaatcttttt
17 RJ22|Sanger  73.9%    taatttaaaaataaatt-aataacaattttaataaaataaataattaattttattttttatattgaattttaaattcaatcttaaagatttaatcttttt
18 RJ22|NGS     73.9%    taatttaaaaataaatt-aataacaattttaataaaataaataattaattttattttttatattgaattttaaattcaatcttaaagatttaatcttttt
19 RJ17|Sanger  73.9%    taatttaaaaataaatt-aataacaattttaataaaataaataattaattttattttttatattgaattttaaattcaatcttaaagatttaatcttttt
20 RJ17|NGS     73.9%    taatttaaaaataaatt-aataacaattttaataaaataaataattaattttattttttatattgaattttaaattcaatcttaaagatttaatcttttt
21 RJ12|Sanger  73.9%    taatttaaaaataaatt-aataacaattttaataaaataaataattaattttattttttatattgaattttaaattcaatcttaaagatttaatcttttt
22 RJ12|NGS     73.9%    taatttaaaaataaatt-aataacaattttaataaaataaataattaattttattttttatattgaattttaaattcaatcttaaagatttaatcttttt
23 RJ3|Sanger   73.9%    taatttaaaaataaatt-aataacaattttaataaaataaataattaattttattttttatattgaattttaaattcaatcttaaagatttaatcttttt
24 RJ3|NGS      73.9%    taatttaaaaataaatt-aataacaattttaataaaataaataattaattttattttttatattgaattttaaattcaatcttaaagatttaatcttttt
25 RJ2|Sanger   73.9%    taatttaaaaataaatt-aataacaattttaataaaataaataattaattttattttttatattgaattttaaattcaatcttaaagatttaatcttttt
26 RJ2|NGS      73.9%    taatttaaaaataaatt-aataacaattttaataaaataaataattaattttattttttatattgaattttaaattcaatcttaaagatttaatcttttt
27 RJ10|Sanger  73.9%    taatttaaaaataaatt-aataacaattttaataaaataaataattaattttattttttatattgaattttaaattcaatcttaaagatttaatcttttt
28 RJ10|NGS     73.9%    taatttaaaaataaatt-aataacaattttaataaaataaataattaattttattttttatattgaattttaaattcaatcttaaagatttaatcttttt

                    201          .         .         .         .         :         .         .         . ] 283
 1 HN9|Sanger  100.0%    attaaaattaataaattaatataaaataaaataaaatataacagaatatatttattaaaacttaatttattaaaatttccaca
 2 HN9|NGS     100.0%    attaaaattaataaattaatataaaataaaataaaatataacagaatatatttattaaaacttaatttattaaaatttccaca
 3 HN19|Sanger 100.0%    attaaaattaataaattaatataaaataaaataaaatataacagaatatatttattaaaacttaatttattaaaatttccaca
 4 HN19|NGS    100.0%    attaaaattaataaattaatataaaataaaataaaatataacagaatatatttattaaaacttaatttattaaaatttccaca
 5 HN22|Sanger  73.8%    attaaaattaataaattaatataaaataaaacaaaatataacagaatatatttattaaaatttaatttattaaaatttccaca
 6 HN22|NGS     73.8%    attaaaattaataaattaatataaaataaaacaaaatataacagaatatatttattaaaatttaatttattaaaatttccaca
 7 HN26|Sanger  74.1%    attaaaattaataaattaatataaaataaaacaaaatataacagaatatatttattaaaatttaatttattaaaatttccaca
 8 HN26|NGS     74.1%    attaaaattaataaattaatataaaataaaacaaaatataacagaatatatttattaaaatttaatttattaaaatttccaca
 9 HN6|Sanger   74.1%    attaaaattaataaattaatataaaataaaacaaaatataacagaatatatttattaaaatttaatttattaaaatttccaca
10 HN6|NGS      74.1%    attaaaattaataaattaatataaaataaaacaaaatataacagaatatatttattaaaatttaatttattaaaatttccaca
11 RJ30|Sanger  73.9%    attaaaattaataaattaatataaaataaaacaaaatataacagaatatatttattaaaatttaatttattaaaatttccaca
12 RJ30|NGS     73.9%    attaaaattaataaattaatataaaataaaacaaaatataacagaatatatttattaaaatttaatttattaaaatttccaca
13 RJ26|Sanger  73.9%    attaaaattaataaattaatataaaataaaacaaaatataacagaatatatttattaaaatttaatttattaaaatttccaca
14 RJ26|NGS     73.9%    attaaaattaataaattaatataaaataaaacaaaatataacagaatatatttattaaaatttaatttattaaaatttccaca
15 RJ25|Sanger  73.9%    attaaaattaataaattaatataaaataaaacaaaatataacagaatatatttattaaaatttaatttattaaaatttccaca
16 RJ25|NGS     73.9%    attaaaattaataaattaatataaaataaaacaaaatataacagaatatatttattaaaatttaatttattaaaatttccaca
17 RJ22|Sanger  73.9%    attaaaattaataaattaatataaaataaaacaaaatataacagaatatatttattaaaatttaatttattaaaatttccaca
18 RJ22|NGS     73.9%    attaaaattaataaattaatataaaataaaacaaaatataacagaatatatttattaaaatttaatttattaaaatttccaca
19 RJ17|Sanger  73.9%    attaaaattaataaattaatataaaataaaacaaaatataacagaatatatttattaaaatttaatttattaaaatttccaca
20 RJ17|NGS     73.9%    attaaaattaataaattaatataaaataaaacaaaatataacagaatatatttattaaaatttaatttattaaaatttccaca
21 RJ12|Sanger  73.9%    attaaaattaataaattaatataaaataaaacaaaatataacagaatatatttattaaaatttaatttattaaaatttccaca
22 RJ12|NGS     73.9%    attaaaattaataaattaatataaaataaaacaaaatataacagaatatatttattaaaatttaatttattaaaatttccaca
23 RJ3|Sanger   73.9%    attaaaattaataaattaatataaaataaaacaaaatataacagaatatatttattaaaatttaatttattaaaatttccaca
24 RJ3|NGS      73.9%    attaaaattaataaattaatataaaataaaacaaaatataacagaatatatttattaaaatttaatttattaaaatttccaca
25 RJ2|Sanger   73.9%    attaaaattaataaattaatataaaataaaacaaaatataacagaatatatttattaaaatttaatttattaaaatttccaca
26 RJ2|NGS      73.9%    attaaaattaataaattaatataaaataaaacaaaatataacagaatatatttattaaaatttaatttattaaaatttccaca
27 RJ10|Sanger  73.9%    attaaaattaataaattaatataaaataaaacaaaatataacagaatatatttattaaaatttaatttattaaaatttccaca
28 RJ10|NGS     73.9%    attaaaattaataaattaatataaaataaaacaaaatataacagaatatatttattaaaatttaatttattaaaatttccaca
```

SF21. Mitochondrial tRNA^{Leu}-cox2 intergenic sequence alignment

Sanger sequence mitotypess aligned against their equivalent reconstructed from NGS reads aligned to reference mitotypes.

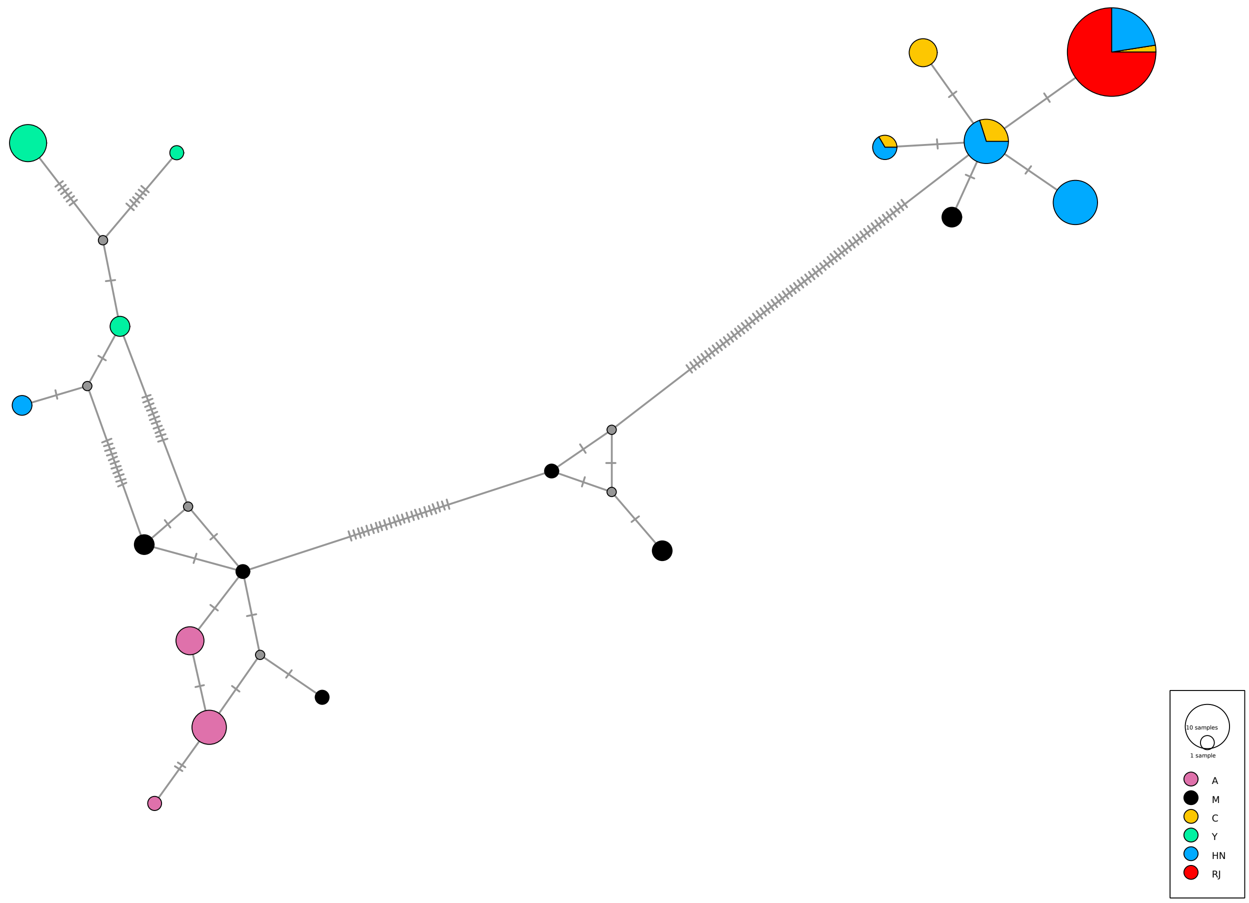

SF22. Haplotype network constructed from mitochondrial tRNA^{Leu}-cox2 intergenic sequence

Reads spanning the intergenic tRNA^{Leu}-cox2 sequence (MT:3413-3626) were retrieved from the BAM files for all bees analysed and aligned to a database of reference mitotype sequences downloaded from GenBank. A fasta sequence was generated from the best alignment for each individual. Sequences were aligned using MAFFT and re-coded such that all 'C' nucleotides were re-coded 'G', and all gaps were re-coded as 'C'. The haplotype network was then constructed using PopArt. Dashes along branches indicate single-nucleotide differences between haplotype sequences.

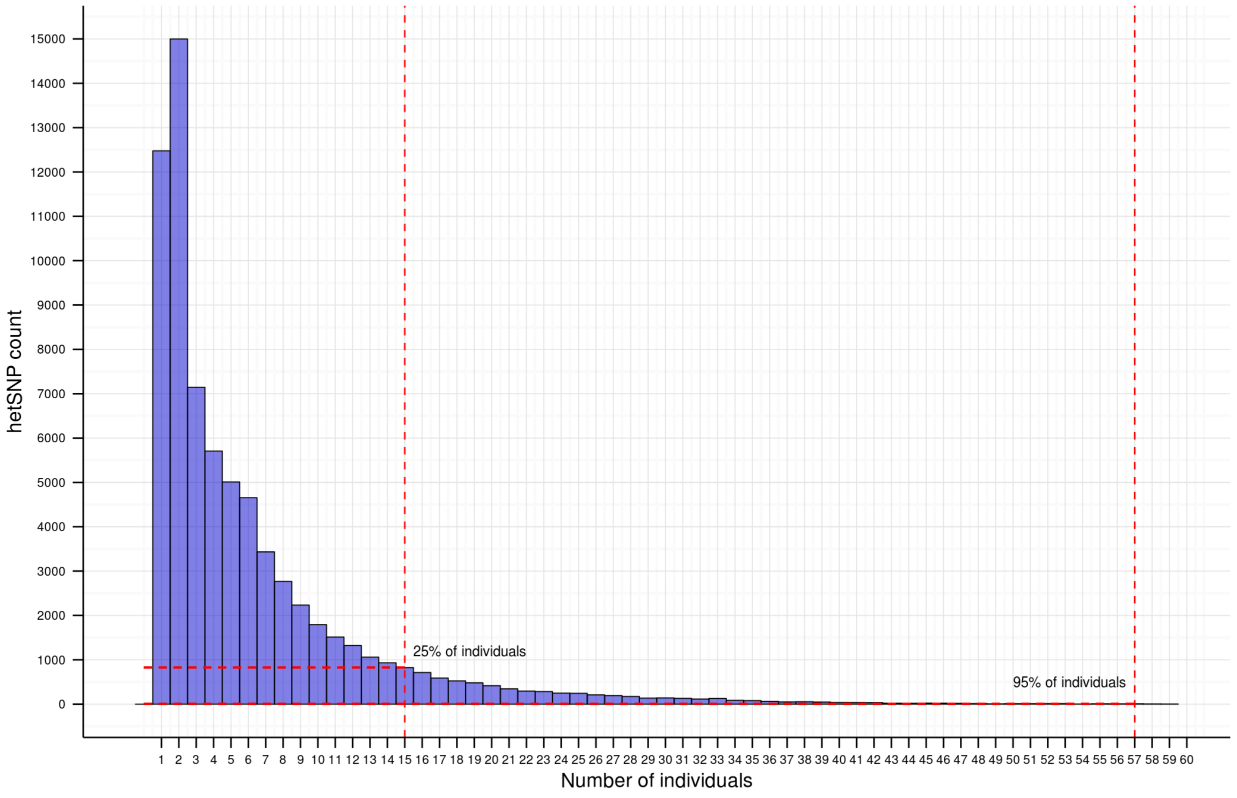

SF23. Histogram of heterozygous SNPs (hetSNPs) prior to clustering

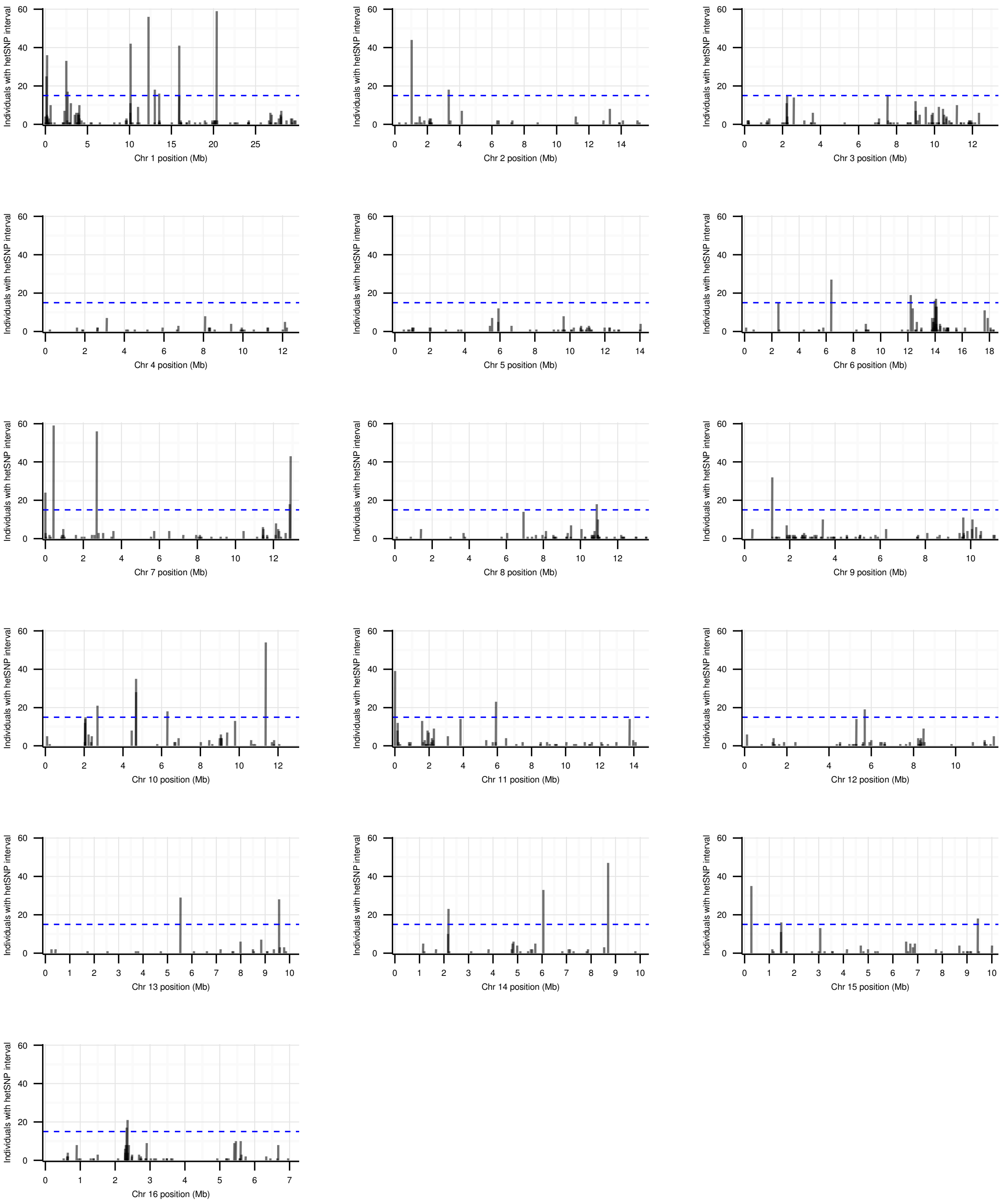

SF24. Distribution of hetSNP clusters

Clusters plotted comprise ≥ 3 SNPs, span ≥ 2 kb and have DP ≥ 3 times the average. Horizontal line marks threshold at which ≥15 individuals possess the hetSNP interval.

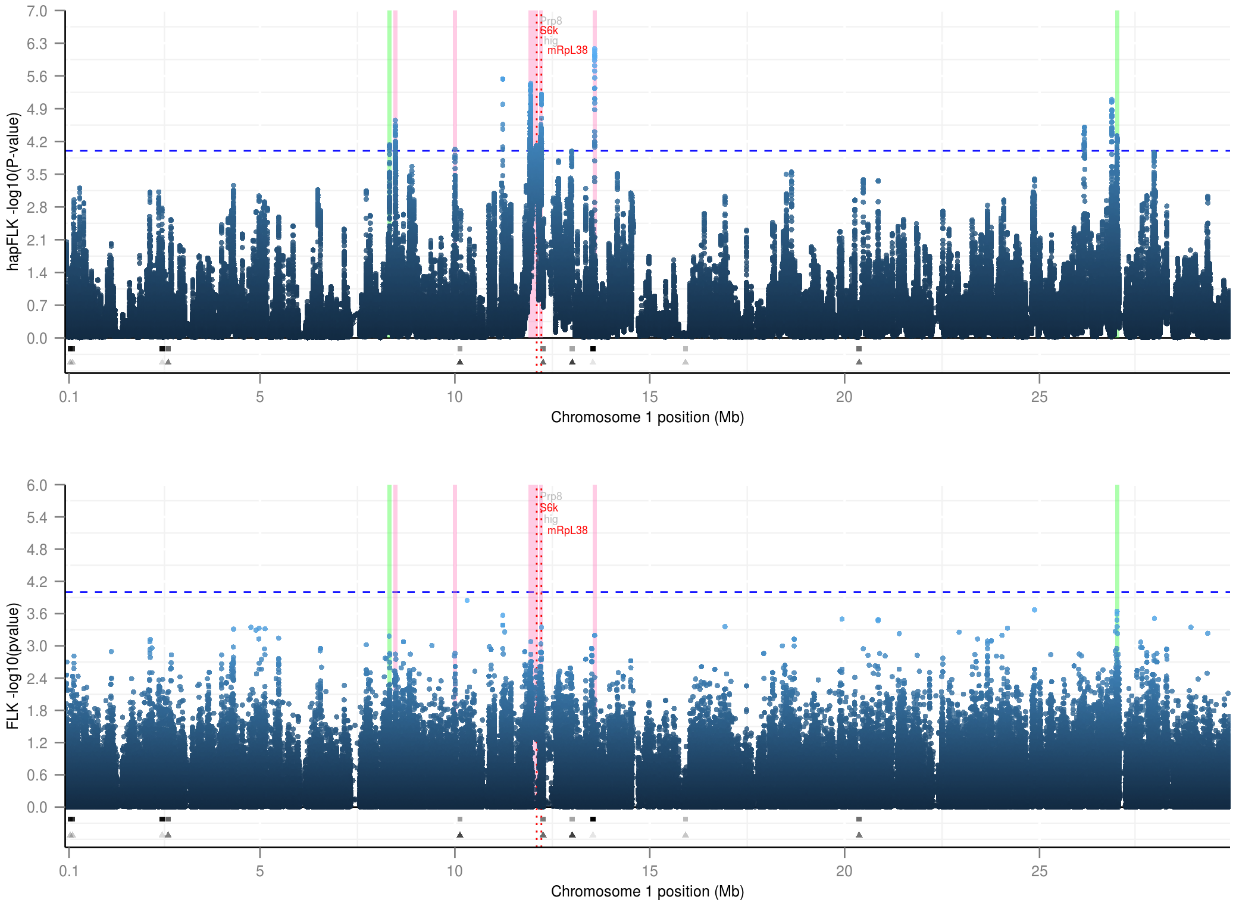

SF25. HapFLK and FLK plots of chromosome 1

Horizontal dashed line indicates significane threshold of 10^{-4}. Vertical yellow and red highlights indicate putative selection signature in HN and RJ populations, respectively. Genes in blue text are discussed in the main text with regards to their GO terms, whilst genes in red text are also supported by previous studies investigating differential gene expression. Lower track indicates hetSNP clusters identified in HN (triangle) and RJ (square) populations, respectively, where the level of transparency is relative to the frequency of the cluster in the population.

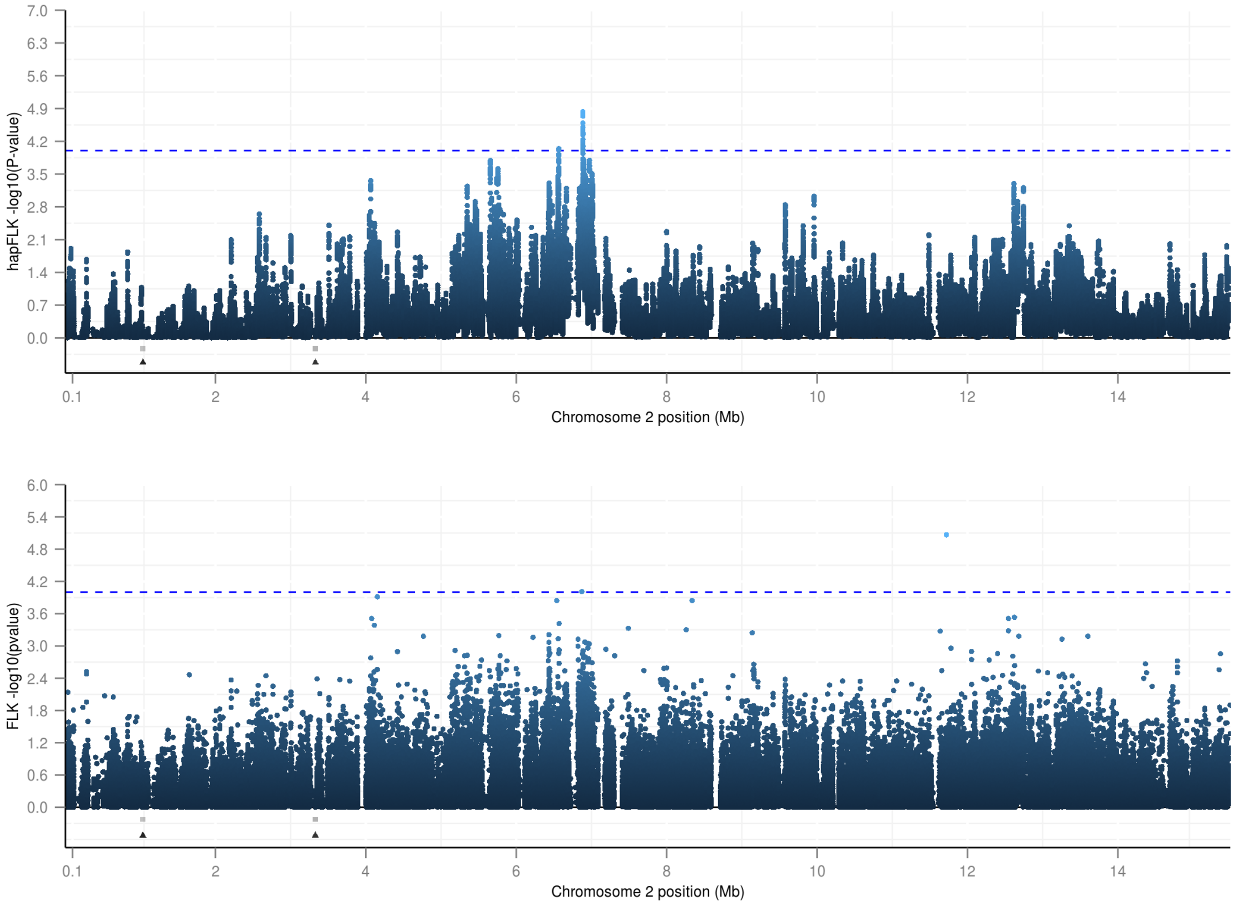

SF26. HapFLK and FLK plots of chromosome 2

Horizontal dashed line indicates significane threshold of 10^{-4}. Vertical yellow and red highlights indicate putative selection signature in HN and RJ populations, respectively. Genes in blue text are discussed in the main text with regards to their GO terms, whilst genes in red text are also supported by previous studies investigating differential gene expression. Lower track indicates hetSNP clusters identified in HN (triangle) and RJ (square) populations, respectively, where the level of transparency is relative to the frequency of the cluster in the population.

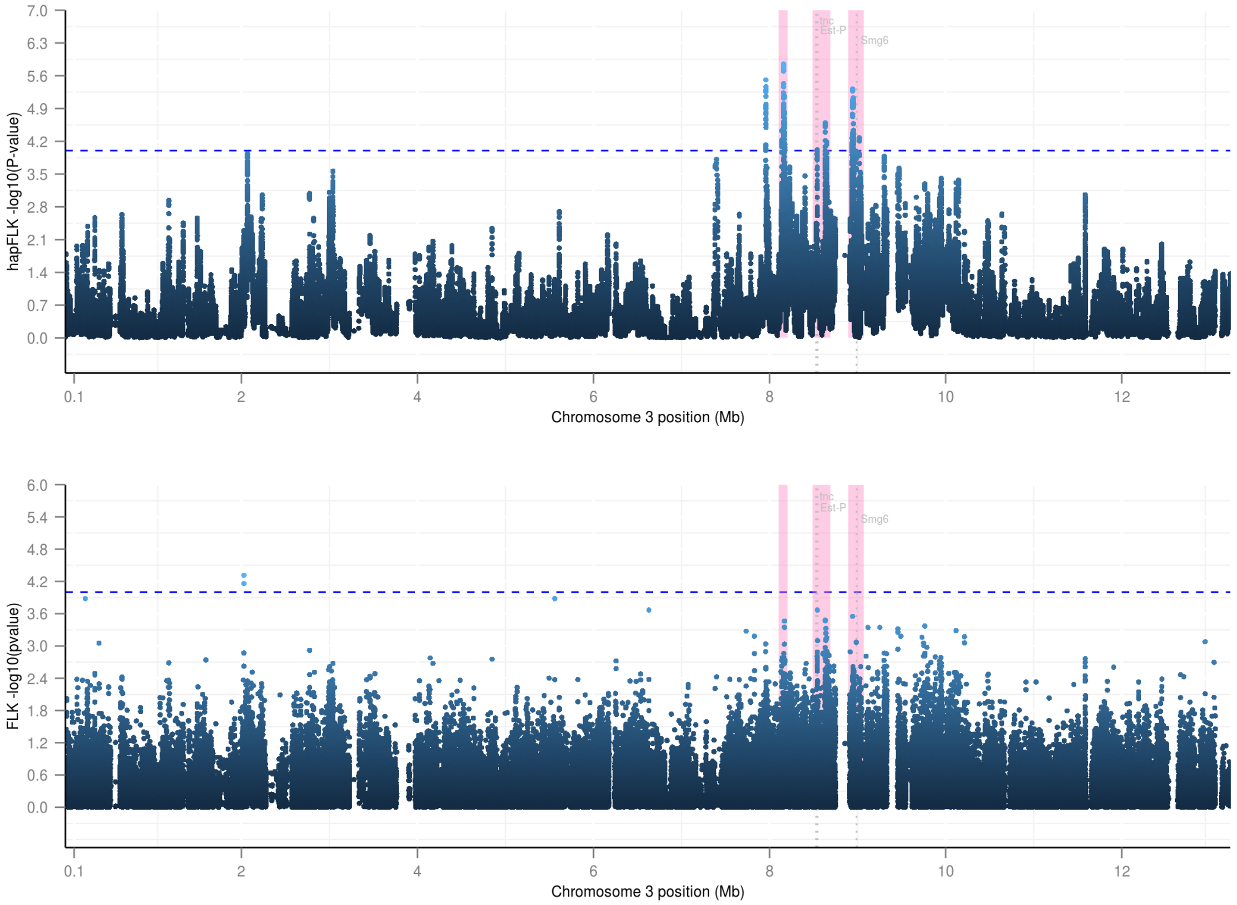

SF27. HapFLK and FLK plots of chromosome 3

Horizontal dashed line indicates significane threshold of 10^{-4}. Vertical yellow and red highlights indicate putative selection signature in HN and RJ populations, respectively. Genes in blue text are discussed in the main text with regards to their GO terms, whilst genes in red text are also supported by previous studies investigating differential gene expression. Lower track indicates hetSNP clusters identified in HN (triangle) and RJ (square) populations, respectively, where the level of transparency is relative to the frequency of the cluster in the population.

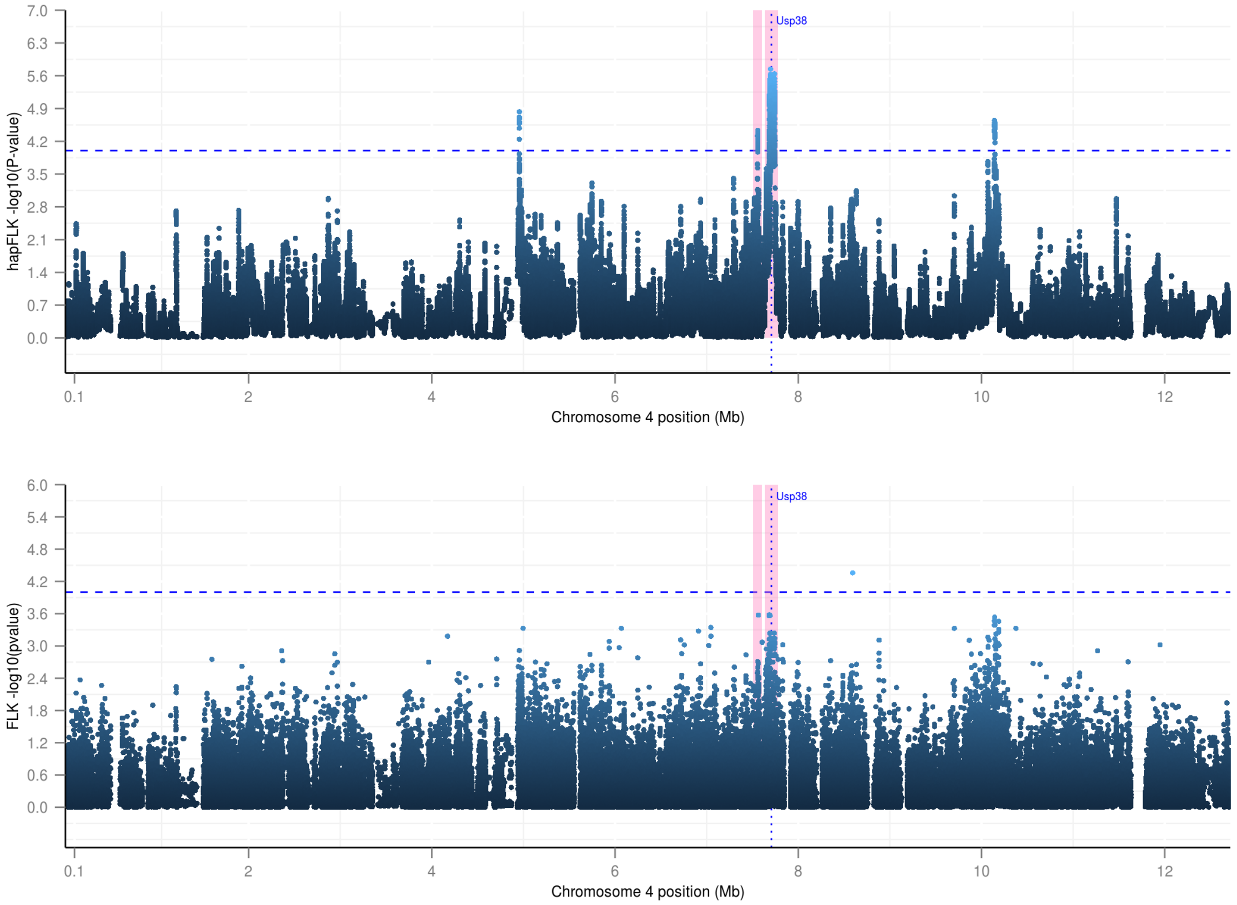

SF28. HapFLK and FLK plots of chromosome 4

Horizontal dashed line indicates significane threshold of 10^{-4}. Vertical yellow and red highlights indicate putative selection signature in HN and RJ populations, respectively. Genes in blue text are discussed in the main text with regards to their GO terms, whilst genes in red text are also supported by previous studies investigating differential gene expression. Lower track indicates hetSNP clusters identified in HN (triangle) and RJ (square) populations, respectively, where the level of transparency is relative to the frequency of the cluster in the population.

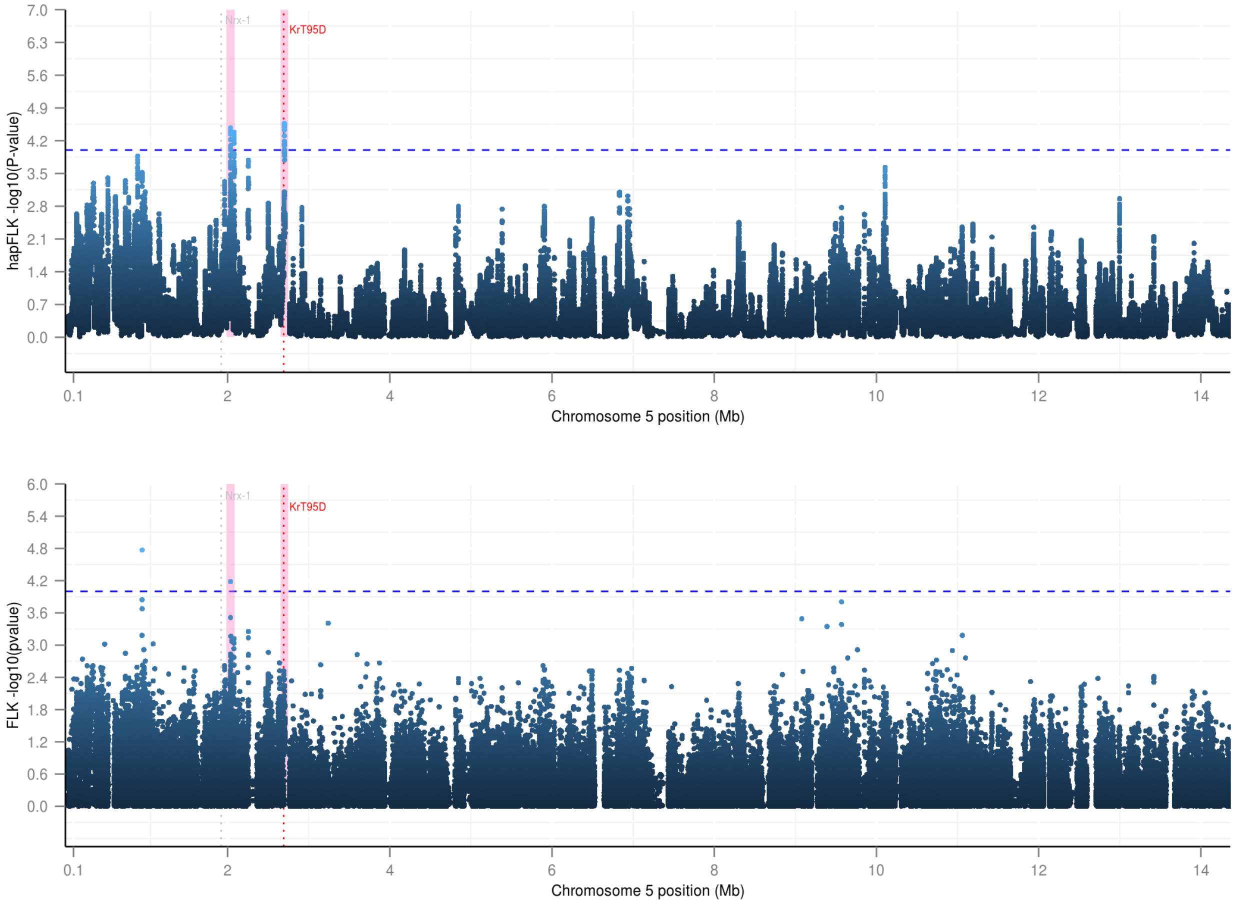

SF29. HapFLK and FLK plots of chromosome 5

Horizontal dashed line indicates significane threshold of 10^{-4}. Vertical yellow and red highlights indicate putative selection signature in HN and RJ populations, respectively. Genes in blue text are discussed in the main text with regards to their GO terms, whilst genes in red text are also supported by previous studies investigating differential gene expression. Lower track indicates hetSNP clusters identified in HN (triangle) and RJ (square) populations, respectively, where the level of transparency is relative to the frequency of the cluster in the population.

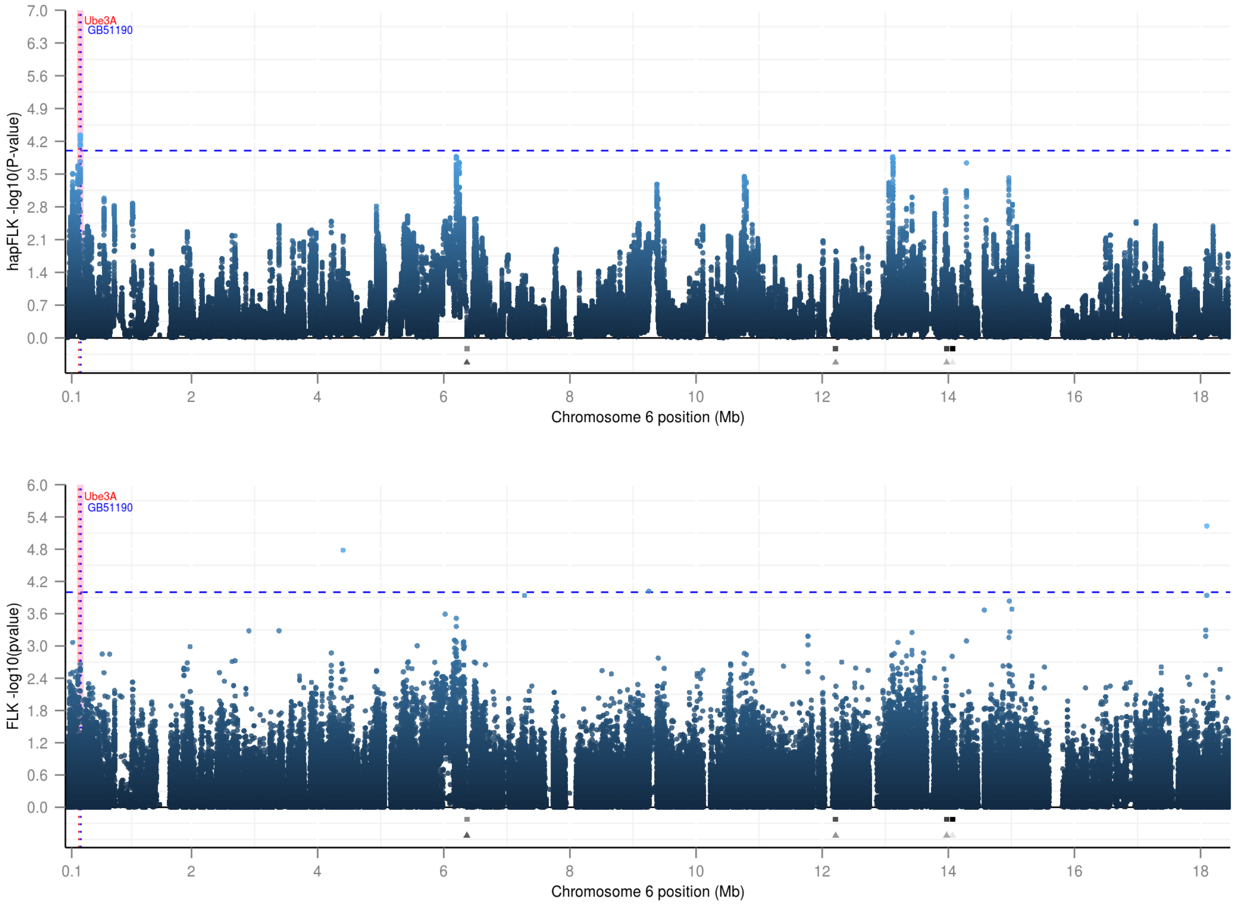

SF30. HapFLK and FLK plots of chromosome 6

Horizontal dashed line indicates significane threshold of 10^{-4}. Vertical yellow and red highlights indicate putative selection signature in HN and RJ populations, respectively. Genes in blue text are discussed in the main text with regards to their GO terms, whilst genes in red text are also supported by previous studies investigating differential gene expression. Lower track indicates hetSNP clusters identified in HN (triangle) and RJ (square) populations, respectively, where the level of transparency is relative to the frequency of the cluster in the population.

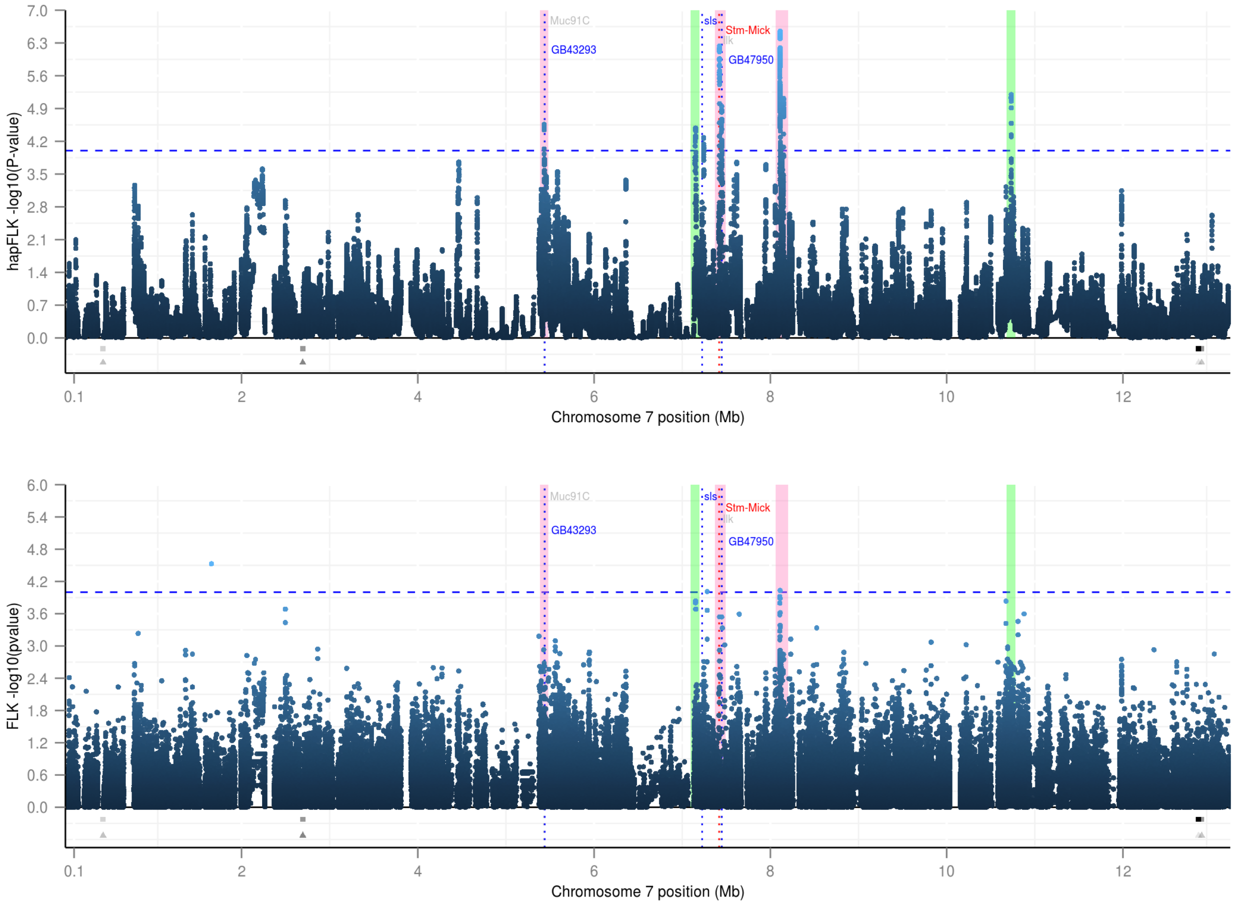

SF31. HapFLK and FLK plots of chromosome 7

Horizontal dashed line indicates significane threshold of 10^{-4}. Vertical yellow and red highlights indicate putative selection signature in HN and RJ populations, respectively. Genes in blue text are discussed in the main text with regards to their GO terms, whilst genes in red text are also supported by previous studies investigating differential gene expression. Lower track indicates hetSNP clusters identified in HN (triangle) and RJ (square) populations, respectively, where the level of transparency is relative to the frequency of the cluster in the population.

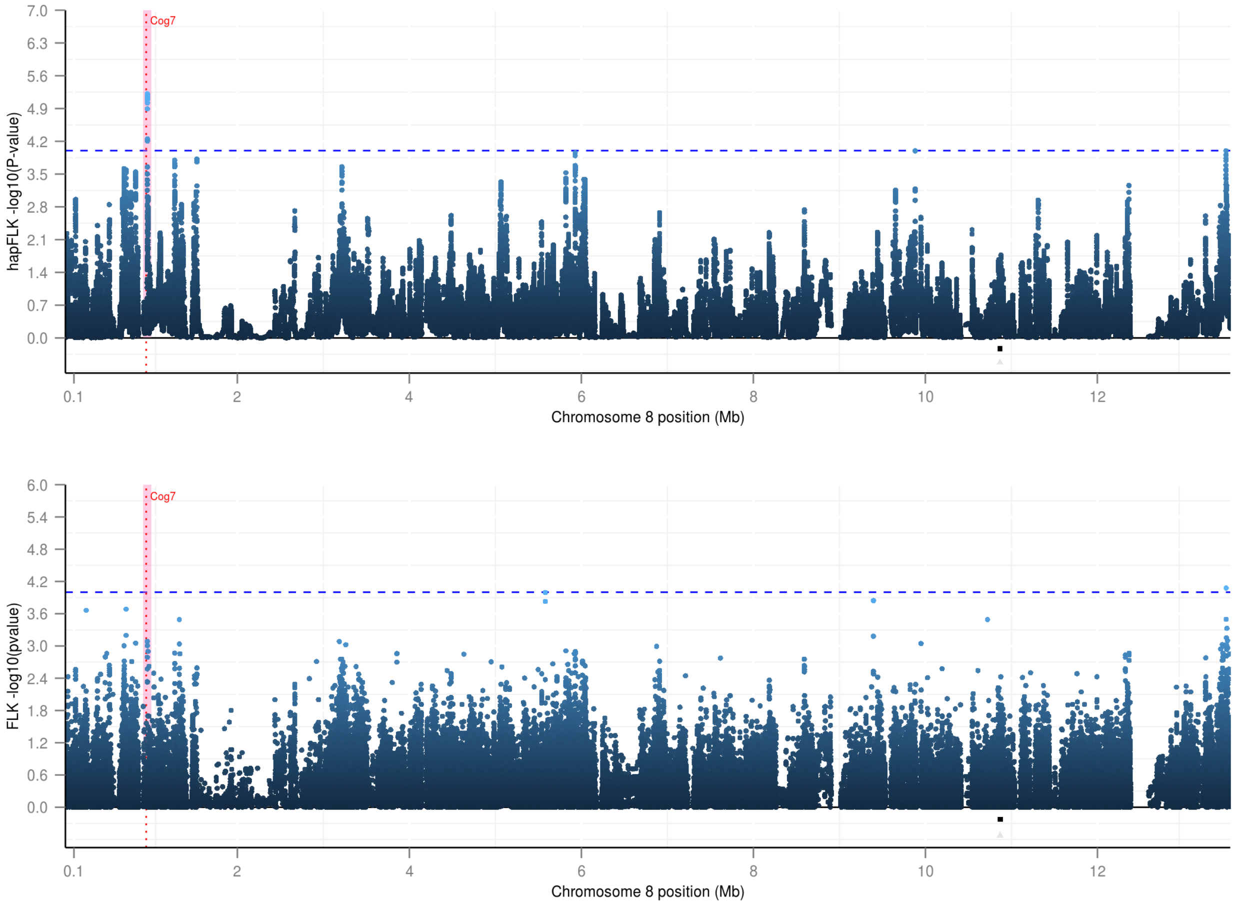

SF32. HapFLK and FLK plots of chromosome 8

Horizontal dashed line indicates significane threshold of 10^{-4}. Vertical yellow and red highlights indicate putative selection signature in HN and RJ populations, respectively. Genes in blue text are discussed in the main text with regards to their GO terms, whilst genes in red text are also supported by previous studies investigating differential gene expression. Lower track indicates hetSNP clusters identified in HN (triangle) and RJ (square) populations, respectively, where the level of transparency is relative to the frequency of the cluster in the population.

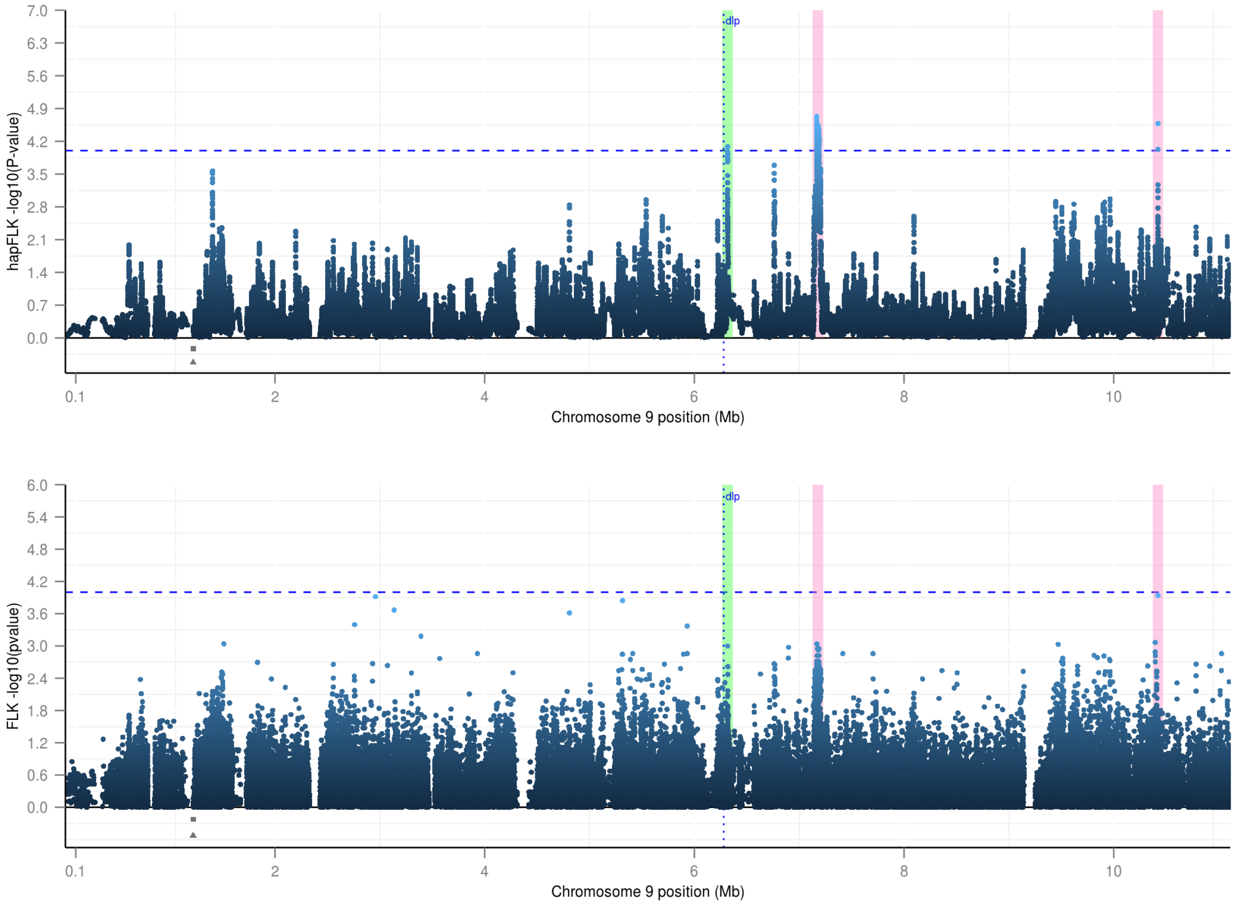

SF33. HapFLK and FLK plots of chromosome 9

Horizontal dashed line indicates significane threshold of 10^{-4}. Vertical yellow and red highlights indicate putative selection signature in HN and RJ populations, respectively. Genes in blue text are discussed in the main text with regards to their GO terms, whilst genes in red text are also supported by previous studies investigating differential gene expression. Lower track indicates hetSNP clusters identified in HN (triangle) and RJ (square) populations, respectively, where the level of transparency is relative to the frequency of the cluster in the population.

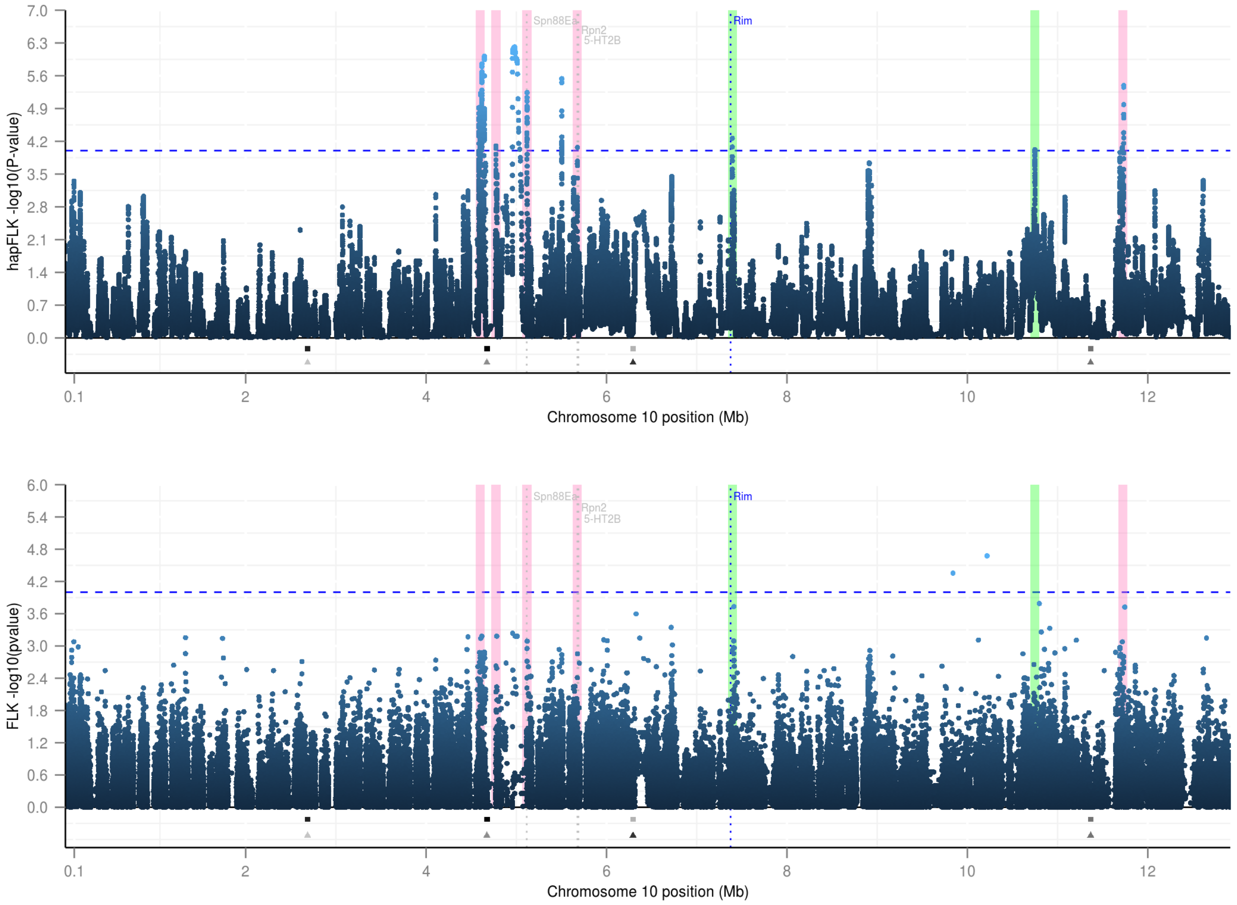

SF34. HapFLK and FLK plots of chromosome 10

Horizontal dashed line indicates significane threshold of 10^{-4}. Vertical yellow and red highlights indicate putative selection signature in HN and RJ populations, respectively. Genes in blue text are discussed in the main text with regards to their GO terms, whilst genes in red text are also supported by previous studies investigating differential gene expression. Lower track indicates hetSNP clusters identified in HN (triangle) and RJ (square) populations, respectively, where the level of transparency is relative to the frequency of the cluster in the population.

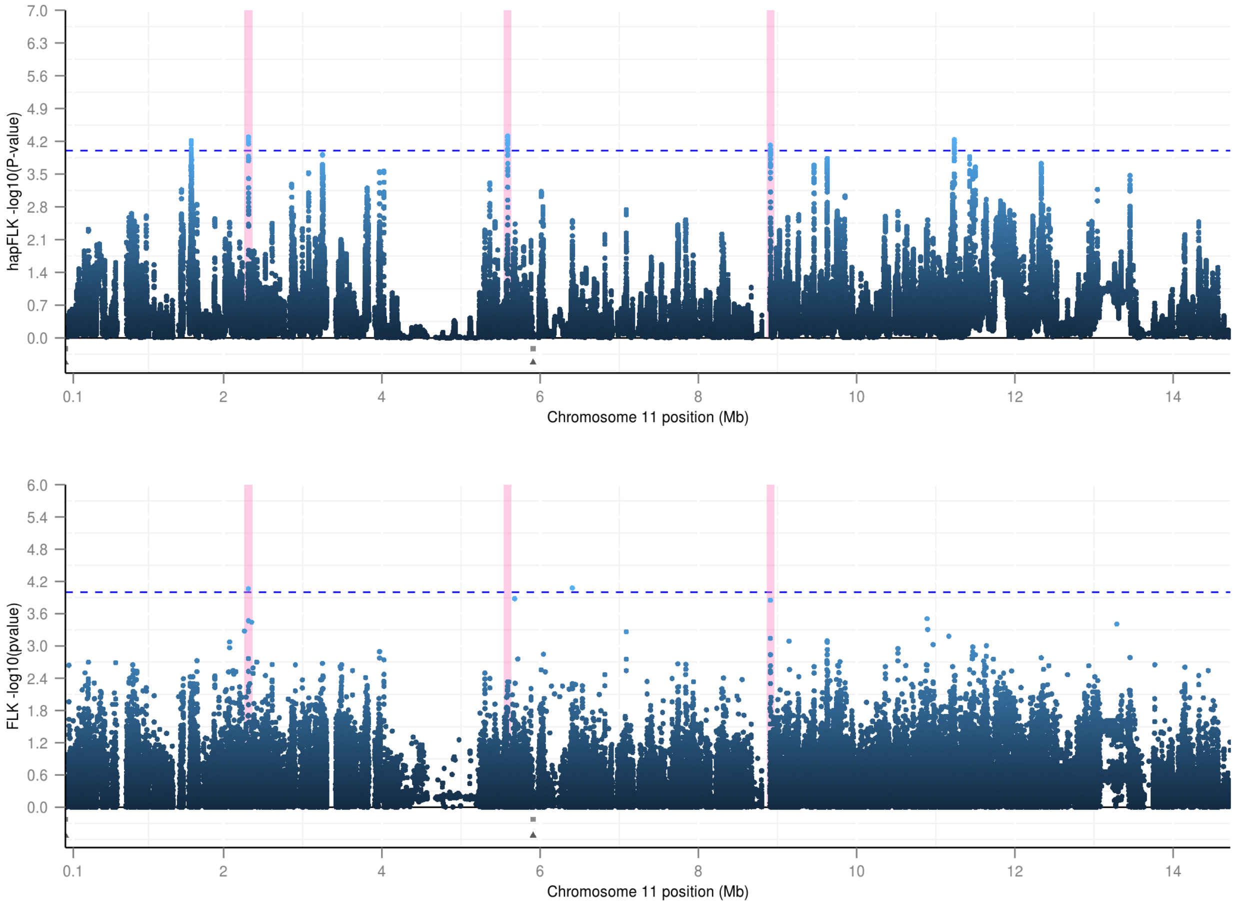

SF35. HapFLK and FLK plots of chromosome 11

Horizontal dashed line indicates significane threshold of 10^{-4}. Vertical yellow and red highlights indicate putative selection signature in HN and RJ populations, respectively. Genes in blue text are discussed in the main text with regards to their GO terms, whilst genes in red text are also supported by previous studies investigating differential gene expression. Lower track indicates hetSNP clusters identified in HN (triangle) and RJ (square) populations, respectively, where the level of transparency is relative to the frequency of the cluster in the population.

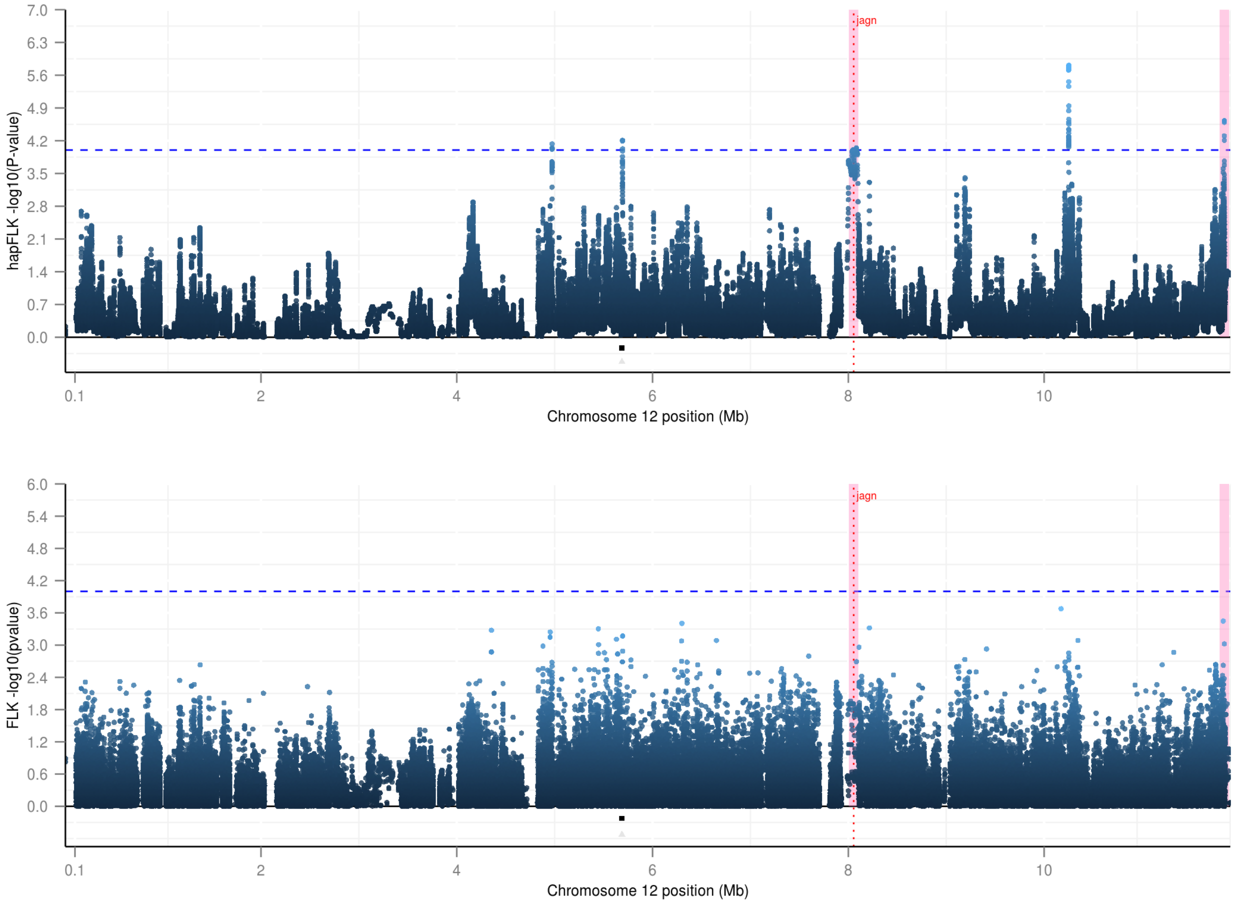

SF36. HapFLK and FLK plots of chromosome 12

Horizontal dashed line indicates significane threshold of 10^{-4}. Vertical yellow and red highlights indicate putative selection signature in HN and RJ populations, respectively. Genes in blue text are discussed in the main text with regards to their GO terms, whilst genes in red text are also supported by previous studies investigating differential gene expression. Lower track indicates hetSNP clusters identified in HN (triangle) and RJ (square) populations, respectively, where the level of transparency is relative to the frequency of the cluster in the population.

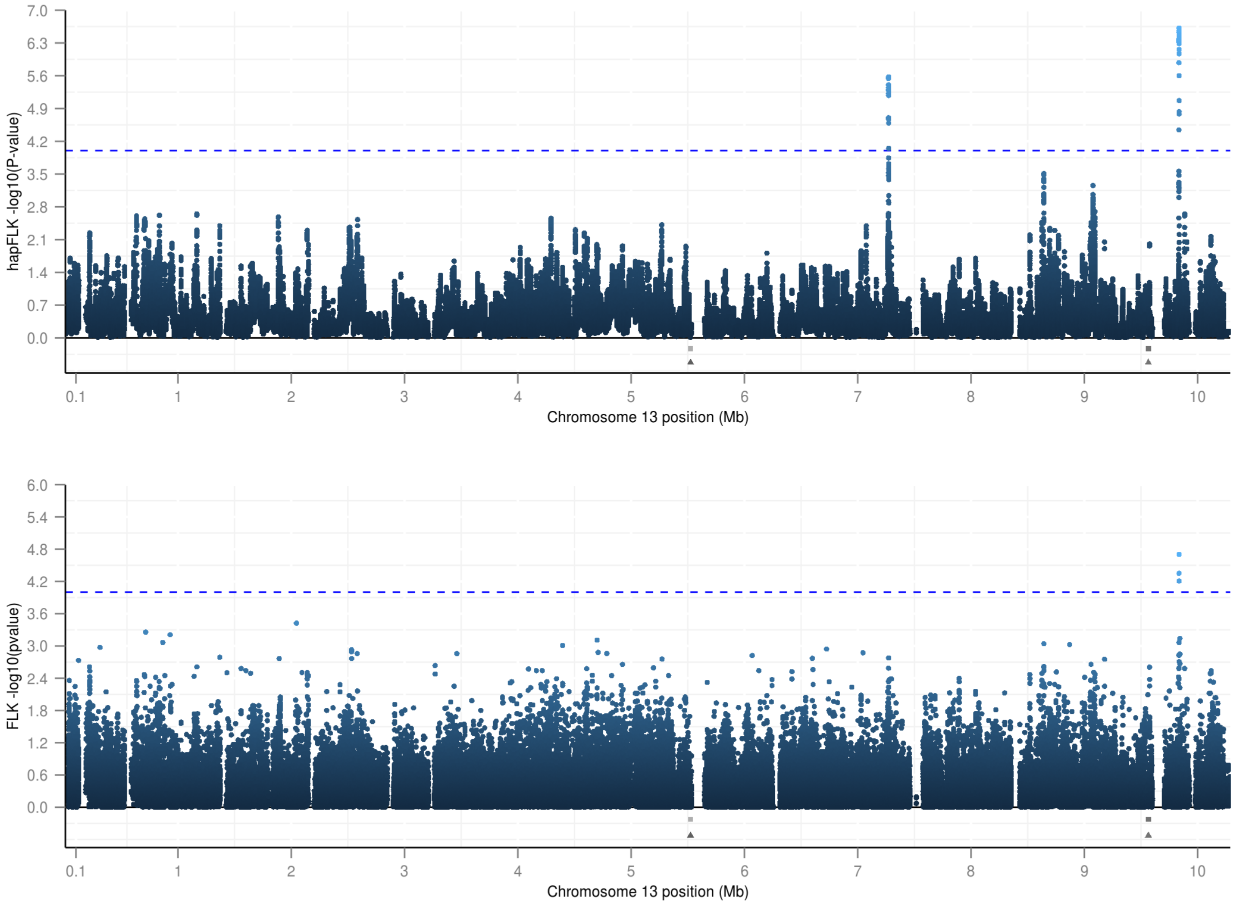

SF37. HapFLK and FLK plots of chromosome 13

Horizontal dashed line indicates significane threshold of 10^{-4}. Vertical yellow and red highlights indicate putative selection signature in HN and RJ populations, respectively. Genes in blue text are discussed in the main text with regards to their GO terms, whilst genes in red text are also supported by previous studies investigating differential gene expression. Lower track indicates hetSNP clusters identified in HN (triangle) and RJ (square) populations, respectively, where the level of transparency is relative to the frequency of the cluster in the population.

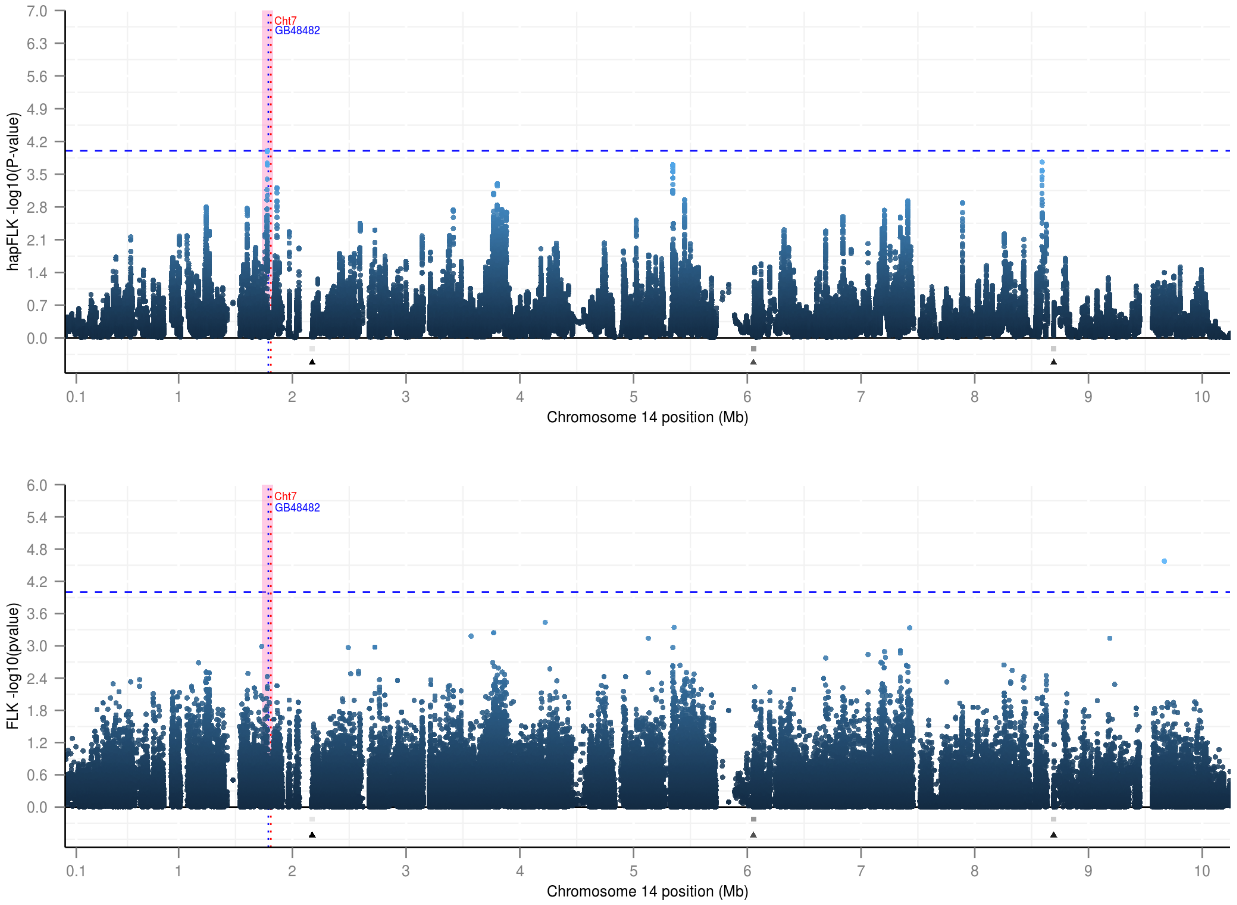

SF38. HapFLK and FLK plots of chromosome 14

Horizontal dashed line indicates significane threshold of 10^{-4}. Vertical yellow and red highlights indicate putative selection signature in HN and RJ populations, respectively. Genes in blue text are discussed in the main text with regards to their GO terms, whilst genes in red text are also supported by previous studies investigating differential gene expression. Lower track indicates hetSNP clusters identified in HN (triangle) and RJ (square) populations, respectively, where the level of transparency is relative to the frequency of the cluster in the population.

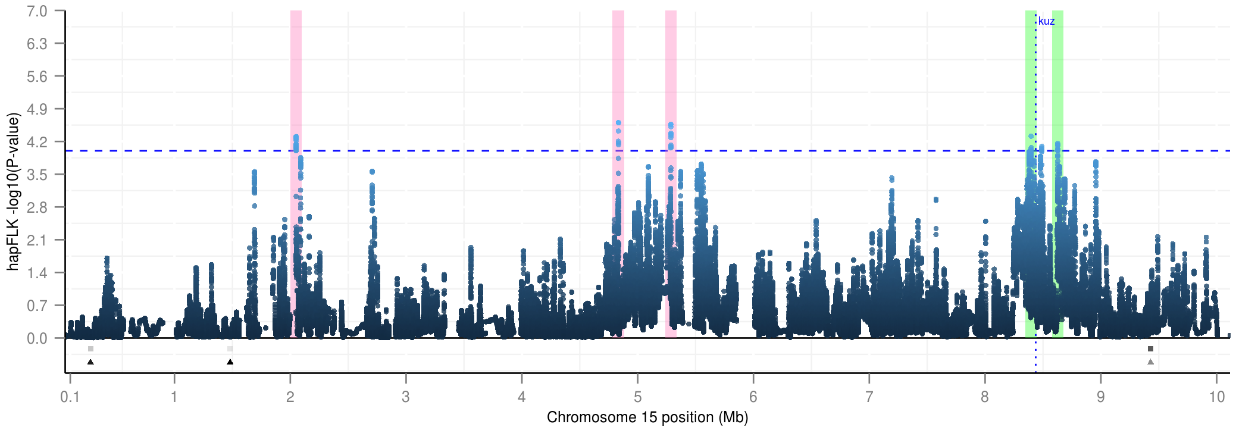

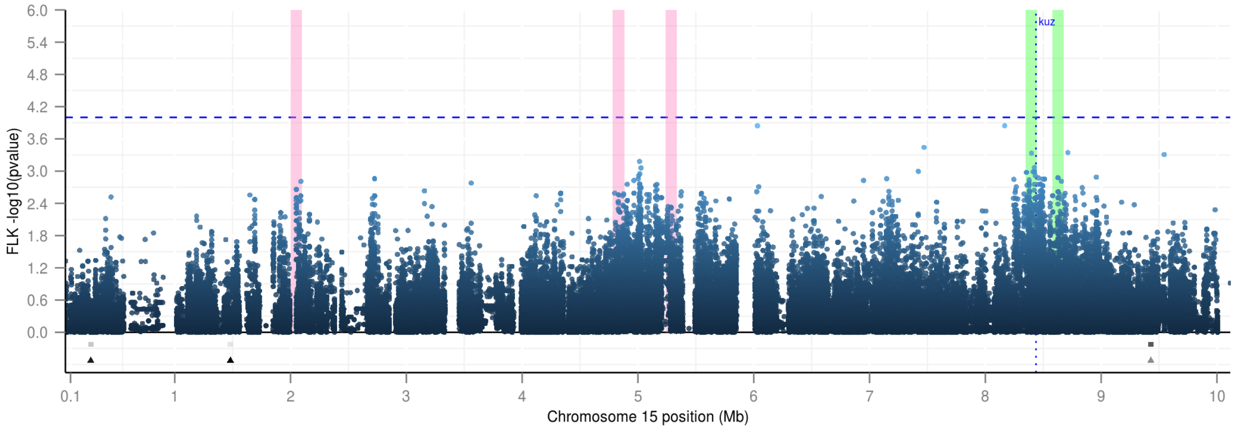

SF39. HapFLK and FLK plots of chromosome 15

Horizontal dashed line indicates significane threshold of 10^{-4}. Vertical yellow and red highlights indicate putative selection signature in HN and RJ populations, respectively. Genes in blue text are discussed in the main text with regards to their GO terms, whilst genes in red text are also supported by previous studies investigating differential gene expression. Lower track indicates hetSNP clusters identified in HN (triangle) and RJ (square) populations, respectively, where the level of transparency is relative to the frequency of the cluster in the population.

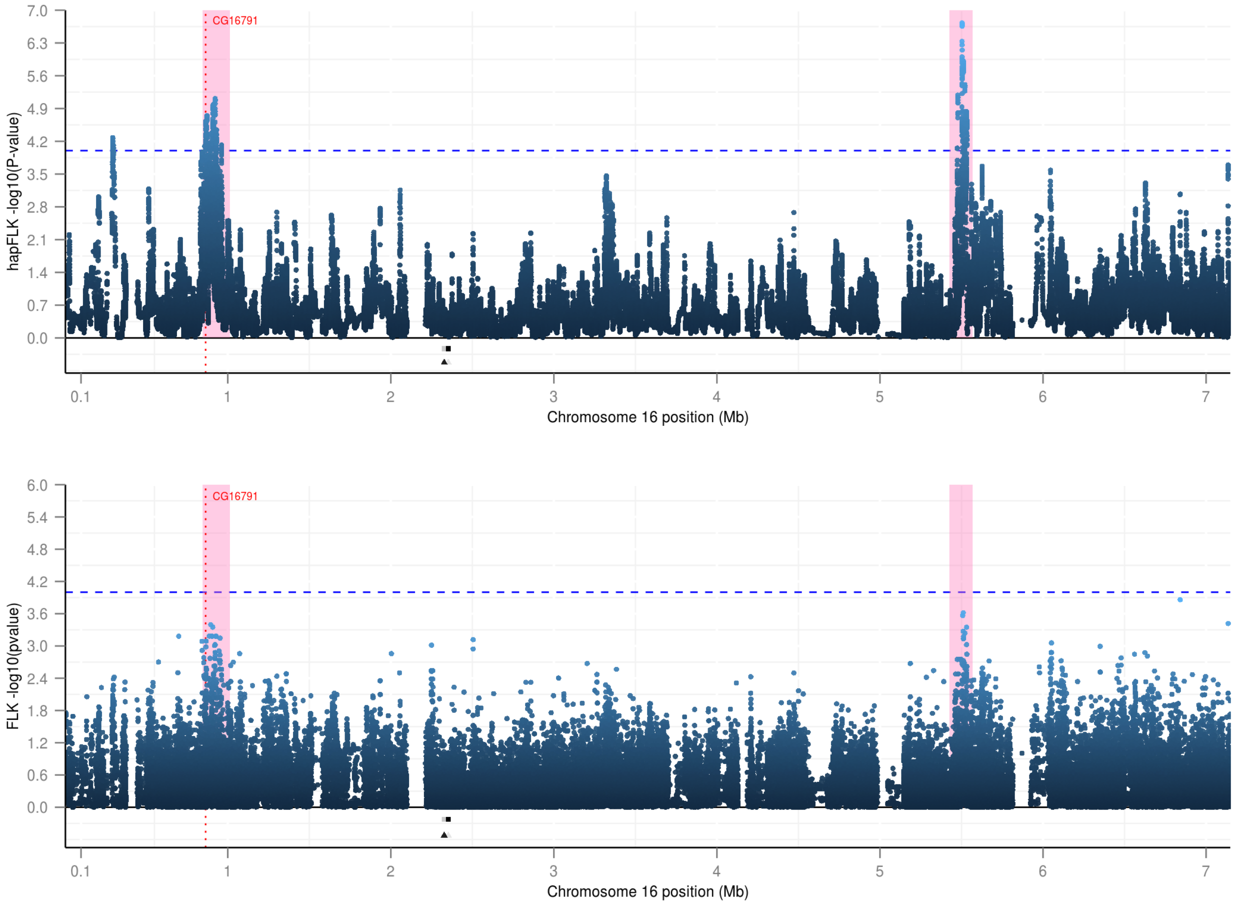

SF40. HapFLK and FLK plots of chromosome 16

Horizontal dashed line indicates significane threshold of 10^{-4}. Vertical yellow and red highlights indicate putative selection signature in HN and RJ populations, respectively. Genes in blue text are discussed in the main text with regards to their GO terms, whilst genes in red text are also supported by previous studies investigating differential gene expression. Lower track indicates hetSNP clusters identified in HN (triangle) and RJ (square) populations, respectively, where the level of transparency is relative to the frequency of the cluster in the population.
